# Electrocatalyst Performances in Direct Alcohol Fuel Cells: Defect Engineering Protocols, Electrocatalytic Pathways, Key Parameters for Improvement, and Breakthroughs on the Horizon

**DOI:** 10.1002/smsc.202300057

**Published:** 2023-11-27

**Authors:** Thabo Matthews, Siyabonga Patrick Mbokazi, Tarekegn Heliso Dolla, Sandile Surprise Gwebu, Kudzai Mugadza, Katlego Raseruthe, Ludwe Luther Sikeyi, Kayode Adesina Adegoke, Oluwaseyi Damilare Saliu, Abolanle Saheed Adekunle, Patrick Ndungu, Nobanathi Wendy Maxakato

**Affiliations:** ^1^ Department of Chemical Sciences University of Johannesburg Doornfontein 2028 South Africa; ^2^ Department of Chemistry Wolaita Sodo University P.O.Box 138 Wolaita Sodo Ethiopia; ^3^ Department of Chemical Engineering University of Capetown Rondebosch Cape Town 770 South Africa; ^4^ Institute of Materials Science, Processing and Engineering Technology Chinhoyi University of Technology P. Bag 7724 Chinhoyi Zimbabwe; ^5^ Molecular Sciences Institute School of Chemistry University of Witwatersrand Braamfontein 2050 South Africa; ^6^ Department of Chemistry Obafemi Awolowo University PMB 220005 Osun Nigeria; ^7^ Department of Chemistry University of Pretoria Pretoria 0001 South Africa

**Keywords:** defects, electrocatalysts, electrocatalytic oxidation, electrocatalytic pathways, electrocatalytic performances, electrocatalytic reduction, fuel cells

## Abstract

In direct alcohol fuel cells (DAFCs), energy conversion co‐occurs at the anode (alcohol oxidation reaction [AOR]) and cathode (oxygen reduction reaction [ORR]). The sluggishness of AOR and ORR needs highly electrocatalytically active and stable electrocatalysts that boost electrokinetics, which is central in electrocatalysts’ architectural design and modulation. This design entails enhanced engineering synthesis protocols, heteroatomic doping, metallic doping/alloying, and deliberate introduction of defective motifs within the electrocatalyst matrix. The electrocatalyst activity and behavior depend on the electrocatalysts’ nature, type, composition, and reaction media, acidic or alkaline. Alkaline media permits cheap nonplatinum group metals. This review elucidates the roles and electrocatalytic pathways on different AOR and ORR electrocatalysts and outlines the aspects distinguishing ORR in alkaline and acidic media. It gives up‐to‐date and ultramodern strategies, protocols, and underlying mechanisms pointing to the efficacy and efficiency of electrocatalysts. The focus centers on heteroatomic, metallic dopants, defects effects correlated to electrocatalytic properties and experimental and theoretical findings. For the advancement in the field, the present study discusses critical parameters for improving the performances of electrocatalysts for DAFCs and breakthroughs on the horizon. Conclusively, knowledge gaps and prospects of these materials for industrial viability and reigning futuristic research directions are presented.

## Graphical abstract

Electrochemical performance in direct alcohol fuel cells (DAFCs) is influenced by several factors, including deliberately inducing defects (heteroatomic and metallic dopants, defects, dislocations) on electrocatalysts using different engineering methods, and governs electrocatalytic pathways for the oxygen reduction and alcohol oxidation, and the ultimate improvement of DAFCs reactions is fundamental to the industrialization of fuel cell technology.

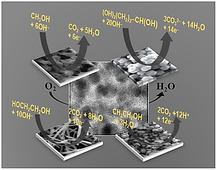

## 1 Introduction

The soaring world population, expected to double by 2050,^[^ ^1^ ^]^ is straining preexisting fossil fuels‐driven energy sources. There is a correlation between the development and growth of humanity based on energy sustainability, so energy is vital in modern societies.^[^ ^2^ ^]^ Energy is the hub where social, socio‐economic, political, and politico‐economic spheres revolve. The provision of energy is currently centered on fossil fuels, which are demerited to land degradation, climatic shifts, coronary diseases, etc. Renewable energy adoption is invincible for a sustainable future. Plausible sustainability means the erection of integrated renewable energy systems. These systems produce energy from replenishable sources, for example, wind, solar, biofuels, and geothermal. As fuel cells (FCs) use biofuels as the anodic fuel, they have drawn the focus on integrating them into energy conversion systems. The typical FC descriptors are 1) electrode–electrolyte contact, 2) separated electron transport and ion transport, and 3) energy transfer processes at the three‐phase boundary.^[^ ^3^ ^]^ FCs are merited to continuous power supply, clean, environmentally benign, and noise free.

The main drivers of the chemical energy conversion in FCs are electrocatalytic reactions necessitated by electrocatalysts. In a FC, there is electro‐oxidation of fuel (FOR) (e.g., hydrogen, H_2_, methanol, CH_3_OH, ethanol, CH_3_CH_2_OH, ethylene glycol, HOCH_2_CH_2_OH, ammonia, NH_3,_ etc.) at the anode and electroreduction of oxygen reduction reaction (ORR) at the cathode. The alcohol oxidation reaction (AOR) and ORR are sluggish and need boosting of the kinetics from the electrocatalyst. Thus, electrocatalysis is vital in the development of FCs. Electrocatalysis houses electrocatalyst engineering (e.g., introducing defects, heteroatoms, metallic dopants), electrocatalytic routes modulation, and mechanism evaluation. Improving the electrocatalytic performance entails well‐optimized, integrated compounded electrocatalysis branches. The core guide for better electrocatalysis also emanates from the electrocatalyst's synthesis protocols and architectural design.

Generally, the end goal for electrocatalysis is to have vast exposed electrochemically active sites, which are achievable through defect engineering, heteroatom doping, and metallic doping. These strategies typically alter the active sites' chemical environment modulation of the electronic structure, which inherently amplifies the charge transfer and electron mobilities.^[^ ^4^, ^5^ ^]^ Metallic‐based (platinum group metals [PGMs], e.g., Pd, Pt, Ru,^[^ ^6^, ^7^, ^8^ ^]^ and non‐PGMs, e.g., carbon‐based,^[^ ^9^, ^10^, ^11^ ^]^ metal oxides,^[^ ^12^, ^13^, ^14^, ^15^, ^16^ ^]^ nitrides)^[^ ^17^, ^18^, ^19^ ^]^ have been investigated. However, because of the absence of highly sophisticated characterization techniques in the past, electrocatalytic contribution effects of defects and doping on electrocatalysts’ performance have not been fully elucidated. Various cutting‐edge technologies have recently investigated the extent of defects and dopants' impact and role in electrocatalysis. These specialized techniques include but are not limited to electron energy loss spectroscopy (EELS), X‐ray photoelectron spectroscopy (XPS), and X‐ray absorption spectroscopy (XAS), which reveal information about the chemical environment, coordination chemistry, chemical bonding, surface shape, electronic structural distortion, and distribution.^[^ ^20^ ^]^ This information helps comprehend and correlate electrocatalysts' reaction mechanisms with performance, giving more light to the structure–activity electrocatalytic mechanism relationship.

The overall adaptation of these technologies is based on the successful engineering of: 1) electrocatalysts to aid in more significant electrode kinetics and 2) electrocatalysts to support material optimization. There is a need for systematic studies to reveal the complex inﬂuential nature of carbon materials' structural and morphological performance in FCs. These studies will develop a new class of structural architecture tailormade materials with improved catalytic properties, low‐PGM contents, and high durability. The architectural electrocatalyst design has prompted the development of intelligent nanomaterials for the electrocatalytic ORR and AOR.

This review generally focuses on the role of intelligent nanomaterials electrocatalysts in the cathode and anode electrocatalytic processes. The electrocatalytic processes in a FC are mainly fuel electro‐oxidation and oxidant electroreduction. The typical fuel is hydrogen, but some classes of fuel with a high hydrogen density have emerged, for example, methanol,^[^ ^21^, ^22^ ^]^ ethanol,^[^ ^23^, ^24^ ^]^ ethylene glycol,^[^ ^25^ ^]^ and glycerol.^[^ ^26^ ^]^ The fast‐paced development of DAFCs is mainly because of lower cost and environmental friendliness, a renewable resource. Electrocatalysis is necessary for energy conversion in an FC. Electrocatalysis is essential as it accelerates the electrochemical reactions occurring at the surface of the electrocatalyst electrode. Thus, first, we elucidate engineering defect protocols, electrocatalytic pathways, defect categories, fabricating strategies, and defect sites for regulating the electronic structures linked to electrocatalytic activity. These include the reactants’ chemical interactions resulting in intermediates and product formation. Thus, electrocatalysts are very important in developing FCs. Thus, the engineering of smart nanomaterial‐based electrocatalysts is of paramount importance. These should have advanced nanostructures to realize high electrocatalytic performance. To lower the reaction activation energy, nanostructures must contain PGMs, e.g., palladium, ruthenium, etc., to reduce the reaction activation energy.^[^ ^27^ ^]^ As the electrocatalytic reactions in a FC are different, optimizing the catalytic performance requires different architecture for enhanced performance.^[^ ^28^ ^]^ Finally, we presented some knowledge gaps and prospects of the electrocatalysts for industrial viability, the futuristic research direction, key parameters for improvement, breakthroughs on the horizon, conclusions, and outlooks.

## 2 Basic Engineering Defect Protocols for Fuel Cell Electrocatalysis

### 2.1 Definition and Theory of Defects for Designing Electrocatalysts

In 1897, Tate v. Latham and Son defined “defect” as the absence or lack of something essential to completeness.^[^ ^29^, ^30^ ^]^ However, in the present scientific term, a defect is defined as the distortions of a periodic structure in a whole crystal.^[^ ^31^ ^]^ The crucial challenge in electrocatalysis includes recognizing and understanding the relations between the adsorbed states of intermediate reactions (i.e., microscopic levels) and the kinetic reactions (i.e., macroscopic properties).^[^ ^32^, ^33^ ^]^ For nanomaterials, defects are usually recognized as the active site(s) for the electrochemical process due to the tuned surface and electronic properties in the local region.^[^ ^33^, ^34^, ^35^, ^36^, ^37^ ^]^ Typically, the electronic states are enhanced at the topological region of defects, for example, when comparing the normal hexagon site(s) in the defect graphene (DG) with the combination of the carbon ring(s) with the “pentagon–octagon–pentagon”.^[^ ^38^, ^39^ ^]^ By combining the experimental studies with the theoretical calculations, the active sites for electrocatalytic reaction in FCs are convinced by the specific defects due to the particular desired binding energies of the reactants for each electrocatalytic reaction.^[^ ^40^ ^]^ Also, the electronic states or bandgaps near the Fermi levels of many 2D transition‐metal dichalcogenide can be altered via the crystal strains that the defect can induce at an edge or basal plane.^[^ ^41^ ^]^ Thus, defect engineering has been extensively employed to fabricate different nanomaterials to enhance the electrochemical reaction.^[^ ^33^, ^34^, ^35^, ^36^, ^37^ ^]^ However, to ensure the efficiency of this approach and gain a deep understanding of the design principle(s) for a specific electrocatalytic process, a controllable defect engineering method is paramount for adequately investigating the defects and the complementary roles on mechanisms of reactions. Moreso suggested that identifying the types of defects, for example, vacancies, interstitials, substitutions, dislocations, etc., and arranging them in order of contribution to specified different AOR and ORR will help advance and improve the electrocatalyst properties.

### 2.2 Defects Categories

Based on the dimensionalities, the defects of the material can be grouped into four classes, as shown in the table below, including 0D point defect (such as doping, vacancy, and reconstruction), 1D line defect (such as dislocation), 2D planar defect (such as the grain boundaries), and 3D volume defect (such as spatial disorder lattice). The details of these dimensionalities have been discussed in other studies.^[^ ^29^, ^42^ ^]^

## 3 Fabricating Strategies

The past few decades have witnessed the emergence of different synthetic strategies to construct defect‐based motifs in other electrocatalyst materials. These approaches can be mainly categorized into postfunctionalized and in situ synthetic methods. In the first approach, the ball‐milling method remains an effective and ecofriendly strategy to create and expose the more significant amounts of reconstructed and vacancy edge defect(s). For example, the fabrication of graphite nanosheets using a ball‐milling method has been demonstrated to produce a size‐reduced graphite nanosheet while significantly enhancing the electrocatalytic oxygen activation originating from the reconstruction of the oxygen defect motif on the zigzag edge.^[^ ^43^ ^]^

Plasma technology is another technique to form a uniform vacant defect structure(s) in carbon and transition metal oxides/dichalcogenides. This is because the plasma technology possesses good controllability, such as O_2_, Ar, or N_2_ plasma source types, processing time, and power density.^[^ ^44^, ^45^ ^]^ In addition, nonmetal‐atom and metal‐atom doping are other promising alternative techniques to obtain structural defects in carbon and transition metal oxides/dichalcogenides (such as CoSe_2_ and MoS_2_). This approach produces several dopant‐defect coordinated motifs that stabilize the structural topology of the defect(s) and manipulate the local electronic distributions by various coordinated configurations^[^ ^46^, ^47^ ^]^

High‐pressure hydrogenation is another technique analogous to the plasma method for treating the 2D iron–cobalt oxides by controlling the annealing temperatures and hydrogen pressure to turn the oxygen vacant density.^[^ ^48^ ^]^ On the other hand, acid etching of metal NPs anchoring at the defect sites is another widely used post‐treatment technique to obtain a metal defect motif at the atomic level.^[^ ^49^, ^50^ ^]^ Notably, it is crucial to consider the conditions of creating the defect engineering (including the defect density and type of defects) in a postfunctionalization method. These conditions are advantageous in generating the anticipated defect centers with optimum amounts and beneficial in selecting the most appropriate metal and nonmetal species as the guest dopants for modifying the defect.

However, the in situ synthetic method is a bottom‐up approach to tune the intrinsic defect density and types when creating defect‐based motifs in various materials. This method includes template‐based carbonizations,^[^ ^51^ ^]^ dopant removal annealing,^[^ ^40^ ^]^ pyrolyzes of Zn‐contained MOFs,^[^ ^52^, ^53^ ^]^ and electrocatalytic activation.^[^ ^54^ ^]^ For example, the MgO‐templated carbonization of C and N precursors has been used to prepare the series of N‐doped graphene meshes with different abundant topological defects.^[^ ^50^ ^]^ Different topological defect types were introduced on the graphene lattices by intentionally removing doped heteroatoms at a higher temperature. This was followed by the “aberration‐corrected transmission electron microscopy”, which directly confirmed the presence of diverse defects, including pentagon, heptagon, and octagon, on the edge of a DG sheet at the atomic scale.^[^ ^40^ ^]^ In addition, the Zn‐enriched MOFs denoted as the IRMOF‐8 were carbonized to obtain a high porous defect C owing to the complete removal of Zn at 950 °C.^[^ ^52^ ^]^ Upon the evaporation of the Zn‐induced defect situations, doping Zn cation in the other metal‐containing MOFs, such as ZnCo‐MOF, is known to exert the “fence function” to ensure a spatial expansion of the adjacent distance of the Co atom. Therefore, after a pyrolytic process, the induced vacant defects resulting from the evaporation of the Zn node engineer in situ preferential coordination with the Co node, forming the stable defect motif denoted as Co–N–C.^[^ ^53^ ^]^ Also, the electrocatalytic activation could create the defect site(s) in the C‐based nanomaterials, recalling that partial amorphous C can be oxidized and eliminated. The wet chemistry procedure can simultaneously generate an atomic metal fixed by the C–N surrounding defect sites.^[^ ^54^ ^]^

The overall aim of having a good defect‐inducing fabricating strategy is pivotal in achieving materials with excellent electrochemical activity. Carbon nanotubes (CNTs), carbon nanofibers (CNFs), carbon black, activated carbon, graphitic carbon nitride (g‐C_3_N_4_), graphene Oxide, etc. are tunable to have carbon vacancies by etching, dislocations, edge, and grain boundaries. These defects enhance electronic behavior, surface reactivity, high surface area, nanomaterials anchoring power, and electrical conductivity, collectively enhancing electrocatalytic performances.^[^ ^55,55^ ^]^

## 4 Defect Sites for Regulating the Electronic Structures

**Figure 1:**
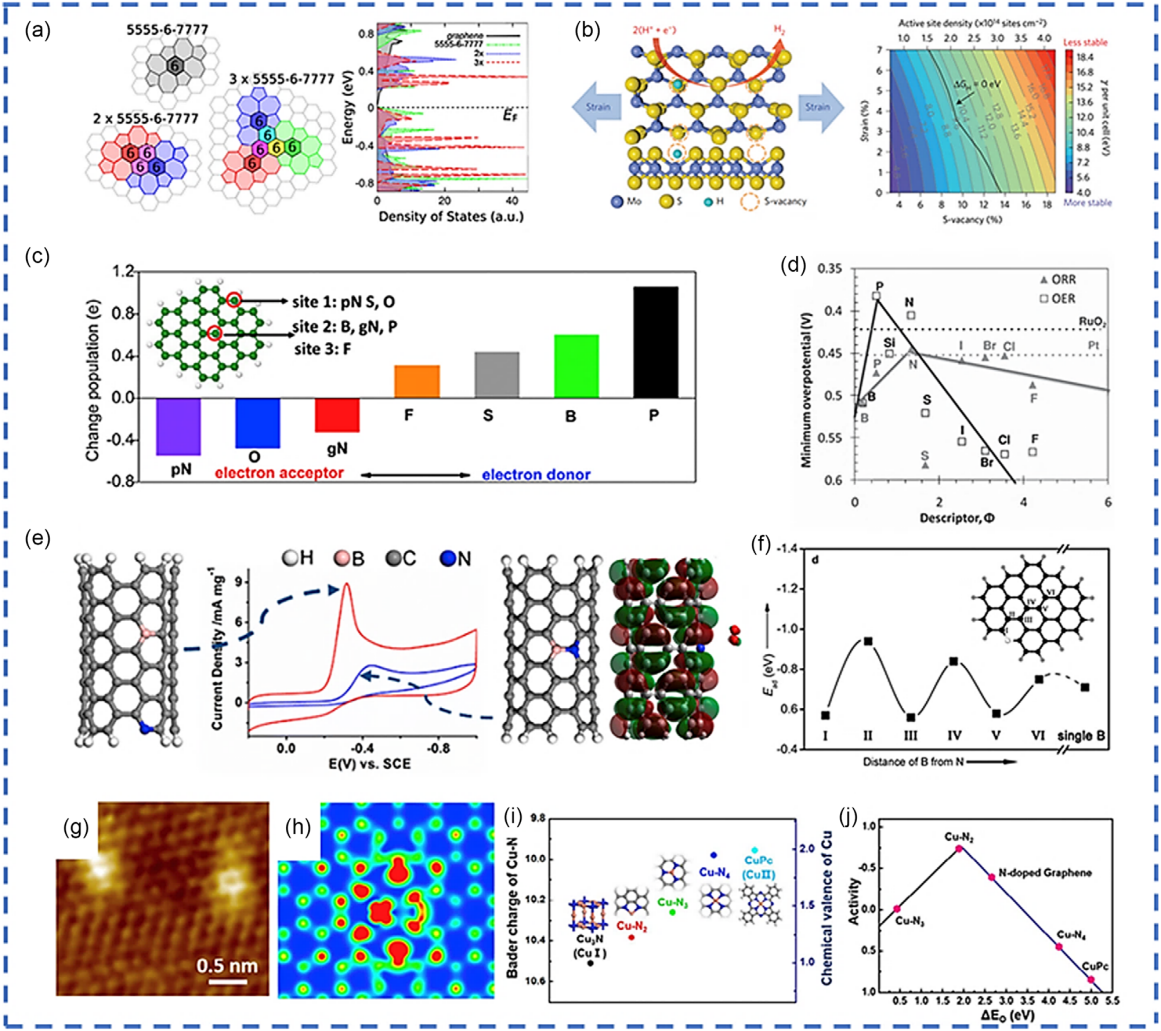
Various defect‐based motifs for electronic structure regulation. a) Three types of topological carbon defects and their electronic density of states. Reprinted with permission.^[^ ^36^ ^]^ Copyright 2019, American Physical Society). b) Schematic of the top (top) and side (bottom) views of MoS_2_ with strained S vacancies on the basal plane and the surface energy in relation to the densities of S vacancy and strain is shown in a colored contour plot. Reprinted with permission.^[^ ^59^ ^]^ Copyright 2019, Nature Publishing Group. c) Natural bond orbital (NBO) population analysis of six different nonmetallic heteroatoms in a graphene matrix. pN and gN represent pyridinic and graphitic N, respectively. Reprinted with permission.^[^ ^60^ ^]^ Copyright 2019, American Chemical Society. The inset shows the proposed doping sites for different elements. d) Volcano plots were obtained to correlate the ORR and OER overpotential and the intrinsic descriptor for doped graphene materials. Reprinted with permission.^[^ ^61^ ^]^ Copyright 2019, Wiley Online. e) Different coordination motifs of dual dopants (B and N) in CNTs show distinct ORR performances as a result of tuned electronic structures. Reprinted with permission.^[^ ^310^ ^]^ Copyright 2019, Springer. f) The C bridge plays an essential role among various B–C–N configurations with B active sites as a function of the distance to a pyridinic N atom (sites I–VI marked in the inset). Reprinted with permission.^[^ ^63^ ^]^ Copyright 2019, Nature Publishing Group. g–j) Atomic‐resolution STM image (g) and the corresponding simulation image (H) of single Cu atoms embedded in NG. Bader charge (left ordinate) and corresponding chemical valence of Cu atoms (right ordinate) for different structures Cu–N–(C) coordination motifs (i) as well as corresponding volcano plot of the relationship between ORR activity and Δ*E*_O_ (j). Reprinted with permission.^[^ ^63^ ^]^ Copyright 2019, Nature Publishing group.

It is essential to unravel the various electrocatalytic reactivity descriptor for a quick electrocatalyst screening for a given electrochemical reaction. The apparent rate of electrochemical reactions has been established to have a strong relationship with their intrinsic electronic structures (such as the work function, d‐band positions for the metal compound, and valence orbital levels for the nonmetals) of the electrocatalysts. This functions as an influential of intermediate adsorption energy, reaction barrier energy, and activation energy in the specific electrocatalytic reactions.^[^ ^13^, ^42^, ^56^, ^57^, ^58^ ^]^ For example, the experimental data and theoretical calculations have demonstrated that the point defect topology in the graphene basal plane can result in the bandgap opening or generation of abundant electronic state(s) near the Fermi level when compared with graphene defect (i.e., π and π* bands near the Fermi levels are doubly generated), as presented in **Figure** **1**a.^[^ ^34^, ^36^, ^40^ ^]^ Similar to the C materials in defective compounds, for example, MoS_2_ with S vacancy, the band structures can be tuned stepwise by changing the S vacant concentrations, thereby reducing the hydrogen adsorption free energy (Δ*G*_H_) (Figure 1b).^[^ ^59^ ^]^

Regarding the nonmetallic dopant‐induced defect motif(s), with S, F, P, and B functioning as the donor of electrons and O and N serving as the electron acceptors for an adjacent carbon atom, six nonmetallic heteroatom materials that have several electronegativities have been selected to examine their influence on the adjacent carbon which functions as the active site for HER.^[^ ^60^ ^]^ It was proposed that the C‐defect‐based motif could modify the local population charges in graphene matrices through multiple dopant coordination, as presented in Figure 1c.^[^ ^60^ ^]^ From our perceptive, the realized properties of the HER materials used can be borrowed for application in FC materials that will utilize edge catalysis, promotional effect, and hybridization‐induced synergy for enhanced FC electrocatalytic activity and thus ultimate excellent surface/interface properties.

In addition, a new descriptor activity has been proposed based on the synergistic effects of electron affinity, and the electronegativity on the redistribution of charges has been reported for a correlative study on the inherent electrocatalyst features in electrocatalytic performance: Φ = (*E*_X_/*E*_C_) × (*A*_X_/*A*_C_).^[^ ^61^ ^]^

The researchers have discovered that modulated nonmetallic heteroatom carbon defect‐based motifs might also significantly impact electron transport and reaction energy in the energy conversion (e.g., ORR, oxygen evolution reaction (OER), etc.) with certain electronic topologies (Figure 1d). On the other hand, the position of several “heteroatom dopants” in the carbon matrices is critical. Zhao et al. reported that “two types of B‐ and N*‐*codoped CNTs dominated by bonded or separated N and B were purposefully generated by chemical vapor deposition growth or after treatment, with unique ORR performances (Figure 1e).”^[^ ^62^ ^]^ The inertness of the bonded B‐ and N*‐*codoped CNTs can be attributed to the fact that the lone pair electrons from the N dopant are mainly neutralized by the unoccupied orbital of the B dopant, and only just a few electrons from the N dopant or the carbon system are allowed to conjugate with the unoccupied (vacant) orbitals.^[^ ^62^ ^]^ According to Zheng et al., the C bridge sites have been observed to play synergetic roles among various B–C–N configurations in the graphene matrices, with the B active site as a function of the distance to a pyridinic N atom. The strength of the synergistic effect gradually decreases as the distance between the B and pyridinic N dopants increases (Figure 1f).^[^ ^63^ ^]^ Besides nonmetal dopants, a defect on the support can also function as the “docking” sites for capturing the atom metal species to develop unique metal–defect coordination motifs to boost electrocatalytic performance. Vacancies on the supporting matrix, in particular, can capture a variety of metal species at the atomic scale based on geometric defect patterns. They can also carefully adjust the electrical structures of the central metal atoms via various surrounding configurations. By pyrolyzing the Cu dicyandiamide and phthalocyanine, Wu et al. created atomically dispersed Cu–N–C coordination motifs with high density in an NG matrix.^[^ ^64^ ^]^ The scanning tunneling microscope (STM) images showed that an embedded Cu atom rendered the neighboring N and C atoms electronically rich, as presented in Figure 1g,h.

The “variable chemical valence states” of the “Cu atom coordinated with different numbers of N atom in different Cu–N–(C) coordination motifs” (Figure 1i). The corresponding volcano plot of the relationship between ORR activity and EO indicates that Cu–N_2_ is the best Cu atom‐modulated NC defect motif for ORR (Figure 1j). In summary, “vacancy, nonmetallic, and metallic dopants” can artificially modify the electronic structures of defect sites to reach the best “binding energy” of various adsorption intermediate levels for specific electrocatalytic processes.

## 5 Characterizations of Defects

Because of the inherent complexities of the defects, increasingly sophisticated characterization devices have been introduced to meet the technological demands for investigating them. Different methods can be used to analyze the influence and stances of defects in electrocatalysts, including “X‐ray diffraction” or “scattering patterns”,^[^ ^65^ ^]^ “Raman spectroscopy”,^[^ ^66^ ^]^ “XPS”,^[^ ^67^ ^]^ “XAS”,^[^ ^49^ ^]^ “aberration‐corrected transmission electron microscopy (ACTEM)”, and^[^ ^68^ ^]^ “positron Anni (HAADF–STEM).”^[^ ^69^ ^]^ By comparison, only ACTEM and HAADF–STEM allow direct characterization of surface flaws in electrocatalyst morphologies among these techniques.

**Figure 2:**
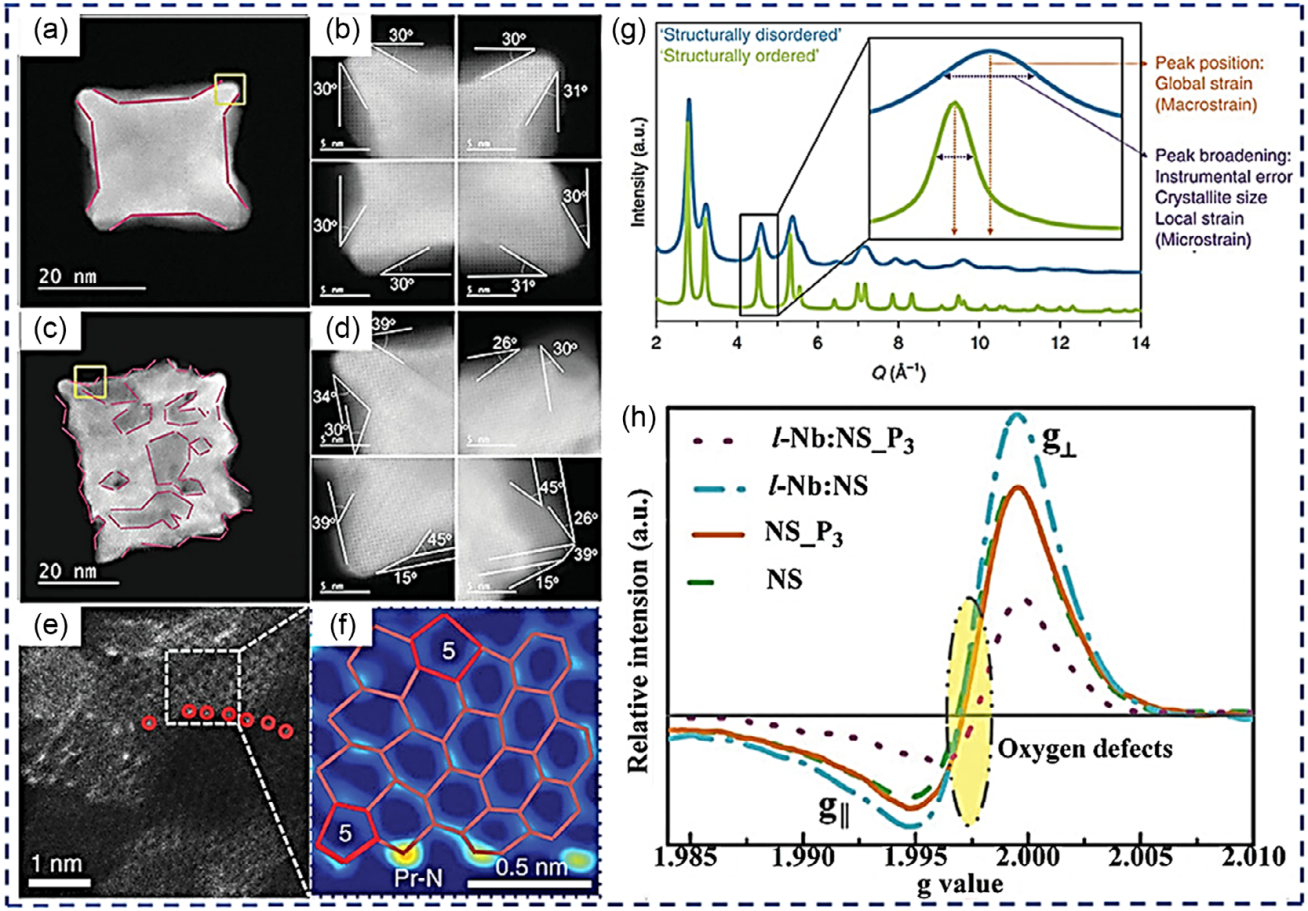
a,c) “HAADF–STEM images and b,d) enlarged images of the four corners of concave cubic and defect‐rich cubic nanocrystals. Reproduced with permission.^[^ ^70^ ^]^ Copyright 2016, Wiley–VCH e) HAADF–STEM images of N‐doped graphene. The red circles represent N atoms, and f) an expanded image of the dotted box in (e) (“5” indicates a pentagon defect). Reproduced with permission.^[^ ^71^ ^]^ Copyright 2019, Nature g) Typical WAXS patterns for electrocatalysts. Reproduced with permission.^[^ ^65^ ^]^ Copyright 2018, Nature h) EPR spectra of selected nanomaterials”. Reproduced with permission.^[^ ^72^ ^]^ Copyright 2018, Nature.

By comparing the morphology of cubic concave nanocrystals with and without defects, the disordered alloy electrocatalysts' defect structure(s) might be studied at the subnanometer and even the atomic scale. This is because the darker portions of the faulty electrocatalysts correspond to the depressions, whereas the brighter area corresponds to a thicker atomic layer (**Figure** **2**a,b). As presented in Figure 2c,d, the angles at the four corners of the nanocrystals were measured, and the interfacial angle(s) decreased from “defect‐rich cubic” to “concave cubic” Pt_3_Sn, indicating that defective catalysts contain more edge sites.^[^ ^70^ ^]^ ACTEM images can also be used to evaluate intrinsic defects in non‐PGM catalysts (carbon pentagon) (Figure 2e,f).^[^ ^71^ ^]^

Furthermore, because the X‐ray diffraction (XRD) patterns break the original crystalline structures and increase/decrease crystallite dimension in nanomaterial, they may be utilized to identify defects. The “simultaneous shifting and width broadenings” of the peaks revealed structural disorder in a PtNi alloy (Figure 2g).^[^ ^65^ ^]^ Electron paramagnetic resonance (EPR) spectroscopy remains a straightforward way to measure the unpaired electrons of electrocatalysts because of the varied coordination‐unsaturated structural defects. A rise in the strength of EPR peak(s) indicates an increasing oxygen vacancy concentration, as shown in Figure 2h.^[^ ^72^ ^]^ Currently, certain intricate defects are nevertheless impossible to precisely and thoroughly validate. A combination of in situ and surface characterization should be used to comprehend and appreciate the different types of defects fully.

The characterization of defects is significant in enhancing AOR. By knowing the exact type of defect, researchers will have a better understanding of reaction kinetics toward AOR as it aids in understanding the active sites and kind of interaction of NPs with support materials. With that, the structure–property relationship can be mapped well, thereby promoting the synthesis of electrocatalysts with optimized defect densities and types. We believe that different defects aid/point to a specific reaction mechanism; thus, the most efficient electrocatalysts with unique electrocatalytic activity and selectivity are possible.

## 6 Relationship between Electrocatalytic Abilities and Defect Engineering

Designing and optimizing defects in an electrocatalyst require a proper understanding of the link between engineering and catalytic abilities. Previous reports have shown that defects might accurately alter the “Fermi level of semiconductor oxides” to vary the adsorption energy of the reactive intermediate species.^[^ ^73^ ^]^ Nevertheless, as defective catalysis progresses based on theories and methodologies, more emphasis has been placed on “defect engineering” to control electrocatalysts’ surface/interfacial characteristics and electronic configuration.^[^ ^74^ ^]^ Adsorption energy (which refers to one or more adsorption/desorption species) and the reaction pathway of a certain catalytic process are linked to the reaction rate.^[^ ^75^, ^76^, ^77^ ^]^ The adsorption/desorption strength of a reactant, intermediate, and/or product can significantly impact the reaction rates and the rate‐determining steps of an electrocatalytic process.^[^ ^78^, ^79^ ^]^ The increase of OH adsorption species or inhibition of OOH adsorption species slows down the reaction rate during ORR.^[^ ^80^ ^]^ For instance, it has been discovered that a pentagonal carbon defect near the zigzag's edge could control the local electronic distribution of surrounding carbon atom(s), thereby allowing for more significant OOH adsorption and ORR electrocatalysis.^[^ ^38^ ^]^ In addition to the ORR, defect engineering may accurately adjust the adsorption energies in additional electrocatalytic process (HER, OER, etc.).

**Figure 3:**
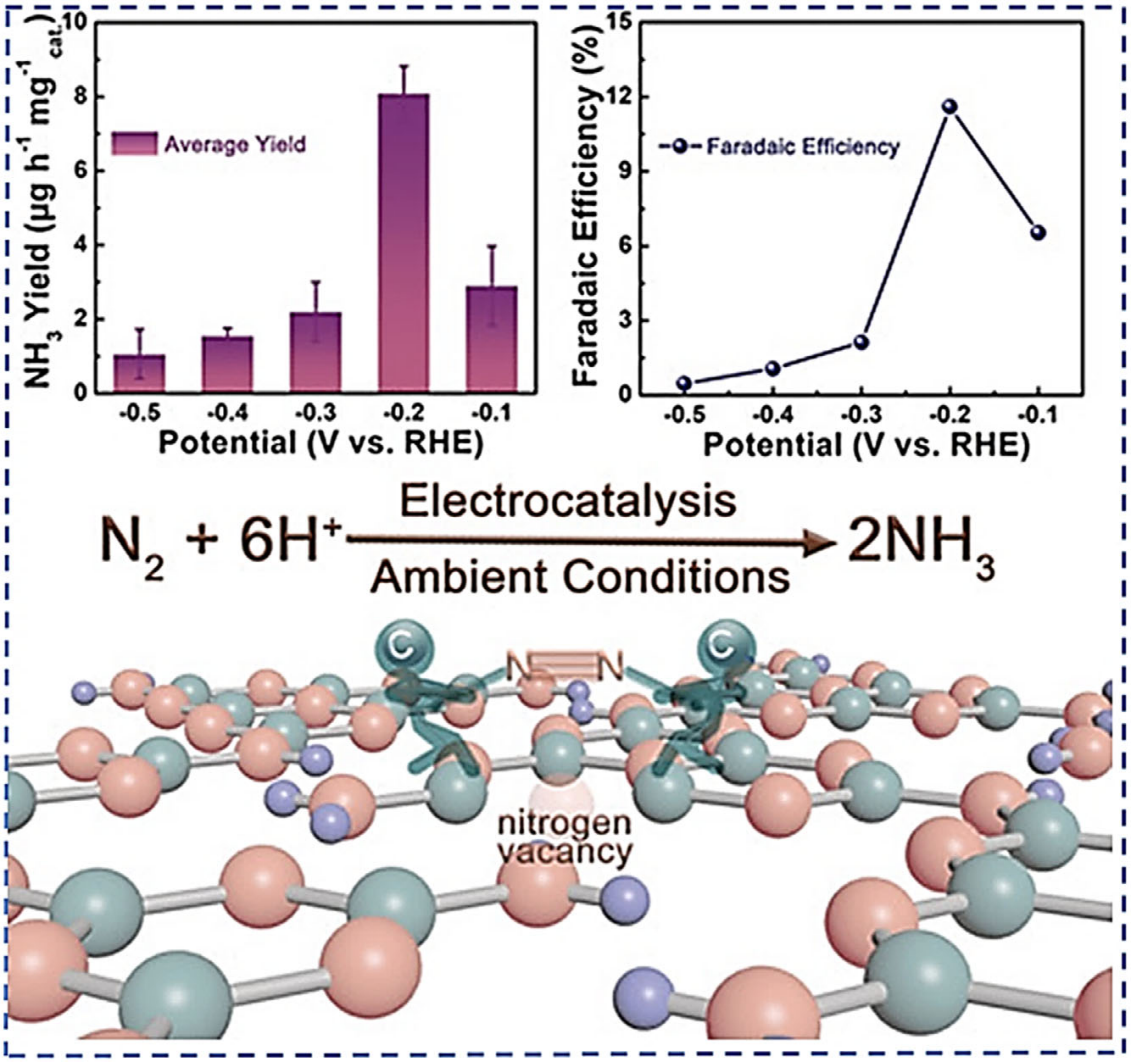
Defect engineering strategy for the electrocatalytic synthesis of ammonia. Reproduced with permission.^[^ ^85^ ^]^ Copyright 2018, Wiley–VCH.

The use of highly oriented pyrolytic graphite as a model electrocatalyst to develop the structure–property link has been reported to demonstrate the intrinsic defect in defective carbons, thereby modifying the “surface charge” of the active site to govern the adsorption energy of reactive intermediate.^[^ ^81^, ^82^, ^83^ ^]^ Similarly, it has been demonstrated that doped F defects in carbon activated the neighboring carbon atom as an electrocatalytic site by enhancing the COOH adsorption while reducing H adsorption. This results in improved efficiency.^[^ ^84^ ^]^ N vacancies in carbon nitride electrocatalysts have been discovered to aid in the N_2_ chemisorption, extend its N≡N bond, and change the electrocatalyst's structural and electronic properties, thereby making it more electroactive (**Figure** **3**).^[^ ^85^ ^]^ Understanding the defects through creating linkages between defect engineering and catalytic abilities by managing the surface/interfacial characteristics, electrical structure, reaction route, and intermediate species of the adsorption energy are crucial.

For well‐engineered defective electrocatalysts in DAFCs, the defect engineering protocols ensure the modifications, concentration, and distribution of the defects (morphological defects, e.g., cracks, voids, or uneven surfaces), surface defects (steps, terraces), etc. Thus, inducing a specific defect into a material helps create active sites and highly selective electrocatalysts. When there is an introduction of a defect in a material, there will be a complimentary alteration of the electronic structure, which is mainly seen in supported Pt or Pd NPs XPS, where there will be a shift in the binding energies representing a change in the d band center.^[^ ^86^, ^87^ ^]^ For example, the edge of zigzag on carbons can regulate the local electronic distributions,^[^ ^38^ ^]^ and intrinsic defects in defective carbon adjust the surface charge of active sites to control.^[^ ^88^, ^89^ ^]^ Thus, the electronic structure medication enhances electrocatalytic performances, for example, enhanced reaction kinetics, excellent durability, and reduced energy losses during electrochemical reactions (lower overpotentials). The optimization of the defects is of greater importance, which we postulate to be achievable via a deep defect analysis and contribution by iteratively integrating morphology and electrocatalysts with electrochemical composition. Therefore, using diverse materials helps create heterointerfaces or hybrid electrocatalyst structures that synergistically boost electrocatalytic capabilities and utilize defect‐related constraints. However, correctly identifying and comprehending defects in the catalysts is currently challenging. As a result, future research on defective catalysts should concentrate on the mechanistic aspects of the electrocatalytic processes through experimental and theoretical simulations.

## 7 Electrocatalyst Classification

### 7.1 Nonmetal Based

Mostly the nonmetal‐based electrocatalyst has a very low activity that renders practical application for AOR. The reasons being the lack of redox (redox probes) activity, weaker alcohol adsorption that limits the effective reactant–electrocatalyst interactions (which results in inert surface interactions), and limited‐to‐no alcohol‐active sites. Thus, mostly the nonmetal‐based electrocatalysts are more active for ORR. Metalloids have, over the years, been utilized as dopants on carbon materials since they significantly increase the oxygen reduction activity of carbon‐containing catalysts. In the quest to find the best metalloid dopant, germanium (Ge) has attracted much attention because of its special properties, such as being a good semiconductor, having high carrier mobility, and 10 000 times higher conductivity when compared to Si, which is another metalloid in contention. Ge can also be doped easily into carbon materials; hence, it is an interesting choice as a metalloid dopant. The low electronegativity of Ge compared to that of carbon makes it an ideal dopant since it can modify the electronic structure of the carbon matrix and impart its electronegative properties.^[^ ^90^ ^]^

Chang et al. investigated an N‐ and P‐codoped system of Ge. They revealed that codoping with nitrogen and sulfur effectively reduced the ORR intermediates' work function and increased the Ge–NP system's ORR activity. As in **Table** **1**, the electrocatalytic analysis in 0.1 m KOH electrolyte solution of the synthesized Ge–N–P–rGO, Ge–N–rGO, and Ge–P–rGO showed that Ge–N–P–rGO had a superior half‐wave and onset potential when compared with the conventional Pt/C and its counterparts. The Ge–N–P–rGO was reported to perform ORR in a four‐step electron process.^[^ ^91^ ^]^

**Table 1:** Summary of defects and their specific examples

Dimension	Defect name	Example
0D	Point	Doping, vacancy, reconstruction
1D	Line	Dislocation
2D	Planar	The grain boundaries
Three‐dimension (2D)	Volume	Spatial disorder lattice

The catalyst becomes more active as the onset (more negative) and half‐wave potential become more positive. As shown in Table 1, Ge–N–P–rGO has a more positive onset and half‐wave potential, indicating that this catalyst is more involved than Pt/C. The analogous criterion's onset and half‐wave potential for the Pt/C were 0.92 and 0.83 V, respectively. Ge–N–P–rGO showed superior stability toward ORR according to the chronoamperometric measurements done at 0.4 versus reversible hydrogen electrode (RHE) as the current density of Ge–N–P–rGO exhibited only a 16% decrease after 10 h reaction, whereas Pt/C was reduced by 26% from its initial current as shown in Table 1. Ge–N–P–rGO also has a comparable limiting current density to the Pt/C, as shown in Table 1. This value is constant for a four‐electron transfer of ORR at 1600 rpm over the working electrode with dia. 5 mm for a given concentration of electrolyte solution, 5.968 mA cm^−2^ for 0.1 m KOH solution.^[^ ^91^ ^]^ The electron transfer number (*n*) derived from Koutecky–Levich (K–L) plots for Ge–N–P–rGO is as high as 4.2 at 0.5 versus RHE, enhancing ORR selectivity.

Doping heteroatoms such as N, S, O, B, Fe, Co, and/or Mn to carbon materials such as graphene, CNTs, graphitic arrays, and amorphous carbon is known to contribute massively to the overall electrocatalytic performance of these materials toward ORR. Even though introducing heteroatoms to carbon‐supporting materials is only limited to surface modification, this method has established credibility since it results in refined electronic properties and increased stability. Carbon‐based metal‐free electrocatalysts usually have less ORR activity in acidic electrolytes than in alkaline electrolytes. It remains a challenge to make them efficient on polymer‐exchange membrane FCs (PEMFCs).

In contrast to what has been mentioned above, poly [cyclotriphosphazene*‐co*‐1,3,5‐triol nitrobenzene] (PCTNB) microspheres with uniform size and diameter of more than 2 μm were synthesized and incorporated into CNTs by a template‐based noncovalent method by Ullah Dar et al., to form a C‐PCTNB@CNTs composite. No significant corrosion under an acidic medium is observed on the carbon electrode when this composite is utilized as an electrocatalyst since carbon is more anticorrosive to acids than transition metals containing electrocatalysts.

ORR polarization curves of N‐, P‐, O‐doped (C‐PCTNB@CNTs) and commercial Pt/C catalysts in O_2_‐saturated 0.1 m HClO_4_ solution showed higher onset potential for C‐PCTNB@CNTs as shown in Table 1 with low‐half‐wave potential which is normal for carbon‐based materials. The kinetic and mechanistic performance of the C‐PCTNB@CNTs catalyst was also observed using Tafel plots. From the plot, the catalyst had a Tafel slope of 78 mV per decade comparable to that of the Pt/C, which was 68 mV per decade. The Tafel slopes were also under 120 mV, which showed no mass transfer issues conflated with the kinetics. The durability of the C‐PCTNB@CNTs catalyst was also investigated, and it was observed that the degradation in the half‐wave potential of ORR polarization curves for the N‐, O‐, and S‐doped composite was only 15 mV after 10 000 potential cycles, which stands out from that of the conventional Pt/C which is almost 40 mV.^[^ ^92^ ^]^ C‐PCTNB@CNTs catalyst is also very stable under ORR conditions as they show negligible activity decay over time in terms of the current density, which is not the case for Pt/C. The electron transfer number (n) was determined to be 3.9 over 0.4–0.7 V from Koutecky−Levich plots. This shows that the C‐PCTNB@CNTs catalyst kinetic route produces minimum H_2_O_2_.

Pyrolysis of porous MOF has emerged as a novel approach to synthesizing carbon materials for electrochemical energy applications. The advantage of producing carbon materials as catalysts for ORR via this route is that these materials possess an atom‐level control of composition and uniform atom‐level distribution in heteroatom‐doped carbon materials due to well‐defined MOF precursors and organic linkers. Another noticeable advantage is that these carbon materials have a high specific surface area and hierarchal mesoporous structures due to uniformly distributed pore‐forming agents (MOFs) and tunable morphology. The electrochemical analysis of NHMC‐900, a resulting carbon material synthesized by pyrolysis of interpenetrated MOF at 900 °C, reveals superior onset and half‐wave potential compared with the Pt/C with a standout onset potential that was 1.0 V, as shown in Table 1. The synthesized carbon catalyst is also insensitive to anion adsorption like phosphates and sulfates compared to the Pt/C since its onset potential remained roughly at 0.88 V versus. RHE in three acidic electrolytes (0.1 m HClO_4_, 0.05 m H_2_SO_4_, 1.0 m H_3_PO_4_).^[^ ^93^ ^]^ The electron transfer number (n) in O_2_‐saturated 0.1 m KOH solution for NHMC‐900 was close to four (3.79–3.99) in the 0.2–0.9 V range, suggesting a four‐electron pathway.

### 7.2 Metal‐Based

#### 7.2.1 PGM‐Based

In spite of the significant advantages of Pt–RE alloy catalysts, their greatest hindrance comes from the synthesis point of view due to the low reduction potential of the lanthanides compared to that of the Pt, which is typically less than −1.9 V versus normal hydrogen electrode. Another hindering factor is that the energy gained by rare earth metals after alloying with Pt is around 4 eV, which is significantly lower than their counterparts since oxides are around 10 eV. This causes the RE metals to form oxides easily when in contact with any oxidizing agents during the synthesis process, which is undesirable. Therefore, this poses a challenge in reducing the RE metals to their metallic state by traditional coreduction methods. To date, physical metallurgical methods are the most successful methods documented in the literature for synthesizing Pt‐RE alloy catalysts, resulting in a 100% alloy conversion rate.^[^ ^94^ ^]^

Some researchers have attempted to synthesize Pt–RE alloy catalysts by chemical reduction methods where the RE component exists in an oxide or hydroxide state. The strain‐induced compression effect from alloying Pt in its metallic phase with RE in an oxide state results in a Pt/oxide junction with microstructural defects operating at the junction, resulting in a catalyst with high ORR performance and stability. Sandström et al. synthesized a Pt_*x*_YO_*y*_/C alloy catalyst utilizing microwave irradiation. The precursor metal salts were first mixed with tetrahydrofuran and then dried. The resulting powder was then subjected to microwave irradiation under the flush of 5% H_2_ in an Ar mixture. A microwave of 700 W power was used, and the mixture was subjected to microwave irradiation for 150 s to form a yellow‐orange powder of Pt_3_YO_*y*_/C nanoparticles (NPs).^[^ ^95^ ^]^

The durability test for the Pt_3_YO_*y*_/C NPs was measured using linear sweep voltammetry (LSV), which measured accelerated degradation of the Pt_3_YO_*y*_/C electrode by cycling the potential between 0.6 and 1.0 V versus RHE in an oxygen‐saturated acidic medium over 6000 times. Those results indicated that the mass activity of that catalyst after 6000 cycles of Pt_3_YO_*y*_/C was still higher than that of the commercial Pt/C, which indicates the superior appeal toward the ORR process for this catalyst over the reference catalyst. The Pt_3_YO_*y*_/C catalysts were also evaluated under real‐life FC environments, and they exhibited a potential of 0.54 V at 1 A cm^−2^ which compares with that of the conventional Pt/C, which is 0.65 V.^[^ ^95^ ^]^ The electron transfer number derived from K–L plots for the Pt_3_YO_*y*_/C catalyst was similar to that of the Pt/C (*n* = 4) despite indications of modified Pt sites on the Pt_*x*_YO_*y*_/C samples with the introduction of yttrium. The above results show that the Pt_*x*_YO_*y*_/C catalyst supports a full four‐reaction pathway with minimal H_2._ The specific activity of the Pt_3_YO_*y*_/C catalyst is shown in Table 3 as that of the Pt/C (0.17 mA cm^−2^ Pt). The mass activity of the catalyst was also larger than that of the reference catalyst (0.14 Amg_Pt_^−1^). These results elucidate that the Pt_*x*_YO_*y*_/C catalyst has superior ORR activity in an acidic media than the Pt/C. The higher mass activity on Pt_*x*_YO_*y*_/C is higher even though the catalyst has a larger average NP size and lower ECSA, which shows that the catalyst's surface greatly benefits from incorporating yttrium.^[^ ^95^ ^]^

Platinum catalysts have attracted significant attention and are used on an FC's anode and cathode parts due to their high electrocatalytic activity. Still, their scarcity and easy poisoning at the Pt surfaces seriously confine their further practical applications. To mitigate this issue, alloying Pt with noble‐based metals like Cu provides a feasible approach to forming Pt‐based nanocrystals with high Pt utilization efficiency and cost‐effectiveness. This approach also vastly improves the Pt catalytic characters.^[^ ^96^ ^]^

Huang et al. fabricated the design of 3D bimetallic PtCu alloyed nanocages (PtCu NCs) via a one‐pot solvothermal method. The PtCu NCs catalysts showed enhanced electrocatalytic performance toward glycerol oxidation and ethylene glycol oxidation reactions in alkaline media compared to the Pt/C and Pt on carbon black. As shown in Table 3, the onset (1.02 V) and half‐wave (0.95 V) potential of the PtCu NCs were the most positive in reference to the Pt/C (0.97, 0.90 V, respectively) and Pt on carbon black, which demonstrates excellent ORR kinetics at a relatively low overpotential in 0.1 M KOH solution. The mass and specific activity of the PtCu NCs catalysts were 2.1 and 5.1 times higher with respect to those of the Pt/C and 7.6, and 5.9‐fold higher relative to those of Pt black, which confirm the remarkable improvements of the bimetallic catalyst toward ORR activity. The mass and specific activity provided for PtCu NCs (shown in Table 3) in an alkaline media with 0.5 m methylene glycol are 2.1 and 5.1 greater than the mass and specific activity of the Pt/C (1.29 A mg^−1^, 2.65 mA cm^−2^ respectively). The above results show improved methylene glycol oxidation for PtCu NCs, which is superior to Pt/C. For glycerol oxidation, the PtCu NCs show a mass and specific activity shown in **Table** **2** that are 2.0 and 3.8 times larger than the Pt/C (1.29 A mg^−1^, 2.65 mA cm^−2^), which elucidate superior glycerol oxidation activity for the bimetallic catalyst. The ratio of the *j*_f_/*j*_b_ for the bimetallic catalyst is 3.4, which is larger than that of the Pt/C (3.0), and this signifies the enhanced antipoisoning ability of PtCu NCs when ethylene glycol and glycerol are used as alcohols for oxidation reactions.^[^ ^96^ ^]^

**Table 2:** Non‐metal‐based electrocatalysts synthesized using various synthetic routes and their ORR protocols.^[^ ^91^, ^318^, ^319^ ^]^

Catalyst	Catalyst synthesis route	Electrolyte	1) ORR *E*_on_ a)/*E*_1/2_ b) V versus RHE. 2) Mass activity/specific activity	Current Density	Electron transfer number [*n*]	Tafel slope	Scan rate
Ge–N–P–rGO	Hydrothermal process	0.1 m KOH	1) 0.94 V (onset) 0.84 V (half wave)	−5.1 mA cm ^−2^	4.2	–	Not reported
C‐PCTNB@CNTs	Facile template‐based noncovalent method	0.1 m HClO_4_	1) 0.94 V versus RHE (onset) 0.85 V (half)	−6.3 mA cm ^−2^	3.9	78 mV dec^−1^	50 mV s^−1^
NHMC‐900	Pyrolysis of the nonporous‐interpenetrated MOF precursor	0.1 m KOH	1) 1.0 V versus RHE (onset) 0.88 V (half)	–	3.99 (high potential) 3.79 (low potential)	–	5 mV s^−1^

a) Onset potential.

b) half‐wave potential.

Platinum‐based ternary display dramatically enhanced electrocatalytic performance compared to their mono‐ and bicounterparts owing to the synergistic effect of the trimetals and enhanced efficient utilization of the Pt metal on the trimetallic system. Han et al. proposed a one‐pot solvothermal method to synthesize multicomponent and uniform 3D PtCoRh ternary catalysts with highly branched nanoassemblies. The prepared ternary catalyst showed outstanding electrocatalytic performance toward ethanol and methanol oxidation reactions (MORs) in 1 m KOH electrolyte solution since the mass and specific activities of this catalyst for EOR and MOR were remarkably higher in comparison to those of Pt/C, as shown in **Table** **3**. Cyclic voltammetry (CV) curves in an electrolyte solution of 1 M KOH with 1 m ethanol for the PtCoRh catalyst showed a lower onset potential of 0.25 V, which was lower than that of Pt/C, which was 0.56 V. The above results depict distinctive improvement in the EOR kinetics on the ternary PtCoRh catalyst over the commercial Pt/C. The peak current density for the PtCoRh catalyst over a half‐wave potential of 0.61 V is 65.67 mA cm^−2^, which is remarkably higher than that of the Pt/C (5.62 mA cm^−2^), implicating that the trimetallic catalyst has superior EOR activity. The mass and specific activity of PtCoRh in an alkaline solution with 1 m ethanol were 1.75 A mg^−1^ and 4.03 mA cm^−2,^ respectively, larger than those of Pt/C (0.17 A mg^−1^ and 0.75 mA cm^−2^ ), and these results demonstrate the efficiency of the trimetallic catalyst. Chronoamperometry was utilized to measure the durability of the trimetallic catalyst at a half‐wave potential of 0.61 V on an alkaline solution with 1 m ethanol, and the catalytic current density for the trimetallic catalyst was higher than that of the monocatalyst after 4000s, which indicates the remarkable stability of PtCoRh over Pt/C. Further electrochemical measurements were done for the trimetallic catalyst under an alkaline solution with 1 m methanol, and the PtCoRh catalyst still showed superior catalytic activity, as depicted in Table 3.^[^ ^97^ ^]^

**Table 3:** Pt‐RE alloy electrocatalysts synthesized using various synthetic routes and their ORR and alcohol oxidation protocols.^[^ ^95^, ^96^, ^97^ ^]^

Catalyst	Catalyst synthesis route	The fate of the RE component or metal component (oxide/alloy)	Electrolyte	1) ORR onset potential/half‐wave potential V versus RHE 2) Mass activity/specific activity 3) Current density	Scan rate	Fuel cell performance evaluation parameters
Pt_*X*_YO_*y*_	Dry‐state microwave irradiation procedure	Oxide	0.1 m HClO_4_	2) 0.32 mAcm_Pt_^−2^ (specific) 0.20 mAg_Pt_^−1^ (mass)	50 mV s^−1^	Cell potential of 0.54 V For Pt_3_YO_*y*_/C, and 0.65 V for Pt/C reference at 1 A cm^−2^. Catalyst loading: 0.2 mg Pt ^−1^, 5 cm^−2^ active area, 60 °C, H_2_–O_2_
PtCu NCs	One‐pot solvothermal method	Alloy	0.5 m KOH + 0.5 m EG/glycerol (alcohol oxidation). 0.1 m KOH (oxidation–reduction)	1) 1.02 V (onset) 0.95 V (half wave), ORR 2) 1.28 A mg^−1^ (mass), ORR 6.46 mA cm^−2^ (specific), ORR 2) 2.65 A mg^−1^ (mass), GOR 13.40 mA cm^−2^ (specific), GOR 2)) 1.28 A mg^−1^ (mass), EGOR 6.46 mA cm^−2^ (specific), EGOR	50 mV s^−1^	Not specified
PtCoRh	One‐pot solvothermal method	Alloy	1 m KOH + 1 m ethanol (EOR) 1 m KOH + 1 m methanol (MOR)	1) 0.26 V (onset) 0.61 V (half), EOR 0.74 V (half), 0.37 V onset (onset) 2) 1.75 A mg^−1^/4.03 mA cm^−2^ (EOR) 0.98 A mg^−1^/2.34 mA cm^−2^ (MOR) 3) 65.67 mA cm^−2^ (current density)	50 mV s^−1^	Not reported

#### 7.2.2 Non‐PGM‐Based

The non‐PGM‐based electrocatalysts are a bad candidate for AOR. They have low reaction kinetics and electrocatalytic stability and are easily poisoned when used as standalone electrocatalysts. In contrast, the non‐PGM‐based electrocatalysts are active toward ORR, as this section will point out. Due to their prominent conductivity and stability, transition metal carbides (MXenes) possessing hydrophilic surfaces have attracted increasing attention as promising electrocatalysts. Notably, employing hydrophilic MXenes as electrocatalysts for ORR offers the following advantages: 1) highly efficient charge carrier transfer due to the prominent metallic conductivity of 2D MXenes; 2) stronger redox reactivity than that of other carbon‐based materials; 3) high stability in aqueous media; and 4) strong interaction with catalytic targets as a result of the hydrophilic moiety in their structure. Ti_3_C_2_ MXene‐based catalyst with desirable ORR activity and stability in alkaline media was fabricated through a liquid exfoliation process by combining HF etching (delamination) and TPAOH intercalation (disintegration) by Lin et al..^[^ ^12^ ^]^

SL Ti_3_C_2_ MXene‐based catalyst shows a 9 mV reduction in its half‐wave potential after 1000 cycles between 0.4 and 1.0 V versus RHE in 0.1 m KOH electrolyte solution while the conventional Pt/C shows a 25 mV reduction of its half‐wave potential which shows that the catalyst is stable at accelerated durability rates. Chronoamperometric measurements confirm that the MXene catalyst offers better stability than Pt/C. The Pt/C showed a higher current decay after continuous operation at 10 000 s as opposed to the SL Ti_3_C_2_ MXene catalyst, which retained 87% of its initial current, showing that it is more durable for the ORR process in alkaline media.^[^ ^98^ ^]^ Electrochemical impedance spectroscopy measurements from the Nyquist plot showed low charge transfer resistance (*R*_ct_) of SL Ti_3_C_2_ MXene catalyst, indicating this catalyst's highest conductivity, which facilitates the remarkable enhancement of ORR activity. The electron transfer number for SL Ti_3_C_2_ was around 3.7 at 0.55–0.70 V under K–L plots, indicating that the catalyst favors a four‐electron pathway of the oxygen reduction process, similar to the high‐quality commercial Pt/C catalyst. The Tafel slope of SL Ti_3_C_2_ was 64 mV per decade in 0.1 m KOH, which is lower than the Pt/C and suggests that the MXene catalyst features favorable ORR kinetics. The methanol crossover effect was also investigated for the SL Ti_3_C_2_ catalyst. The introduction of methanol on SL Ti_3_C_2_ on O_2_ saturated 0.1 m KOH solution resulted in minimal deterioration of the ORR performance of the SL Ti_3_C_2_ catalyst, which proffers the applicability of the catalyst on direct methanol fuel cells (DMFCs).

Heteroatom‐doped carbon materials show promising prospects for ORR activity, especially those with transition metal and nitrogen as dopants on the carbon material. According to the literature, (Fe‐N/C) carbon materials that are nitrogen doped and which have iron as the transition metal show outstanding durability and electrocatalytic performance. Due to their interesting features, further research and progress have been made toward Fe‐N/C materials to identify the active sites on such materials for ORR. Creating active sites for ORR, such as N_*x*_, Fe_3_‐C@C, and N/C moieties, are considered the most effective means of increasing the electrocatalytic performance of Fe‐N/C materials. Another crucial factor is that creating mesoporous structures of Fe‐N/C has an impact on increasing the number of active sites for ORR and exposing abundant active sites of ORR to electrolytes. Interconnected nanopores also facilitate the mass transfer activity of reactants and products for ORR.^[^ ^92^ ^]^

Huang et al.^[^ ^99^ ^]^ fabricated the synthesis of Fe−N‐doped double‐shelled hollow carbon microsphere (Fe−N–DSC) through in situ polymerization followed by pyrolysis. Exposure of more active sites for ORR on the Fe−N–DSC and a high flux mass transportation resulted in higher ORR performance than the commercial Pt/C catalyst, attributed to the special double‐shelled hollow carbon microsphere structure of the catalyst. As shown in **Table** **4**, the Fe−N–DSC showed a more positive half‐wave potential and similar onset potential to the Pt/C, which alludes to the proposition that Fe–N–DSC has better electrocatalytic activity than the Pt/C. Methanol and durability tests for Fe−N‐DSC were performed using chronoamperometric measurements, which revealed a sharp decay in current density for the Pt/C after injection of methanol which was not observed on the Fe–N–DSC catalyst. The above results allude that the Pt/C is more susceptible to CO poisoning than the Fe–N–DSC catalyst, which is a better candidate for use in DMFCs. The Fe–N–DSC catalyst also showed good durability under an acidic medium as chronoamperometric results showed a 94.3% retention of the initial current density after 30 000 s compared to 70.7% retention of the initial current density for the commercial Pt/C. Fe–N–DSC catalyst also had an electron transfer number of 3.96, suggesting that oxygen, the kinetic process for this catalyst, favors a direct four‐electron pathway for the ORR.

**Table 4:** Non‐noble‐based electrocatalysts synthesized using various synthetic routes and their ORR and alcohol oxidation protocols.^[^ ^98^, ^99^, ^100^, ^101^, ^102^ ^]^

Catalyst	Catalyst synthesis route	Electrolyte	1) ORR onset potential/half wave potential V versus RHE. 2) Mass activity/specific activity. 3) current density (*j* value).	Electron transfer number [n]	Tafel slope	Scan rate
SL Ti_3_C_2_	Liquid exfoliation process by combining HF etching (delamination) and TPAOH intercalation (disintegration)	0.1 m KOH	1) 0.85 V versus RHE (onset)	3.7	64 mV decade^−1^	50 mV s^−1^
Fe−N‐DSC	In situ polymerization followed by pyrolysis	0.5 m H_2_SO_4_	1) 0.608 V versus Ag/AgCl (onset). 0.456 V versus Ag/AgCl (half).	3.96	–	50 mV s^−1^
Fe/N/APC‐900	Ammonia presynthesis activation strategy	0.1 m KOH	1) 0.9 V versus RHE (onset). 0.88 V (half). 6.05 mA cm^−2^ (limiting current density).	3.97	30 mV decade^−1^	50 mV s^−1^
NiO@C/CC	Air‐assisted transient thermal shock strategy	1 m KOH + 1 m ethanol	119.9 mA cm^−2^ (current density).	–	–	50 mV s^−1^
Cu‐Co@N‐C	Hydrothermal process	1.0 m KOH + 1.0 m methanol	171.3 mA cm^2^ (current density)	–	–	50 mV s^−1^

Xi et al.^[^ ^100^ ^]^ fabricated the synthesis of hierarchically nanoporous Fe/N/APC‐900 by a feasible and straightforward ammonia presynthesis activation strategy. This strategy achieved highly efficient heme‐derived Fe/N/C electrocatalysts with highly exposed Fe−N sites. As shown in Table 3, the synthesized Fe/N/APC‐900, the iron and nitrogen‐doped ordered mesoporous carbon (APC), performed exceptionally for ORR in alkaline media as it possessed a positive half‐wave potential and a higher diffusion limiting current density in comparison to the conventional Pt/C. LSV curves suggest that this catalyst had exceptional onset (0.9 V) and half‐wave (0.88 V) potential, which was more positive to indicate that this catalyst was active toward ORR and comparable to that of the Pt/C (half‐wave potential of 0.85 V, onset potential 0.965 V). Also, the diffusion limiting current density of Fe/N/APC‐900 in 0.1 m KOH was 6.05 mA cm^−2^ at 0.3 V versus RHE, while that of the Pt/C was 5.0 mA cm^−2^. This shows that the Fe/N/APC‐900 catalyst, compared to Pt/C under the same electrolyte and same conditions, was less influenced by experimental conditions.

The high electrocatalytic activity on these Fe/N/APC‐900 catalysts was attributed to the synergistic effect of the exposed catalytic sites between high contents of Fe−N and pyridinic N, along with the fast mass transport properties arising from the etched highly permeable porous structure.^[^ ^100^ ^]^ The electron transfer number for the Fe/N/APC‐900 catalyst was 3.97, which was comparable to that of Pt/C, which was 3.95, indicating the desire toward a four‐electron process for oxygen reduction Fe/N/APC‐900 catalyst. Chronoamperometry was used to examine the durability of the Fe/N/APC‐900 catalyst, and continuous O_2_ reduction after 30 000 s at 0.81 V versus RHE resulted in only an 18% loss of current density before becoming constant. The results for the Pt/C showed current loss under the same conditions that were as high as ≈30%, which indicates better stability and durability of the Fe/N/APC‐900 relative to the Pt/C toward the ORR process. The enhanced ORR activity of the Fe/N/APC‐900 catalyst was also attributed to the smaller Tafel slope of 30 mV/decade at low overpotentials compared to that of the Pt/C (43 mV/decade) in 0.1 m KOH. The above results of the Tafel slope showed that the reaction kinetics of the catalyst were under the minimal influence of mass transfer.

The construction and fabrication of non‐noble metal‐based electrocatalysts that are highly active and durable are crucial requirements that will facilitate the further development and application of FCs. Liu et al.^[^ ^101^ ^]^ fabricated the synthesis of highly dispersed ultrathin carbon‐coated nickel oxide NPs settled on carbon cloth (1.0 M KOH + 1.0 m ethanol at 0.6 V (vs. saturated calomel electrode (SCE)) for 3600 s by an air‐assisted transient thermal shock strategy. This NiO@C/CC catalyst showed outstanding electrocatalytic performance for ethanol oxidation and even methanol and ethylene glycol oxidation in an alkaline medium and even outperformed most of the reported non‐noble metal catalysts. As shown in Table 4, the current density for the synthesized NiO@C/CC catalyst was 119.9 mA cm^−2^, significantly higher than its counterpart Ni@C/CC. The current ratio for the forward scan in relation to the backward scan (*I*_f_/*I*_b_) for EOR at NiO@C/CC (1.1) was 1.4 times higher than that of Ni@C/CC (0.8), which shows that they are fewer toxic intermediates that are produced for NiO@C/CC during the oxidation of ethanol. Chronoamperometry conducted in 1.0 m KOH + 1.0 m ethanol at 0.6 V (vs SCE) for 3600 s was used to examine the stability of the NiO@C/CC catalyst. A retention rate of 87% regarding the current density was observed after 3600 s, indicating the excellent electrocatalytic stability of the synthesized NiO@C/CC catalyst.

To date, continuous effort has been devoted to non‐noble‐based catalysts for MORs due to the low cost of such materials compared to Pt‐based electrocatalysts. Chen et al.^[^ ^102^ ^]^ fabricated the synthesis of Cu–Co nanocrystal stabilized on a nitrogen‐doped carbon matrix (Cu–Co@N–C) through a hydrothermal process. The Cu–Co@N–C composite showed excellent MOR due to the synergistic effect of Cu and Co since Co has a high activity for oxidation reactions, and Cu has a high adsorption capacity for methanol. As shown in Table 4, CV plots of the composite at a scan rate of 50 mv s^−1^ at 1 m KOH after the addition of 1 m methanol show a high specific MOR activity (*j* value) of 171.3 mA cm^2^ at 0.6 V versus SCE in an alkaline medium for the MOR which is significantly higher than that of the reported Pt/C (41.5 Ma cm^2^). The stability of the Cu–Co@N–C catalyst was measured utilizing chronoamperometry operated for 10 h, which revealed a decrease in the *j* value of the composite from 171.3 to 137.7 mA cm^2^ while that of the Pt/C was from 42 to 3 mA cm^2^. The above results indicated that large amounts of CO intermediate products were adsorbed on the surface of the Pt/C catalyst, which rendered the catalyst unusable during the MOR process. The durability of the Cu–Co@N–C composite was evaluated using CV plots which showed a retention rate of the *j* value of 77.6% at 0.6 V versus SCE after 1500 CV cycles which indicated that the catalyst could be reused over a long period of time for MOR. When the electrolyte was changed to the same as the original one, the retention rate recovered to almost 92.3%, indicating this catalyst's reusability.

## 8 Electrocatalysis

As alluded to earlier, a FC has two fundamental processes: alcohol electro‐oxidation and oxygen electroreduction. Alcohol electro‐oxidation is mainly referred to as AOR, while oxygen electroreduction is ORR. These two processes are equally important, as the overall FC performance is based on them. So, any sluggish execution of either one results in reduced FC performance. To achieve a high‐performance FC goal, the smart nanomaterial‐based electrocatalysts should mediate the activation energy to reduce energy consumption during bond breaking and formation. Higher kinetic rates are also needed to reduce the reaction electrocatalytic time.

The electrocatalysts should facilitate a complete reaction pathway. This need calls for a more profound understanding of either oxidation or reduction of reactants and intermediates formed. For example, in AOR, during the oxidation reaction, CO is produced, which poisons the electrocatalysts. An H_2_O_2_ intermediate is created in ORR, which can poison the electrocatalyst or interfere with the electrokinetics at the cathode side, resulting in sluggish ORR. Thus, smart nanomaterials should enhance performance and mitigate unwanted reactions and intermediates.

### 8.1 Alcohol Oxidation Reaction (AOR)

The use of alcohols as fuels in FCs has become popular compared to hydrogen. They are relatively cheaper, have a high volumetric energy density, and are easily stored and transported. Platinum or platinum‐based electrocatalysts have proved to be the best performing toward AOR.

Different alcohol‐based FCs have been developed. These include, but are not limited to, direct methanol fuel cells (DMFCs), direct ethanol fuel cells (DEFCs), direct ethylene glycol fuel cells (DEGFCs), direct glycerol fuel cells (DGEFCs). The alcohol name within the FC name represents the alcohol used as the fuel for that specific FC. For the listed FCs, the fuels are methanol, ethanol, ethylene glycol, and glycerol, respectively. These are monohydric (e.g., methanol, ethanol) and polyhydric (e.g., ethylene glycol, and glycerol). Two electrolytes have been used for the oxidation of these two classes of alcohols. These are sodium hydroxide (NaOH) and potassium hydroxide (KOH). KOH has emerged to be the most used of the two as it is less corrosive and very conductive and reduces Ohmic overpotential loss than NaOH. The usage of KOH or relatively alkaline conditions enables the usage of non‐noble electrocatalysts.

There should be unceasing electrocatalyst research for FCs’ highly outstanding efficiency of the electro‐oxidation process. The development of AOR electrocatalysts should be so that they can effectively catalyze the activation of the C—C bond cleavage and facilitate the complete oxidation of the alcohols to carbon dioxide. The complete oxidation will allow the release of the maximum theoretical number of electrons. Equation (1) and (2) represent the fast dissociative adsorption of R–CH_2_OH. The complete oxidation of alcohols requires an extra oxygen atom. This extra oxygen atom comes from the adsorbed hydroxyl groups (OH^−^) (Equation (3) and (4)) formed from water chemisorption. Some of the OH^−^ comes from the ORR occurring at the cathode. This postulation can be well represented by the Pd system, which has proved to be very stable in the alkaline medium.^[^ ^103^ ^]^ Using Pd in alkaline media opens room for employing transition metal oxides as promoters, different defected carbons, and heteroatom‐doped support for well‐engineered electrocatalysts. Thus, there is better kinetic oxidation of the reaction.

(1) $$\text{Pd}+{\text{RCH}}_{2}\text{OH}\to \text{Pd}–{\left({\text{RCH}}_{2}\text{OH}\right)}_{\text{ads}}$$

(2) $$\text{Pd}–{\left({\text{RCH}}_{2}\text{OH}\right)}_{\text{ads}}+{\text{3OH}}^{-}\to \text{Pd}–\left(\text{RCO}\right)\text{ads}+{\text{3H}}_{2}O+{\text{3e}}^{-}$$

(3) $$\text{Pd}–\text{OH}\to \text{Pd}–{\text{OH}}_{\text{ads}}+e^{-}$$

(4) $$\text{Pd}–{\left(\text{RCO}\right)}_{\text{ads}}+\text{Pd}–{\text{OH}}_{\text{ads}}\to \text{Pd}–\left(\text{RCOOH}\right)+\text{Pd rds}$$

(5) $$\text{RCOOH}+{\text{OH}}^{-}\to {\text{RCOO}}^{-}+H_{2}O$$

Value‐added products (VAPs) can be obtained in FCs with a cogeneration system, for example, R–COOH or R–COO^−^ (Equation (4) and (5)). The rate‐determining step, Equation (4), shows that the catalytic precedence of AOR is mainly dependent on the surface coverage of the electrocatalysts by adsorbed species and the concentration of adsorbed OH^−^.

#### 8.1.1 Alcohol Electro‐Oxidation Reaction Mechanisms

DAFCs are better candidates than fossil fuel combustion for producing electrical energy. The lack of efficient electrocatalysts to boost the anodic electro‐oxidation of alcohol by cleaving the C—C bond to facilitate the oxidation of alcohol to carbon dioxide urgently needs to be addressed.^[^ ^104^ ^]^ Platinum‐based NPs are still the excellent catalysts for the electro‐oxidation of simple and polyalcohols with fast kinetics over many precious and nonprecious metal NPs when employed as electrocatalysts. However, the high cost and low electrochemical stability of platinum induced by easy chemical adsorption of carbonaceous intermediates, mainly known as carbon monoxide, which strongly attacks or adsorbs on the active sites of platinum responsible for electro‐oxidation of alcohol at the anode compartment, are still the main challenges which severely hinder the utilization of DAFCs in large‐scale applications.^[^ ^105^ ^]^ It has been confirmed that CO is the primary inhibitor during the electro‐oxidation of many alcohols. It chemically adsorbs onto the active sites of platinum, responsible for the complete electro‐oxidation of alcohol to carbon dioxide.

What causes this chemisorption of CO into active platinum sites? The electron donation causes this strong chemical bond from the 3σ molecular orbital to the d*σ* band of platinum. Therefore, platinum will back donate more electrons from its d*π* orbital to the electron‐free 2*π* antibonding orbital of carbon monoxide, making this bond between platinum and carbon monoxide stronger.^[^ ^106^ ^]^ In the last 10 years, many engineering strategies have been conducted to improve platinum's electrocatalytic activity, durability, and performance toward the anodic electro‐oxidation of alcohol. To counteract the lower stability of platinum when used as an electrocatalyst for electrochemical alcohol oxidation, strategic plans such as the fabrication of Pt‐M alloy NPs showed significantly increased catalytic activity toward alcohol electro‐oxidation compared to a monometallic Pt electrocatalyst. This unique electrocatalytic activity towards alcohol electro‐oxidation is ascribed from two phenomena generally known as bifunctional mechanism and electronic effect.^[^ ^105^, ^106^, ^107^ ^]^

##### Bifunctional Mechanism

The bifunctional mechanism is the addition or introduction of second or third metal (M) to help the desorption of chemically adsorbed species on the surface of an electrode which also contributes to the enhanced electrocatalytic activity of alcohol electro‐oxidation. The second metal addition to the monometallic NPs results in a significant synergetic effect, resulting in the eventual desorption of carbon monoxide from active platinum sites for further electrochemical oxidation to carbon monoxide. How does the carbon monoxide intermediates desorb from the surface of the platinum electrocatalyst? The desorption of carbon monoxide intermediates is induced by the synergistic interaction of the second metal to platinum (forming the Pt—M bond), which causes less electron density between the Pt—CO bond. Pt back donates fewer electrons to the empty 2π antibonding orbitals of CO; hence, the synergistic interaction of the Pt—CO bond loses its stabilizing effect. Platinum is back donating more electrons to the second metal's orbitals, thus strengthening the Pt—M bond and weakening the Pt—CO bond, which contribute to the desorption of CO intermediates for further electro‐oxidation process, leaving more active sites of platinum.^[^ ^106^, ^107^, ^108^ ^]^

At low potentials on the metal surfaces of bimetallic systems (Pt—M), water's second substrate produces oxophilic species (M). This allows partial oxidation of intermediates to be removed from the surface more efficiently, reducing surface poisoning.^[^ ^109^ ^]^ According to the bifunctional process, methanol is dissociated and adsorbed on Pt before degrading into adsorbed CO and/or formyl‐like species –CHO_ads_ through a dehydrogenation reaction (Equation (6)). Water splits into OH* and is adsorbed on M sites simultaneously (Equation (7)). The species then interact on Pt and M sites to produce CO_2_ molecules. CO_2_ is formed due to the reaction between Pt–CO_ads_ and M–OH_ads_, resulting in poison‐free Pt and M sites (Equation (8)). The MOR on supported binary PtRu alloy electrocatalysts can be stated as the following equations using the bifunctional mechanism.^[^ ^105^, ^110^, ^111^ ^]^

(6) $$\text{Pt}+{\text{CH}}_{3}\text{OH}\to \text{Pt}-{\text{CH}}_{3}{\text{OH}}_{\text{ads}}\to {\text{Pt}–\text{COH}}_{\text{ads}}\to {3H}^{+}+{\text{3e}}^{-}\to \text{Pt}-{\text{CO}}_{\text{ads}}+{\text{ H}}^{+}+e^{-}$$

(7) $$M+H_{2}O\to M–\text{OH}+H^{+}+e^{-}$$

(8) $$\text{Pt}–{\text{CO}}_{\text{ads}}+M–{\text{OH}}_{\text{ads}}\to \text{Pt}+M+{\text{CO}}_{2}+H^{+}+e^{-}$$

Since pure Pt shows a rapid poisoning effect during anodic electro‐oxidation of alcohols due to the production of toxic intermediates, the introduction of second and third oxophilic transition metals, such as Ru, Mo, Sn, Pd, Rh, has been discovered to be highly beneficial because the oxophilic metals' intermediate, M–OH. The M–OH may destroy Pt–CO via an electrochemical reaction in an acidic medium depicted in Equation (3).^[^ ^112^ ^]^ Marinho et al.^[^ ^113^ ^]^ investigated the addition of palladium as second metal to platinum toward ethylene glycol electro‐oxidation in alkaline media. CV and chronoamperomtery tests were used to study the EGOR in an alkaline medium on PtPd/C. PtPd/C had the highest ethylene glycol electro‐oxidation catalytic activity and extraordinary antipoisoning ability compared to Pt/C electrocatalyst. This was explained by Pd, promoting a higher glycolate or oxalate formation. Souza et al.^[^ ^114^ ^]^ incorporated niobium as a palladium promoter for the enhanced catalytic activity of Pd/C electrocatalyst toward ethanol electro‐oxidation. This study found that the PdNb/C electrocatalyst possessed mass activity for the EOR of 1.7 times greater than that of the pristine Pd/C electrocatalyst. It was also observed that palladium electrocatalyst with niobium shifted the EOR toward low onset potentials than pure Pd/C electrocatalyst, implying PdNb/C electrocatalyst was more electrocatalytic active with high current density and poison tolerance toward EOR than pure Pd/C electrocatalyst. This significant catalytic activity was attributed to the bifunctional mechanism and modification of electronic structure and properties of palladium induced by alloying with Nb. Niobium existed as Nb_2_O_5_, functioning as an oxophilic substance to adsorb water and then producing OH* which is essential for the removal and oxidation of carbon monoxide intermediates to carbon dioxide, thus freshening Pd active sites, as shown in Equation (9) and (10), respectively. The bifunctional mechanism that arises between niobium and palladium interaction may be represented as follows.

(9) $${\text{Nb}}_{2}O_{5}+H_{2}O\to {\text{Nb}}_{2}O_{5}-\text{OH}+H^{+}+e^{-}$$

(10) $$\text{Pd}–\text{CO}+{\text{Nb}}_{2}O_{5}–\text{OH}\to \text{Pd}+{\text{Nb}}_{2}O_{5}+{\text{CO}}_{2}+H_{2}O$$

Yan et al.^[^ ^115^ ^]^ studied the incorporation of copper with palladium through a simple solvothermal technique; a self‐supported PdCu alloy nanowire (NW) electrocatalyst was successfully prepared with the help of Cu. The presence of Cu assisted in modifying the morphology of the Pd‐based catalyst from graininess to the NW. It was found that PdCu/C electrocatalysts exhibited a distinctive catalytic activity with enhanced poison tolerance and durability toward ethylene glycol electrochemical oxidation compared to a commercial Pt/C electrocatalyst. PdCu/C electrocatalyst also showed a higher electrochemical surface area than commercial Pd/C catalysts due to its self‐supported structure.

In another study, Ahmadi et al.^[^ ^116^ ^]^ evaluated the importance of alloying Pt with Co as an effective electrocatalyst to catalyze methanol electro‐oxidation reaction. They found that bimetallic sulfur‐modified CNTs‐decorated PtCo nanoalloys electrocatalysts exhibited high catalytic activity and poison tolerance against carbon monoxide intermediates toward MOR compared to commercial Pt/C electrocatalysts. The effect of Co as a Pt promoter was revealed by the shift toward a low onset potential of ≈212.0 mV compared to a monometallic Pt/CNT electrocatalyst around 240.0 mV. The high MOR activity was highly induced by incorporating Pt with Co as a metal promoter, which also modified the electronic structure and properties of platinum, thus freshening active platinum sites for adsorption of methanol by removing carbon monoxide intermediates that were formed during electro‐oxidation of methanol. Santos et al.^[^ ^117^ ^]^ studied the improved CO_2_ selectivity during the electrochemical oxidation of ethanol on carbon‐decorated PtPb NPs. PtPb/C was synthesized using a simple approach, including formic acid as the reduction agent; they effectively generated carbon‐supported PtPb/C core–shell electrocatalysts. CO monolayer and ethanol oxidation electrochemistry tests revealed that the PbPt/C electrocatalyst performed better electrocatalytically. In addition to electrochemical studies, in situ, Fourier‐transform infrared spectroscopy investigations revealed that acetic acid was the major product of ethanol electro‐oxidation on the investigated catalysts. The core–shell catalysts PbPt/C produced more CO_2_, implying more selectivity for C—C bond breaking. These results can be attributed to combining the electrical and geometric effects of the core–shell structure. When Sn was incorporated into the bimetallic electrocatalyst to form the PtPbSn/C electrocatalyst, it also outperformed the electrocatalytic activity of the PtPb/C electrocatalyst and also showed the best poison tolerance. The poison tolerance was ascribed to the bifunctional mechanism facilitated by third metal in the shell. Ding et al.^[^ ^118^ ^]^ also studied the hydrothermally synthesized trimetallic PdNiAl nanocomposites as an active electrocatalyst for ethanol electro‐oxidation. Even though Al content was fixed, it was noted that the addition of aluminum on the PdNi/MCNTs composite was found to increase the catalytic activity and performance of the bimetallic catalyst system toward ethanol electro‐oxidation reaction. It also revealed that the addition of Al has a positive effect on the long‐term stability and poison tolerance of the PdNiAl/MCNTs electrocatalyst.

Chen et al.^[^ ^119^ ^]^ also investigated the application of a sub‐1 nm PtSn ultrathin sheet as an exceptional electrocatalyst for both methanol and ethanol electro‐oxidation. In conclusion, we established a simple colloid production process for ultrathin PtSn nanosheets with thicknesses ranging from 0.6 to 0.9 nm. PtSn nanosheets not only exhibited an outstanding mass activity in the MOR (871.6 mA/mgPt), which was 10.1 times (86.1 mA/mgPt) higher than commercial Pt/carbon and Pt black, but also showed an outstanding mass activity in EOR (673.6 mA mgPt^−1^), which is 5.3 times higher than commercial Pt black electrocatalyst (127.7 mA mgPt^−1^). PtSn nanosheets exhibited good electro‐oxidation properties for MOR and EOR in both acidic and alkaline mediums, owing to their ultrathin 2D structure, which dramatically enhances the cleavage of C—C bonds while also improving the removal of CO intermediate species formed on the platinum surface during alcohol electro‐oxidation. A similar study was conducted by Puthiyapura et al.,^[^ ^120^ ^]^ where they investigated the use of bimetallic PtSn/C electrocatalysts toward isomeric butanol electro‐oxidation reaction. They found that commercial Pt/C electrocatalysts could not oxidize both butanol isomers when employed as the electrocatalyst. However, for PtSn bimetallic catalysts, the oxidation of primary isomers of butanol exhibited a substantial improvement, with an oxidation peak at potentials between 0.25 and 0.30 V, which is at a significantly lower overpotential than monometallic Pt/C electrocatalyst. Nonetheless, no substantial effect was detected for secondary and tertiary butanol isomer when Sn was added to Pt. The bifunctional process linked with the bimetallic electrode could account for the greater activity on Sn in addition to Pt for the electro‐oxidation of primary butanol.

The effect of iridium as a metal promoter boosts the electrocatalytic activity of bimetallic PtSn/C electrocatalyst to facilitate ethanol electro‐oxidation reaction. It was found that the addition of Ir to PtSn/C electrocatalyst contributed to the high catalytic electrochemical conversion of ethanol to carbon dioxide compared to bimetallic and monometallic PtSn/C and Pt/C, respectively. It was also found that trimetallic PtSnIr/C electrocatalyst exhibited high current density and poison tolerance. It was found to possess high electrochemical stability over a long period of time than PtSn/C and Pt/C when tested against chronoamperometry.^[^ ^121^ ^]^ Lan et al.^[^ ^122^ ^]^ also studied an application of PtNiCu nanoalloys as an effective electrocatalyst to boost the methanol electro‐oxidation reaction. They found that increasing the percentage of Cu resulted in high catalytic activity and stability toward the methanol electro‐oxidation reaction. It was seen that PtNiCu/C electrocatalyst exhibited a current density of about two‐ and eight‐fold greater than that of bimetallic PtCu/C and Pt/C electrocatalyst when examined in CV, respectively. Several defects of grain boundary, low coordinated atom, and lattice disorder possessed by PtNiCu/C electrocatalysts were the main reasons for the enhanced electrocatalytic activity toward methanol electro‐oxidation reaction. Second, it was concluded that the synergistic interaction between Pt, Cu, and Ni induced this substantial electrocatalytic activity, and the performance of PtNiCu/C electrocatalyst toward MOR helps in lowering the degree of chemisorption of carbon monoxide intermediates. Bergamaski et al.^[^ ^123^ ^]^ investigated the effect of incorporating platinum with rhodium NPs as an efficient electrocatalyst for ethanol electro‐oxidation. They also found that rhodium played a vital role in boosting platinum's electrocatalytic activity and stability. They also noted that incorporating Pt with Rh has caused the shift in the efficiency of the pristine platinum electrocatalyst to move from 0.08 to 0.5. The addition of Rh improved the electrocatalytic conversion of ethanol to carbon monoxide by tuning the electronic properties of Pt.

Other researchers in the past decades investigated the effect of using molybdenum as a metal promoter on platinum–ruthenium electrocatalyst toward MOR. PtRuMo/C electrocatalysts showed much enhanced electrocatalytic activity with high poison tolerance than PtRu/C electrocatalysts. It possessed a current density of approximately two times than that of one of the PtRu/C electrocatalysts. The improved performance of PtRuMo/C NPs electrocatalyst compared to PtRu/C has been attributed to the Mo additive, which can be investigated from three perspectives: the bifunctional mechanism, the hydrogen‐spillover aspect, and the adjustment of Pt electronic states. It was found that MoO_*x*_ promotes water activation, which results in the formation of OH* species capable of oxidizing CO to CO_2_. This explained why the PtRuMo/C NP catalyst possessed a higher catalytic activity during the ethanol electro‐oxidation process.^[^ ^124^ ^]^ Also, Bi NPs have been studied as promoters on PtRu/C electrocatalysts toward ethylene glycol electro‐oxidation reaction (EGOR). The hydrothermal and liquid impregnation methods were used to prepare a 3D‐reduced graphene oxide/MOF‐199 successfully supported Pd–Ru–Bi nanoalloy electrocatalyst with high activity and durability for EGOR. When the PdRuBi/rGO/MOF‐199 catalyst was used as an anode electrocatalyst for EGOR, the current density was 7.23 times superior to that of commercial Pd/C, with values of ≈213.97 and 29.57 mA cm^−2^ respectively. The results show that incorporating reduced graphene oxide/MOF‐199 support into a catalyst can provide a large surface area, superior electron transfer, and abundant active reaction sites for Pd–Ru–Bi nanoalloys. Furthermore, the significant trimetallic synergistic effect would increase the surface adsorption of OH* and make the oxidation of the forming intermediates on Pd surfaces easier. These factors contribute to an improved PdRuBi/rGO/MOF‐199's electrocatalytic activity, including a lower oxidation onset potential, high current density, and electrochemical stability. The catalytic activity of the as‐synthesized electrocatalyst was in the following order: PdRuBi/rGO/MOF‐199 > PdRu/rGO/MOF‐199 > PdBi/rGO/MOF‐199 > Pd/rGO/MOF‐199 > commercial Pd/C. This shows how the synergistic interaction of Ru and Bi species improved the stability and catalytic activity of the EGOR.^[^ ^125^ ^]^

##### Intrinsic Mechanism

This mechanism is also known as the ligand effect. It involves the synergistic interaction between the base metal and the second metal, which modifies the electronic properties of the base metal. The addition of the second or third metal to the base metal NPs causes a huge effect in increasing the electrochemical stability, electrocatalytic activity, performance, and poison tolerance against carbonaceous intermediates such as carbon monoxide. Such an effect can be observed where the presence of a second metal weakens the bond strength of Pt—CO due to an intrinsic interaction between the two metals in which the bond strength of Pt—M is strengthened, thereby weakening the Pt‐CO bond leading to desorption of CO which is further oxidized to carbon dioxide leaving free Pt active sites for adsorption of methanol or any alcohol. For alcohol oxidation processes, bimetallic catalysts are often more active than monometallic catalysts. The bifunctional mechanism and ligand effect induce this during alcohol electro‐oxidation.

Han et al.^[^ ^126^ ^]^ investigated the addition of Sn as a promoter in platinum electrocatalysts for methanol electro‐oxidation reaction. This research demonstrated that PtSn supported on graphene exhibited distinctive electrocatalytic activity with high current density, high poison tolerance, and low onset potential compared to monometallic platinum supported on graphene electrocatalyst. Wang et al.^[^ ^127^ ^]^ also studied the effectiveness of reduced graphene oxide‐decorated trimetallic PdNiAu electrocatalyst toward EOR in alkaline media. They found that trimetallic palladium‐based electrocatalysts exhibited superior catalytic activity of high current density, long‐term stability, and good performance with a high antipoisoning effect compared to the commercial Pd/C electrocatalyst. This unique activity was attributed to the ability of gold to change the electronic properties of palladium core–shell. Furthermore, it was revealed that gold could also eliminate the chemisorbed carbonaceous species, such as carbon monoxide, from palladium's surface, leaving more palladium actives sites. It was also found that the catalytic activity of trimetallic palladium‐based electrocatalysts was four times greater than that of monometallic palladium electrocatalysts. When comparing the activity of bimetallic electrocatalysts, namely, PdAu and PdNi supported on reduced graphene it was found that PdAu/rGraphene showed better catalytic activity and electrochemical stability compared to PdNi/rGraphene toward ethanol electro‐oxidation. This also reveals that gold is a better oxophilic metal or promoter capable of tuning Pd's electronic structure and properties than Ni as a promoter. The catalytic activity of all palladium‐based electrocatalysts was found to be in this order toward ethanol oxidation reaction (EOR), PdNiAu/rGraphene > PdAu/rGraphene > PdNi/rGaphene > Pd/rGraphene > commercial Pd/C electrocatalyst. Roy et al.^[^ ^128^ ^]^ also studied the synergistic interaction between silver and palladium toward ethanol electro‐oxidation activity in an alkaline environment. They found that the addition of silver NPs to Pd altered the electronic nature of Pd and further boosted the catalytic activity and poison tolerance of PdAg/C toward ethanol electro‐oxidation. Notably, Ag helps Pd in forming nonadsorbed species such as acetates than carbonates, thus boosting the antipoisoning ability of palladium during the ethanol electro‐oxidation process.

#### 8.1.2 Electrocatalytic Oxidation of Monohydric Alcohols

##### Methanol Oxidation Reaction (MOR)

MOR is the most studied alcohol in AOR. The MOR is favored due to methanols' simple molecular structure (C1–alcohol), ease of cleaving the C—H bond, and high electrokinetics (six electrons in the electrocatalytic process). However, apart from these merits, its usage is much feared because of its toxicity. MOR is complex as it depends on the electrocatalyst's structural architecture, coupled with electrolytes' pH, temperature, and concentration. The overall MOR reaction can be written as shown in Equation (11).

(11) $${\text{CH}}_{3}\text{OH}+{\text{6OH}}^{-}\to {\text{CO}}_{2}+{\text{5H}}_{2}O+6e^{-}$$

This overall reaction is believed to occur mainly because of methanol adsorption and carbon monoxide and CO oxidation. However, alkaline‐dependent MOR is a multistep process in a real FC. Cohen et al.^[^ ^129^ ^]^ proposed that the MOR occurs via Equation (12) and (13).

(12) $${\text{CH}}_{3}\text{OH}+{\text{4OH}}^{-}\to {\text{CO}}_{\text{ads}}+{\text{4H}}_{2}O+4e^{-}$$

(13) $${\text{OH}}^{-}\to {\text{OH}}_{\text{ads}}+e^{-}$$

(14)

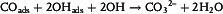




There have been studies that have confirmed that the route of MOR proceeds via some intermediates. For example, Santasalo‐Aarnio et al.^[^ ^130^ ^]^ formed significant formaldehyde and formate on Pt and Pd electrodes. An independent study by Tripkovic et al.^[^ ^131^ ^]^ revealed the formation of formate. Interestingly, the independent teams showed that formate is indeed an intermediate. The formation of both formaldehyde and formate is interlinked to the surface adsorbed intermediates as shown below.

(15) $${\text{CH}}_{3}\text{OH}+{\text{3OH}}^{-}\to {\text{COH}}_{\text{ads}}+{\text{3H}}_{2}O+3e^{-}$$

(16) $${\text{CH}}_{3}\text{OH}+{\text{OH}}^{-}\to {\text{CH}}_{3}O_{\text{ads}}+H_{2}O+e^{-}$$

(17) $${\text{CH}}_{3}\text{OH}\to {\text{CH}}_{3}{\text{OH}}_{\text{ads}}$$

From Equation (15), the OH^−^ facilitates the deprotonation of the CH_3_OH molecules, resulting in the formation of COH adsorbed intermediate species and three water molecules. Likewise, in Equation (16), the hydroxyl enhances partial deprotonation, leading to the formation of adsorbed methoxy, CH_3_O_ads_, one molecule, and an electron. However, in some cases, there will be direct adsorption of methanol molecules on the electrocatalyst surface, as shown in Equation (17). These adsorbed intermediates are responsible for forming formaldehyde and formate.^[^ ^130^, ^131^ ^]^ However, these are then further oxidized to carbon dioxide.

With this generalized MOR, different electrocatalysts have been developed. Platinum‐based electrocatalysts proved to be the best, with high electrocatalytic activity. In that regard, Gwebu et al.^[^ ^132^ ^]^ synthesized Pt/carbon nanodots electrocatalyst, which had 8.12% of Pt, as determined by inductively coupled plasma–optical emission spectroscopy (ICP–OES). This study reported higher methanol oxidation due to the high CH_3_OH and OH–concentration. To improve MOR kinetics, researchers adopted bifunctionality electrocatalysis; this has been popularly known as alloying. The promotional effect of Ru on platinum‐based electrocatalysts has been well studied. Herrero et al.^[^ ^133^ ^]^ performed CV to show that the activity could be assigned to the adsorbed hydroxyl species on Ru in the composite electrocatalyst. They coined the activity of the highly active adsorbed hydroxyl species to the highly effective bifunctionality nature of Pt (110)/Ru electrocatalyst surface. The nature and role of Ru in alloyed electrocatalysts have been debatable. In that regard, it can be postulated that metallic Ru is responsible for enhancing MOR electrocatalytic activity.

However, it has been proven experimentally by Rolison et al.^[^ ^134^ ^]^ and Ma et al.^[^ ^135^ ^]^ that for alloyed Pt and Ru electrocatalysts, RuO_*x*_H_*y*_ is more electrocatalytically active as compared to metallic ruthenium. A commendable MOR performance has been reported by Cao et al.,^[^ ^136^ ^]^ who used hydrous CNTs‐RuO_2_–Pt. Conclusively, high electrocatalytic MOR is enhanced by the interlinkage of RuO_*x*_H_*y*_ and Pt in terms of relative interplanar distance and RuO_*x*_H_*y*_ amount. Many electrocatalysts architectural designs for improving MOR have been reported.^[^ ^137^, ^138^, ^139^ ^]^ Therefore, the overall enhancement of the MOR is based on metallic cooperation and support material synergy with the electrocatalyst metallic component.

**Figure 4:**
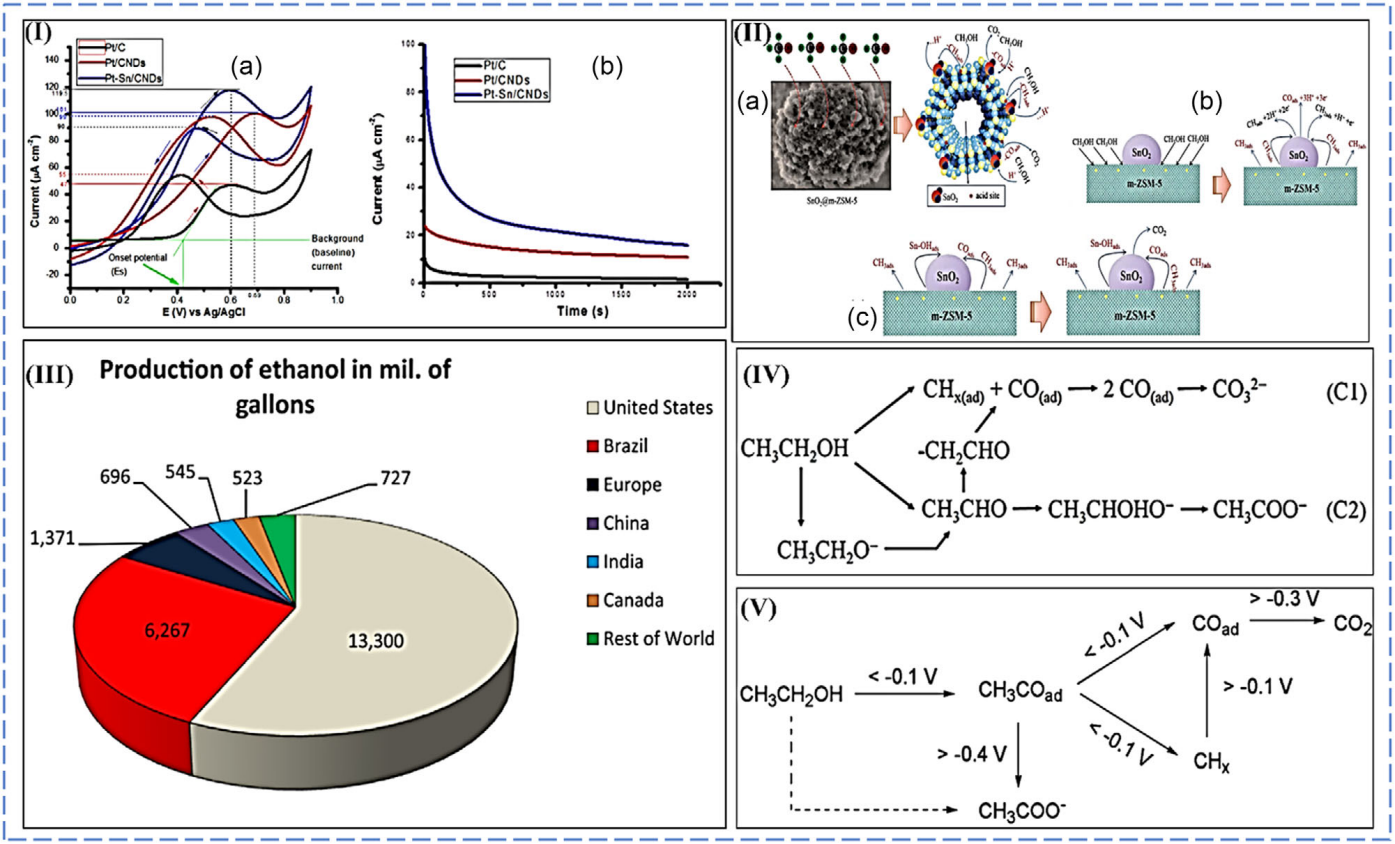
I: a) Cyclic voltammograms and b) chronoamperometric curves of Pt/C, Pt/CNDs, and Pt‐Sn/CNDs in 0.5 m CH_3_OH + 0.5 m H_2_SO_4_ electrolyte. Reproduced with permission.^[^ ^141^ ^]^ Copyright 2018, Wiley; II: a) CH_3_OH adsorption on to SnO_2_/*m*‐ZSM‐5; b) synergic electrocatalytic effect during MOR; and c) intermediate species oxidation to CO_2_.Reproduced with permission.^[^ ^142^ ^]^ Copyright 2015, Royal Society of Chemistry; III: Pie chart showing worldwide ethanol production;^[^ ^311^ ^]^ IV: EOR scheme (alkaline). Reproduced with permission.^[^ ^312^ ^]^ Copyright 2010, Elsevier. V: EOR on palladium surface (alkaline medium).^[^ ^155^ ^]^ Reprinted with permission.^[^ ^35^ ^]^ Copyright 2019, American Chemical Society.

In other studies, the alloying metal has been with Sn. Sn as an alloying metal has gained much attention as there have been reports of electrocatalysts with CO tolerance improvement.^[^ ^140^ ^]^ However, an adequate amount of Sn was said to be ≈25% for the notable and efficient bifunctional and well‐balanced electronic coupling. This means that there might not be enough Sn catalytic sites at lower weight percentages, hence reducing the oxidizing power of the adsorbed intermediate species. However, in their study, Gwebu et al.^[^ ^141^ ^]^ reported the synthesis of Pt–Sn/CNDs by an alcohol reduction reaction. They concluded 2.28% of Sn in the composite and high activity toward MOR and stability, as shown in **Figure** **4**Ia. This was attributed to the synergistic effect interaction of Pt–Sn and CNDs. From XPS results, there was a significant amount of SnO.

This complimented some research done by Shi et al.^[^ ^142^ ^]^ who showed that SnO_2_/*m*ZSM‐5 possessed high electrocatalytic properties for MOR coupled with a high CO tolerance. The high electrocatalytic activity toward MOR gave SnO_2_ ability to efficiently react with OH^−^ forming Sn (OH)_*x*_. The formed tin hydroxide, Sn(OH)_*x,*_ would reversibly release OH^−^. This reversible release of OH^−^ regenerates SnO_2_ (Equation (18)), and the MOR mechanism is shown in Figure 4IIa–c.

(18) $${\text{SnO}}_{2}+{\text{OH}}^{-}+H_{2}\text{O Sn}{\left(\text{OH}\right)}_{x}+e^{-}$$

These efforts improved the electrocatalyst toward MOR, improving sluggish kinetics and poisoning tolerance.

Due to the high costs of platinum and its outweighed stability in an alkaline medium, Pd electrocatalysts have been used mainly alloyed with Ni and/or NiO. The usage of Ni can be attributed to its ease in forming nickel hydroxide, Equation (19) and (20), which adds more hydroxide species to the reaction system. NiO is also said to enhance the poisoning resistivity of the electrocatalyst. Fleischmann's team proposed a mechanism of alcohol oxidation to carboxylic acids.^[^ ^143^ ^]^

(19) $$\text{Ni}{\left(\text{OH}\right)}_{2}\to \text{NiOOH}+H^{+}+e^{-}$$

(20) $${\text{RCH}}_{2}\text{OH}+\text{NiOOH}\to \text{RCOOH}+\text{Ni}{\left(\text{OH}\right)}_{2}$$

Such a type of reaction mechanistic reaction pathway has also been reported to use nonprecious metal‐based spinel oxides systems by Wu et al.^[^ ^144^ ^]^ Co_3_O_4_/NiO and Anu‐Prathap et al. studied^[^ ^145^ ^]^ NiCo_2_O_4_. They showed the possibility of non‐PGMs usage in MOR.

With all the efforts in evaluating the electrocatalyst performance, the CV does not give detailed mass transfer capability of the electrocatalysts. Many electrocatalysts have been developed for trial in MOR. These include binary systems, Rh–Ni,^[^ ^146^ ^]^ Pt–Cu,^[^ ^147^ ^]^ Pt–Mo,^[^ ^148^ ^]^ and ternary systems Pt–Ru–Co.^[^ ^149^ ^]^ There have been reports on quaternary systems, Pt–Ru–Ir–Os,^[^ ^150^ ^]^ Pt–Ru–Ni–Zr,^[^ ^151^ ^]^ etc. Thus, there is undoubtedly a need to continuously evaluate the electrocatalyst electrocatalytic performance using polarization curves in DAFCs.

##### Ethanol Oxidation Reaction (EOR)

Ethanol, a C‐2 type of alcohol, has gained much attention in the past years. This is because, compared to the other monohydric alcohol, CH_3_OH, ethanol is less toxic. Theoretically, ethanol produces 8.0 kW h ^−1^ kg ^−1^ versus 6.1 kW h ^−1^ kg ^−1^ for CH_3_OH^[^ ^152^ ^]^ and has a lower cost as it can be obtained continuously from biomass. Different countries have long focused on ethanol production, the leading country being the United States of America, as shown in Figure 4III.

The electrochemical EOR has been reported to be very complex. Hence, a close research focus is needed to fully achieve complete ethanol oxidation and its subsequent release of 12 electrons.^[^ ^38^ ^]^ This points to a vital need for a well‐developed synergistic interlink between the electrocatalysts synthesis techniques and theoretical simulations. The synergy development helps understand electrocatalyst architectures, meaning clear and well‐understood electrocatalysts' intrinsic properties. The EOR has had ongoing debates in the research community. This is usually on the mechanism pathway. However, there have been converging proposals pointing to the dual pathway,^[^ ^22^, ^153^ ^]^ commonly referred to as the C1 and C2 pathways.^[^ ^154^ ^]^ These two pathways are intermediary routes to the complete EOR, which releases 12 electrons, as shown in Equation (21).

(21) $${\text{CH}}_{3}{\text{CH}}_{2}\text{OH}+{12\text{OH}}^{-}\to {2\text{CO}}_{2}+9H_{2}O+12e^{-}$$

The dual C1 and C2 pathways, Figure 4IV, are inherent in equal or more complex than the MOR. The C1 pathway results in complete EOR as there will be the full bond breaking of the intermediary species. As for the C2 pathway, there will be incomplete oxidation. Both the C1 and C2 pathways are heavily dependent on the electrocatalytic activity of EOR electrocatalysts.

##### C1 Pathway

The breaking of a C—C bond mainly characterizes this C1 pathway. The C1 pathway leads to the formation of CO, CH_*x*_, fragments, or carbonates which will be sequentially oxidized to CO_2_ (CH_3_CH_2_OH → CH_3_CHO_ads _→ CO_ads_, CH_*x*_, _ads _→ CO_2_/CO_3_^2−^). This releases 12 electrons, as shown in Equation (22) and (22). The occurrence of the C1 pathway proceeds via the C—C bond activation. This pathway is not commonly experienced as the C—C bond breaking is sterically hindered due to the ethanol's atoms' orientation. Also, the formation of CO_ads_ on the electrocatalysts’ surfaces causes poisoning of the electrocatalysts. This significantly affects the Faradaic efficiency due to the loss in charge transfer. There is a need for smart nanomaterial‐based electrocatalysts that favor CH_3_CH_2_OH and C—C bond cleavage.

(22) $${\text{CH}}_{3}{\text{CH}}_{2}\text{OH}+{3H}_{2}O\to {2\text{CO}}_{2}+{12H}^{+}+{12e}^{-}$$

(23)

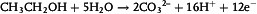




##### C2 Pathway

As opposed to the C1 pathway, the C—C bond is not cleaved; hence, there is incomplete oxidation, thus releasing two electrons, Equation (24), or four electrons, Equation (25), The resulting products are acetaldehyde (CH_3_CH = O), acetate, and acetic acid (CH_3_COOH); thus, CH_3_CH_2_OH → CH_3_CH_2_OH_ads_ → CH_3_CHO → CH_3_COOH. The occurrence of the C2 pathway proceeds via the C—H and O—H bond activation.

(24) $${\text{CH}}_{3}{\text{CH}}_{2}\text{OH}\to {\text{CH}}_{3}\text{CHO}+2e^{-}+2H^{+}$$

(25) $${\text{CH}}_{3}{\text{CH}}_{2}\text{OH}+H_{2}O\to {\text{CH}}_{3}{\text{CO}}_{2}H+4e^{-}+4H^{+}$$

The formation of the intermediates and products of EOR are also dependent on applied electrode potential.^[^ ^155^ ^]^ For the C1 pathway, the applied voltage is greater than 0.4 V, while for the C2 voltage, it is less than 0.1 V, as shown in Figure 4V. It is important to point out that the electrocatalytic behavior of the electrocatalyst is affected by the applied voltage.

**Figure 5:**
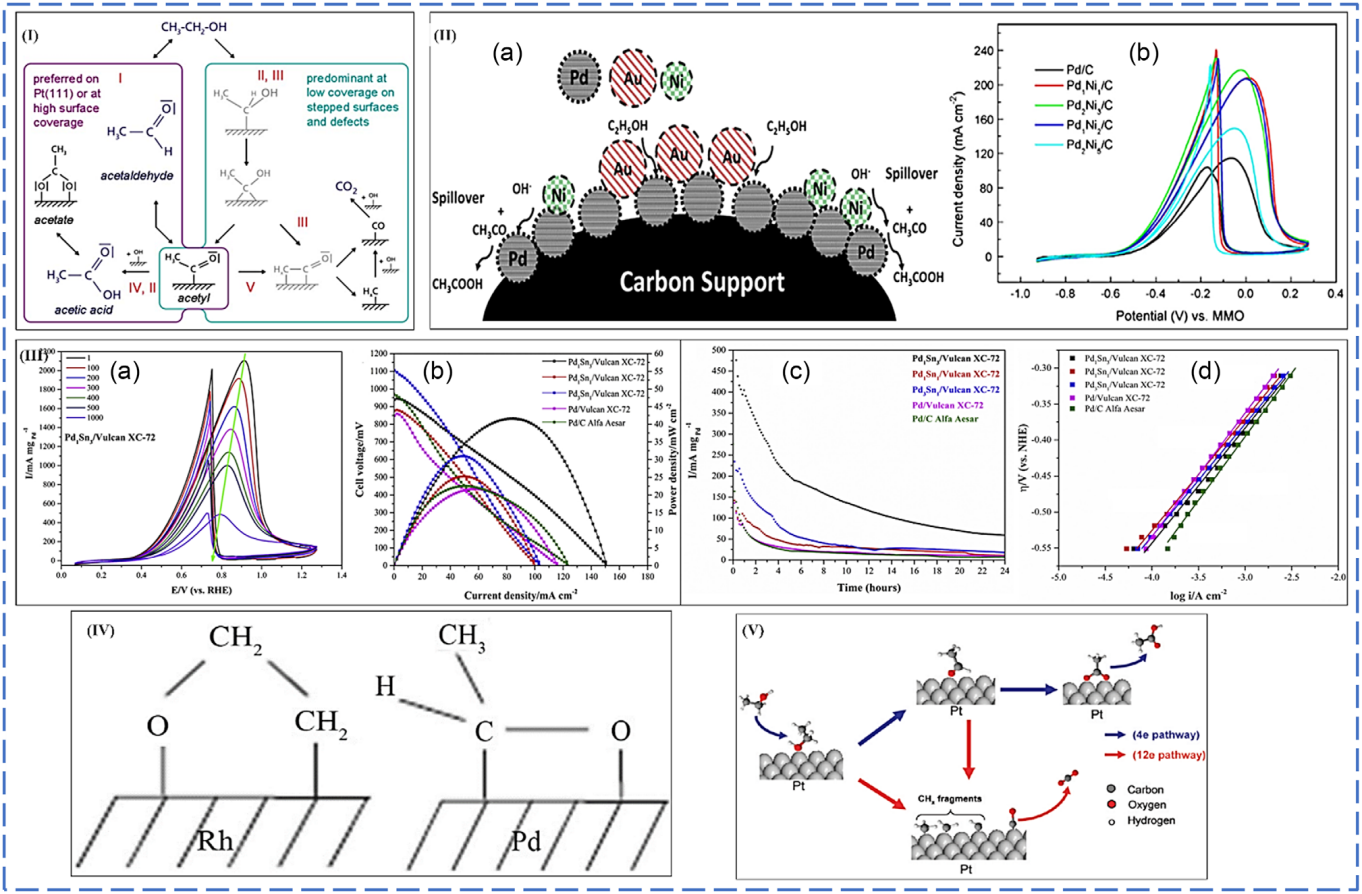
I: EOR mechanism on Pt. Right side (high surface coverage) and left side (low coverage).^[^ ^157^ ^]^ Reprinted with permission.^[^ ^37^ ^]^ Copyright 2010, American Chemical Society. II: a) A schematic diagram showing the effects of adatom high concentration: Pd catalytic site blockage. Reproduced with permission.^[^ ^177^ ^]^ Copyright 2013, Elsevier. b) CV curves of Pd‐based electrocatalysts at scan rate 50 mVs^−1^. Reproduced with permission.^[^ ^313^ ^]^ Copyright 2010, Elsevier. III: a) Cyclic stability test at a scan rate of 100 mV s^−1^. b) Polarization curves and density power versus density current at 80 °C. c) Chronoamperometry at 0.768 V versus RHE (–0.3 V vs SCE). d) Tafel of EOR plot from linear sweep voltammetry at a scan rate of 0.5 mV s^−1^ using 1.0 mol L^−1^ CH_3_CH_2_OH and 1.0 mol L^−1^ KOH. Reproduced with permission^[^ ^180^ ^]^ Copyright 2020, Elsevier. IV: CH_3_CH_2_OH adsorption mechanism on Rh and Pd surfaces, respectively. Reproduced with permission^[^ ^183^ ^]^ Copyright 2010, Elsevier. V: Schematic of EOR mechanistic pathways of ethanol electrochemical oxidation on Pt surfaces.^[^ ^314^ ^]^ Reprinted with permission.^[^ ^65^ ^]^ Copyright 2012, American Chemical Society.

In a bid to try to clear and align the differing EOR mechanistic pathways, Melke et al.^[^ ^156^, ^157^ ^]^ developed a schematic, **Figure** **5**I, for EOR, which is based on the findings from different research groups. Their results pointed out that the EOR pathway mainly depends on electrolytes' pH and electrocatalyst architectural designs. Figure 5I shows that the acetyl, CH_3_CHO, is the interlink intermediate governing the final pathway followed by byproducts. This will be governed by the electrocatalysts' ability to enhance the C—C bond cleavage or CH_3_COOH formation.^[^ ^158^ ^]^

From the literature survey done over the past three decades, palladium has emerged as the main competitor of Pt toward EOR, mainly in alkaline media. The electro‐oxidation activity of Pd‐based electrocatalysts has been studied based on the electrocatalysts' morphological orientation,^[^ ^24^, ^159^, ^160^ ^]^ support material used, for example, metal oxides^[^ ^161^ ^]^ and carbonaceous supports.^[^ ^162^, ^163^ ^]^ The support materials provide a large surface area for electrocatalyst dispersion, enhancing the exposure of catalytically active sites.

More so, the metal oxide support induces the promotional effect on the electrocatalyst, as reported by Monyoncho et al.^[^ ^161^ ^]^ and Xu et al.^[^ ^164^ ^]^ The EOR has proved to be more achievable on Pd than Pt‐based electrocatalysts.^[^ ^164^, ^165^, ^166^ ^]^ Sheng et al.^[^ ^167^ ^]^ used ab initio computation on Pd in alkaline conditions and showed the relevance of water‐adsorbed hydroxide species in the electrocatalytic enhancement of EOR. A study by Fang et al.^[^ ^168^ ^]^ used CV and in situ infrared spectroscopy, and they observed that the highest electrocatalytic performance was at pH = 14. They also pointed out that acetate was formed at low pH. In precedence, different palladium‐based electrocatalysts have been made to date. These include bimetallic and trimetallic electrocatalysts.

Due to the multistep nature and various intermediates formed in EOR, a single metal‐based electrocatalyst does not have sufficient electro‐oxidation properties to facilitate the whole chain reaction. Thus, Pd has been alloyed by adatoms. The alloying of the adatoms with Pd changes the bond between the two (interatomic distances because of altered lattice strains) to favor water disassociation and promote the bifunctional effect. The adatom enhances Pd d‐band splitting, thereby altering the electronic configuration and favoring weak adsorption of poisonous intermediate species, for example, COads, CHx, ads, etc. As a result of the bifunctional effect, these termed intermediates can be easily oxidized to CO_2_. The overall effect of Pd alloying with adatoms reduces the binding energy of the electrocatalysts with the intermediates. The alloying enhances the adsorption of OH^−^ at low potentials, thus improving the electrocatalyst's electrochemical properties. Therefore, the overall effect enhances electrocatalytic activity and high poison tolerance. However, for a comprehensive understanding of electrocatalysts’ electro‐oxidation structural kinetics, interlink behavior is of fundamental importance.

Following Li and Zhao, the use of Pd in the alkaline electrolyte provides a bigger room for a more comprehensive selection of metallic cocatalysts. Mostly, bimetallic electrocatalysts have been given much attention. Of interest is the Pd–Ni^[^ ^169^, ^170^, ^171^, ^172^ ^]^ owing to the low price of nickel. Typically, Pd‐based electrocatalysts are supported on carbon‐based materials, for example, multiwalled carbon nanotubes (MWCNTs), graphene, CNFs, etc., so as a mitigation strategy hindering their sintering and dissolution,^[^ ^173^ ^]^ as a result of high surface area, conductivity, and stability in alkaline media. Chen and co‐workers employed a modified polyol method to synthesize PdNi supported on MWCNTs. Their study alluded that Ni existed as Ni (OH)_2_ in the nanocomposite, where the hydroxide form of Ni was attributed to the improvement in poisoning resistance.^[^ ^174^ ^]^

In another study, Sheikh et al.^[^ ^175^ ^]^ prepared Pd–Ni/C electrocatalysts using an impregnation‐reduction route. Herein they reported high electrocatalytic activity on Pd_40_Ni_60_/C. They also attributed the increased activity to Ni hydroxides, Ni (OH)_*x*_. In an independent study, Qi et al.^[^ ^176^ ^]^ prepared Pd_40_Ni_60_ by dealloying Al_75_Pd_10_Ni_15_ using 20 wt% of sodium hydroxide. They also reported superior electrocatalytic activity in alkaline media. However, Dutta and Datta reported the synthesis of Pd_*x*_Ni_*y*_/C by the NaBH_4_ reduction method and coined the improvement of electrocatalytic activity to the presence of nickel oxide NiO. This prompted other researchers to use NiO as support, for example, Xu et al.^[^ ^164^ ^]^ synthesized Pd‐NiO, which has high electrocatalytic activity and steady stability for EOR. Still, not many details are available in the literature regarding the promotional effect of NiO on EOR. The most generalized idea of the importance of Ni usage lies in the fact that this adatom is a good attractor of OH^−^.^[^ ^177^, ^178^ ^]^

It is noteworthy to point out that high electrocatalyst architectural engineering designs are of paramount importance. The optimizations are essential because a high concentration of the adatoms results in the blockage of electrocatalytic activity sites, thus inherently reducing EOR, as shown in Figure 5II. As shown in Figure 5IIa, uncontrolled adatom addition can interfere with the Pd adsorption/desorption of the intermediate species. As Ni content increased, the activity of the composite electrocatalysts antagonistically decreased. Figure 5IIb shows that the highest peak occurred for Pd_1_Ni_1_. Thus, Ni increase corresponded to CV maximum peaks’ decrease.

Sn‐alloyed electrocatalysts have been reported. The usage of tin as an adatom to Pd has been due to its enhancement ability of Pd's electrochemically active surface area and the ease of OH^−^ adsorption–dissociation and thus, an improvement of catalytic activity. Computational studies by Du et al.^[^ ^179^ ^]^ revealed that Sn‐alloyed Pd exhibits lower reaction energy toward EOR dehydrogenation steps. Most recently, Pinheiro et al.^[^ ^180^ ^]^ synthesized a series of Sn Pd electrocatalysts. For the best‐performing electrocatalyst, they reported a high current density Figure 5IIIa, maximum current density (152 mA cm^−2^), and power density (42 mW cm^−2^) with an open‐circuit potential of 939 mV at 80 °C, Figure 5IIIb. Such a good performance was attributed to the oxophilic nature of Sn, which is said to improve CO_ads_ removal, the presence of vacancies, and defects in Pd_*x*_Sn_*y*_/Vulcan XC‐72 electrocatalysts. These attributes improved the intrinsic properties of the electrocatalyst. After 24 h of the chronoamperometric run, Figure 5IIIc, the synthesized electrocatalysts were stable. They also reported high *i*_0_ and low Tafel slopes, indicating a high electron transfer rate and lower energy for starting the EOR, as shown in Figure 5IIId. Herein, they based the synthesized electrocatalyst activity on the widely accepted mechanism (Equation ((26), (27), (28))–(29)):^[^ ^23^, ^139^ ^]^

(26) $$\text{Pd}+{\text{OH}}^{-}\to \text{Pd}–{\text{OH}}_{\text{ads}}+e^{-}$$

(27) $$\text{Pd}+{\text{CH}}_{3}{\text{CH}}_{2}\text{OH}+3{\text{OH}}^{-}\to \text{Pd}–{\left({\text{CH}}_{3}\text{CO}\right)}_{\text{ads}}+{\text{3H}}_{2}O+3e^{-}$$

(28) $$\text{Pd}–{\left({\text{CH}}_{3}\text{CO}\right)}_{\text{ads}}+{\text{Pd}-\text{OH}}_{\text{ads}}\to \text{Pd}+{\text{CH}}_{3}\text{COOH}+\text{Pd}$$

(29) $$\text{Pd}–{\text{CH}}_{3}\text{COOH}+{\text{OH}}^{-}\to \text{Pd}+{\text{CH}}_{3}{\text{COO}}^{-}+H_{2}O$$

The Ru‐alloyed Pd is the group of catalysts that proved to offer a better role in the EOR.^[^ ^23^, ^181^ ^]^ Ru selection has been attributed to its ability to split water molecules, thereby increasing the concentration of OH^−^. This is mainly due to their oxyphilic nature and intrinsic electrocatalytic activity. Monyoncho's group synthesized a series of Ru‐alloyed Pd electrocatalysts, Pd_*x*_Ru_*1–x*_/C (*x* = 1, 0.99, 0.95, 0.90, 0.80, 0.50).^[^ ^182^ ^]^ XRD showed the presence of metallic palladium, Ru, and Pd oxides. As alluded to before, the Pd/Ru oxides attract OH, facilitating EOR. This group reported that the addition of Ru lowered the onset potential of CV, resulting in improved current densities. The best‐performing electrocatalysts were Pd_90_Ru_10_/C and Pd_99_Ru_1_/C, which showed sixtime fold relative to the commercial Pd/C. Shen et al.^[^ ^183^ ^]^ studied EOR on the Pd–Rh electrocatalyst, and they reported different adsorption orientation configurations of the CH_3_CH_2_OH, as shown in Figure 5IV. Rh was said to adsorb using C1 and the oxygen atom, while Pd used C2 and the oxygen atom. From this, they concluded that the Rh facilitates CH_3_CH_2_OH to CO_2,_ and Pd forms acetaldehyde.

Some groups have also reported Pd‐based bimetallic Pd–Pb,^[^ ^184^ ^]^ Pd–In_2_O_3_,^[^ ^185^ ^]^ Pd–Cu,^[^ ^186^, ^187^, ^188^ ^]^ Pd–Au,^[^ ^189^, ^190^, ^191^ ^]^ Pd–Ti,^[^ ^192^ ^]^ Pd–Ag,^[^ ^193^, ^194^ ^]^ Pd–Ir,^[^ ^195^ ^]^ Pd–V,^[^ ^196^ ^]^ NiPdAu,^[^ ^197^ ^]^ Pd–Fe_2_CoO_*x*_,^[^ ^198^ ^]^ etc. Despite the greater strides and focus on Pd‐based electrocatalysts, the research on Pt‐based electrocatalysts has not ceased. However, monometallic Pt has been ruled out from the Pt‐based electrocatalysts because of their low efficiency and high poisoning rates. The hope of using Pt‐based electrocatalysts now lies in reducing the amount of Pt with the electrocatalyst composite (reducing cost) and using support materials for electrocatalyst dispersion. This will collectively improve and expose more electrocatalytic active sites for EOR and morphology–activity dependence (however, morphological activity dependency is highly elusive). To realize these alluded routes, there should be no compromise of electrocatalytic activity, cyclic stability, and durability. The EOR on the surface of platinum‐based electrocatalysts is shown in Figure 5V.

Research is still being done to determine the EOR reaction parameters and the associated mechanistic pathways. However, there are still some extreme mismatches in the literature. For example, Zhou et al.^[^ ^199^ ^]^ and Tian et al.^[^ ^200^ ^]^ ascribed the plausible electrocatalytic behavior of tetrahexahedral Pt nanocrystals to high densities of the stepped atoms. In contrast, Li et al.^[^ ^201^ ^]^ coined the improved electrocatalytic activity to the morphological architecture of the Pt (spherical and NWs), specified in their study as a well‐defined ensemble of the larger crystal facets and small density of the defective sites. This calls for a new view of the EOR electrocatalytic mechanism.

Many types of research have been aimed at transiting from monometallic to mainly bimetallic and trimetallic Pt‐based electrocatalysts. There have also been reports on non‐PGM‐based electrocatalysts for EOR; however, their activity evaluation is still in its infancy. The developed electrocatalysts have shown better performances as compared to standalone Pt. The alloying has managed to address the poisoning effects of CO_ads_ partially; however, the partial address has been satisfactory and shows a direction to some extent.

Of much focus have been Pt–Sn,^[^ ^202^, ^203^, ^204^, ^205^, ^206^, ^207^ ^]^ Pt–Ru,^[^ ^111^, ^161^, ^162^ ^]^ and Pt–Rh.^[^ ^141^, ^161^, ^163^ ^]^ Pt–Ru is the archaic electrocatalyst of all the bimetallic and most studied thereof. In 2008, Petrii wrote a well‐elaborated and comprehensive review of the research done on Pt–Ru; hence, readers are referred to the review.^[^ ^153^, ^208^, ^209^ ^]^ The review points out the fundamental results for EOR, which now lies as the research backbone for advanced Pt–Ru development. Petrii also showed the material architectural engineering relation to EOR electrocatalysis. This review^[^ ^153^ ^]^ is divided into 1) 1963–1970 prehistoric discoveries of Pt–Ru, 2) classification of Pt–Ru based on interlink between the structural morphology and electrocatalytic activity, and 3) advanced structural–mechanistic relations coupled to electrocatalytic enhancement via the oxidative routes.

However, in a glimpse, Ru as an ad–atom in Pt bimetallic acts as an oxygen attractor and thus a supplier of oxygen in EOR pathways. The literature has some disagreeing findings, especially on the Ru optimum percentage in the bimetallic.^[^ ^210^, ^211^ ^]^ For example, Spinace et al.^[^ ^212^ ^]^ and Camara et al.^[^ ^213^ ^]^ reported 30% and 40% optimum content of Ru in Pt–Ru. Another adatom that has developed interest among researchers is Sn. Artyushkova et al.^[^ ^186^ ^]^ clearly outlined the activity of the Pt–Sn electrocatalyst.

**Figure 6:**
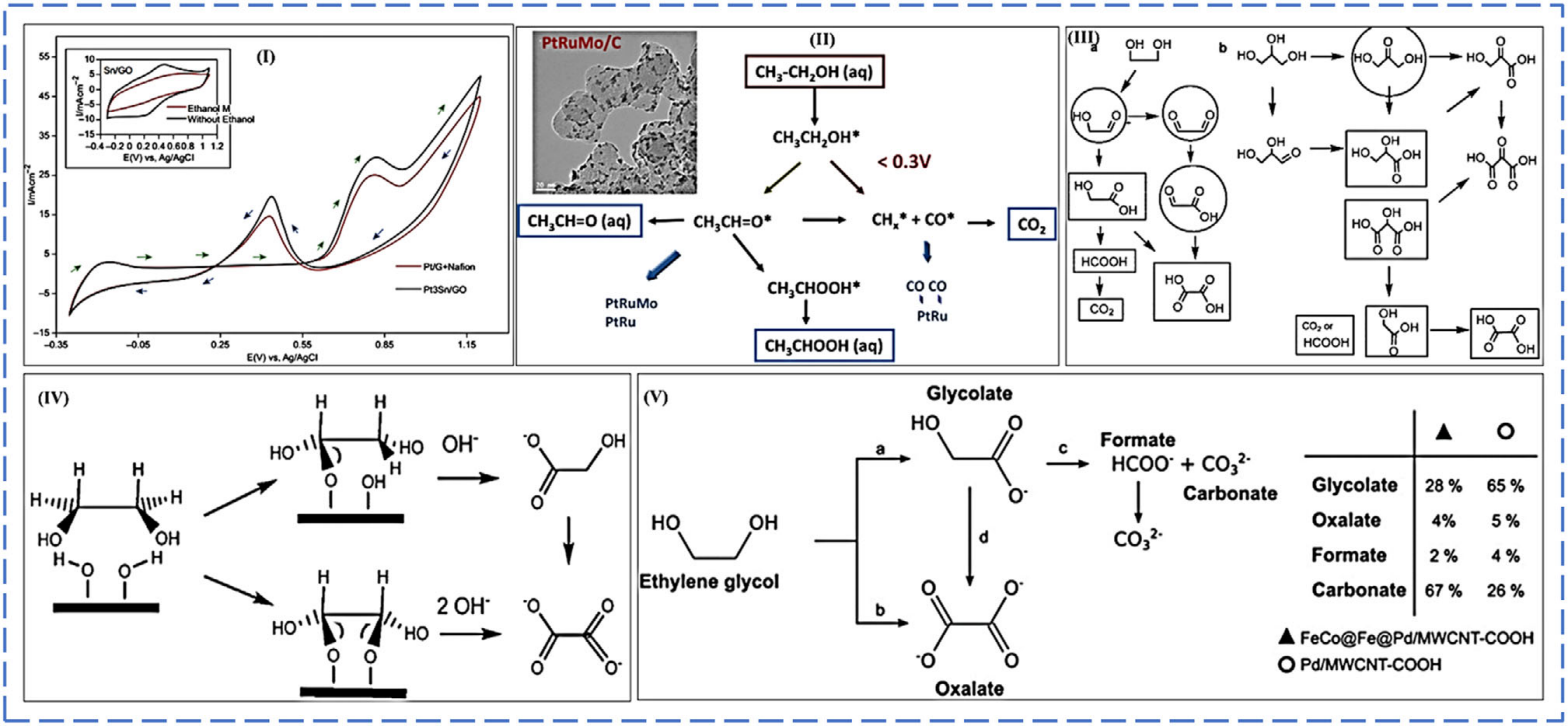
I) Cyclic voltammograms of Pt_3_Sn/(GO), Pt/G + Nafion, and Sn/GO electrodes at a Pt loading of 90 mg cm^−2^ in 1 Methanol and 0.5 mol L^−1^ H_2_SO_4_ at 50 mV s^−1^. Reproduced with permission^[^ ^214^ ^]^ Copyright 2015, Elsevier. II) Transmission electron microscope image PtRuMo/C catalyst and scheme representing a suggested mechanistic pathway of electrochemical oxidation of CH_3_CH_2_OH on PtRuMo/C electrocatalysts. Reproduced with permission^[^ ^315^ ^]^ Copyright 2017, Elsevier. III) Schematic illustration of metallic‐based oxidation of ethylene glycol (route a) and glycerol (route b).^[^ ^224^ ^]^ Reprinted with permission.^[^ ^91^ ^]^ Copyright 2009, American Chemical Society. IV) Oxidation paths of ethylene glycol to glycolate and oxalate.^[^ ^224^ ^]^ Reprinted with permission.^[^ ^91^ ^]^ Copyright 2009, American Chemical Society. V) Ethylene glycol electro‐oxidation on FeCo@Fe@Pd/MWCNT‐COOH and Pd/MWCNT‐COOH in alkaline medium. Reproduced with permission.^[^ ^230^ ^]^ Copyright 2015, Royal Society of Chemistry.

Interestingly, the Pt–Sn shows different electrochemical routes based on structural morphology. They alluded to metallic form‐based Pt–Sn electrocatalytic enhancement based on a well‐defined inner sphere electron transfer (ISET). At the same time, for EOR in an alkaline medium, there must be higher proportions of Pt (OH)_*x*_ and Sn(OH)_*x*_. This supports the postulations of the handiness of OH^−^ in the oxidation of alcohols. Thus, the presence of Pt/Sn hydroxides promotes the outer sphere electron transfer (OSET). The dispersion of Pt–Sn has also proved and shown favorable results for its consideration. The support material provides an enhanced anchor for the electrocatalysts and has let alone the intimate interaction and synergy; this dramatically reduces the noble metal content, ensuring the economic viability of the electrocatalysts. Kakaei synthesized Pt and Sn on graphene oxide (GO).^[^ ^214^ ^]^ Herein, Kakaei showed the promotional effect of Sn (Sn showed no effect on EOR), **Figure** **6**I, and proposed a reaction mechanism on Pt–Sn/GO.^[^ ^215^, ^216^ ^]^

(30) $$\text{RGO}+H_{2}O\to \text{RGO}–{\text{OH}}_{\text{ads}}+H^{+}+e^{-}$$

(31) $$\text{Pt}–{\text{CO}}_{\text{ads}}+\text{RGO}–{\text{OH}}_{\text{ads}}\to {\text{CO}}_{2}+H^{+}+\text{Pt}+\text{RGO}+e^{-}$$

(32) $$\text{Pt}–{\text{CO}}_{\text{ads}}+{\text{Sn}–\text{OH}}_{\text{ads}}\to {\text{CO}}_{2}+\text{Pt}+\text{Sn}+H^{+}+e^{-}$$

Equation (30) shows the importance of water molecules. The H_2_O is stripped of its H+, resulting in hydroxylated RGO and RGO–OHads, thus increasing OH– in the reaction system EOR. In Equation (31), the formed RGO–OH_ads_ then provides extra oxygen for further oxidation of adsorbed CO in Pt–CO_ads_, thereby forming free leaving CO_2_. The phenomenon occurs for the hydroxylated tin, Sn–OH_ads_, Equation (32), like RGO–OH_ads_, and provides an oxidative route for CO_ads_ to CO_2_. Thus, from this study, a conclusion can be drawn that OH^−^ plays a vital role in EOR mechanistic pathway in mitigating CO electrocatalyst poisoning.

Different groups of researchers have synthesized and applied trimetallic electrocatalysts in EOR. The development of trimetallic electrocatalysts requires an individualized understanding of the contributing adatoms and evaluation of the compatibility between trio‐metals.^[^ ^164^, ^167^ ^]^ For example, Sn enhances the electrocatalysts' poisoning tolerance, and Ru propels the dissociation of water molecules on the surface of the electrocatalyst. Another important point to note in trimetallic electrocatalysts is enhanced synergy, which helps mitigate the poisoning of electrocatalysts by weakening the bond between Pt and adsorbed CO.^[^ ^217^ ^]^ The trimetallic electrocatalysts for EOR synthesized to date are PtRuSn,^[^ ^218^ ^]^ PtRuNi,^[^ ^216^ ^]^ PtRhSn,^[^ ^201^ ^]^ PtMoIr,^[^ ^219^ ^]^ PtRuMo,^[^ ^220^ ^]^ or PtSnMo.^[^ ^221^ ^]^ Kowal et al.^[^ ^222^ ^]^ reported that in acidic conditions, the prepared PtRhSnO_2_/C electrocatalyst facilitated the C—C bond cleavage at low potentials. In this study, Pt was said to facilitate dehydrogenation. Rh weakly bound to CO, hence reducing catalytic poisoning. SnO_2_ helps water dissociation and hence the inherent provision of OH^−^ which facilitate the oxidation of the CO_ads_ to CO_2_. Wang et al.^[^ ^216^ ^]^ synthesized PtRuNi for application in EOR, and they pointed out the importance of Ni in the electrocatalytic EOR as a result of the enhanced formation OH_ads_. In another study, Ribadeneira et al.^[^ ^215^ ^]^ synthesized PtRuNi and PtSnNi. They concluded that PtRuNi was most electroactive than PtSnNi. This was attributed to the incompatibility of Sn alloying with Ni. They found that Sn is only much more compatible with Pt. Garcia et al.^[^ ^220^ ^]^ synthesized PtRuMo/C; they noted that an increase in Mo resulted in associated high current densities from high C2 products and not complete CH_3_CH_2_OH electro‐oxidation. The proposed mechanism is shown in Figure 6II.

#### 8.1.3 Electrocatalytic Oxidation of Polyhydric Alcohols

The higher alcohols ethylene glycol (EG)‐ and glycerol (Gly)‐based FCs have seen a rise in research due to their merits when used in FCs. Ethylene glycol is nonvolatile, has a specific energy of 8.6 kWh kg^−1^, is cheap, of renewable origin (obtained from cellulose), and is nontoxic. Glycerol is affordable, less poisonous, has high theoretical energy density of 6.4 KWh L^−1^, is nonflammable, and renewable (a byproduct of biodiesel production).^[^ ^26^, ^223^ ^]^ The electrochemical oxidation of these polyhydric alcohols is complex due to the complexity of their structures. Due to that, there is partial oxidation, which means that there are multiple routes via intermediates to the final product. Chemically this means that the scission of the C—C bond is very difficult. As a result, many intermediates are realized. For ethylene glycol, the intermediates mainly generated are glycolaldehyde, glyoxal, glycolic acid, glyoxylic acid, and oxalic acid.

In contrast, glycerol gives glyceric acid, tartronic acid, mesoxalic acid, and glycolic acid. Bianchini and Shen proposed a generalized EG and Gly oxidation on metal electrocatalysts, as shown in Figure 6III.^[^ ^224^ ^]^ With these valuable intermediates' products, the electrochemical conversion of polyhydric alcohols in FCs can be used in electricity cogeneration and the production of VAPs.^[^ ^225^ ^]^ Hence, the FC can be used as a reactor.^[^ ^173^, ^226^ ^]^

For the successful electrochemical conversion of the higher alcohols, alkaline conditions are much more favored.^[^ ^15^, ^227^ ^]^ However, the need for high alkaline conditions inhibits polyhydric‐based FCs' use in normal operational conditions, hence limiting their entire application. The main reason for that is the inherent poisoning of the electrolyte by carbon dioxide, be it from the air or the oxidation process. The poisoning of the electrolyte occurs as shown in Equation (33).
(33)

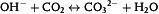




##### Ethylene Glycol Oxidation Reaction (EGOR)

The complete electro‐oxidation of EG via the C—C bond scission releases a maximum of ten electrons according to Equation (34).

(34) $${\text{HOCH}}_{2}{\text{CH}}_{2}\text{OH}+{\text{10OH}}^{-}\to 2{\text{CO}}_{2}+8H_{2}O+{10e}^{-}$$

However, in practice, there is only partial oxidation, primarily to oxalate species, Figure 6IV, where only eight electrons are released; the product formed is, however, dependent on pH.^[^ ^228^, ^229^ ^]^

The products and their respective amounts are dependent on the type and composition of the electrocatalyst used. In that light, Ozoemena et al.^[^ ^230^ ^]^ synthesized a core–shell–shell electrocatalyst FeCo@Fe@Pd/MWCNT‐COOH for the electrocatalysis of ethylene glycol. They reported the electrocatalyst to be highly selective toward carbonate formation. From the results they got, they proposed a mechanism for the catalytic activity of FeCo@Fe@Pd/MWCNT‐COOH toward ethylene glycol electro‐oxidation, as shown in Figure 6V. In their proposed mechanistic pathway, only one direct partway led to the formation of oxalate (route b). From a close look at the mechanism, it can be seen that route a allowed for the oxidation of only one hydroxyl group of the EG, which formed glycolate. From the formed glycolate, there will be further oxidation via two routes; in route c, the glycolate can be oxidized to formate and carbonate, where they will further dissociate to form more carbonate. Overall, route c produces more carbonate. Based on the amount of oxalate formed, route d is not favored. The formation of CO_3_^2−^ indicates that there was a C—C bond scission. Therefore, from route c, FeCo@Fe@Pd/MWCNT‐COOH and Pd/MWCNT‐COOH produced 67% and 26% carbonate, respectively, indicating that FeCo@Fe@Pd/MWCNT‐COOH is more robust in the cleavage of the C—C bond.

Miyazaki et al.^[^ ^231^ ^]^ reported the formation of glycolate and formate on Pt/C electrocatalyst. Just as found by Ozoemena et al.,^[^ ^230^ ^]^ Miyazaki said the scission of the C—C bond formed in the formate route formation, Equation (35), with the release of six electrons. Whereas for the glycolate route, the C—C bond remained unbroken in Equation (36), with the release of four electrons.
(35)

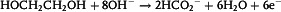

(36) $${\text{HOCH}}_{2}{\text{CH}}_{2}\text{OH}+{5\text{OH}}^{-}\to 2{\text{HOCH}}_{2}{\text{COO}}^{-}+4H_{2}O+4e^{-}$$

**Figure 7:**
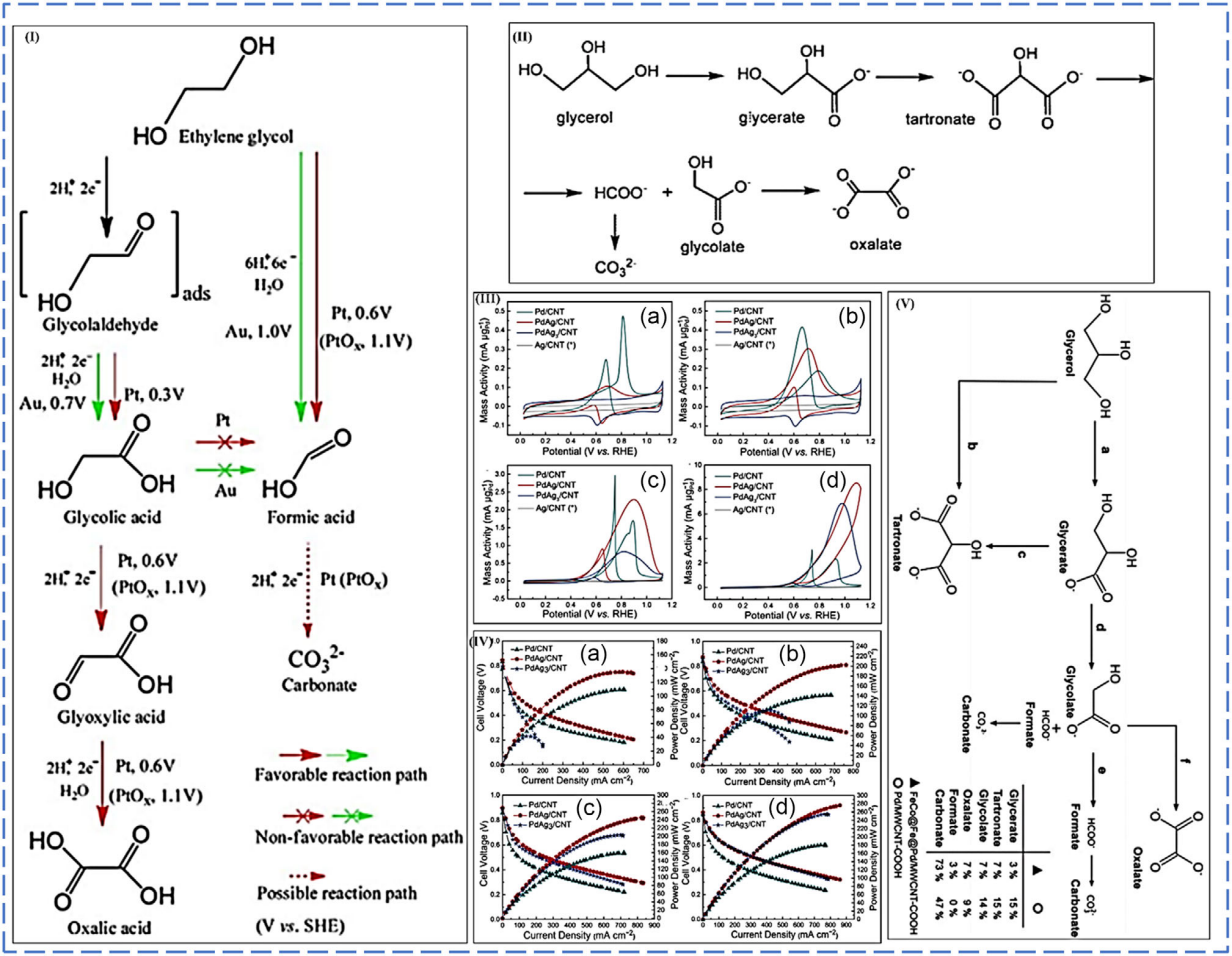
I) Reaction pathways for potential‐assisted electro‐oxidation of ethylene glycol on Au/C and Pt/C electrocatalysts in an alkaline medium. Reproduced with permission.^[^ ^232^ ^]^ Copyright 2012, Elsevier. II) Products of Pd/MWCNT anodes’ partial oxidation of glycerol. Reproduced with permission.^[^ ^316^ ^]^ Copyright 2009, Elsevier. III) Cyclic voltammograms of Pd/CNT, PdAg/CNT, PdAg_3_/CNT, and Ag/CNT for alcohol oxidation in N_2_‐purged 1.0 m KOH + 0.1 m: a) CH_3_OH, b) CH_3_CH_2_OH, c) HOCH_2_CH_2_OH, d) CH_2_OHCH(OH)CH_2_OH at 50 mV s^−1^. Reproduced with permission.^[^ ^234^ ^]^ Copyright 2016, Elsevier. IV) Cyclic voltammograms of Pd/CNT, PdAg/CNT, PdAg_3_/CNT, and Ag/CNT for alcohol oxidation in N_2_‐purged 1.0 m KOH + 0.1 m: a) CH_3_OH, b) CH_3_CH_2_OH, c) HOCH_2_CH_2_OH, d) CH_2_OHCH(OH)CH_2_OH at 50 mV s^−1^. Reproduced with permission.^[^ ^234^ ^]^ Copyright 2016, Elsevier. V) Glycerol electro‐oxidation on FeCo@Fe@Pd/MWCNT‐COOH and Pd/MWCNT‐COOH in alkaline medium. Reproduced with permission.^[^ ^230^ ^]^ Copyright 2015, Royal Society of Chemistry.

In another detailed study, Xin et al.^[^ ^232^ ^]^ reported the electro‐oxidation of ethylene glycol on supported Pt and Au. They reported the formation of glycolic acid, oxalic acid, and formic acid. They noted a decrease in the amount of acids formed with time, which they ascribed to metal oxide formation on the surface of the electrocatalyst. These oxides reduced the number of electrocatalytically active sites. In the mechanistic pathway based on their findings, **Figure** **7**I, there are two notable EG electro‐oxidation routes.

The direct formic acid route is a highly oxidative route that results in further oxidation to carbonates. The route proceeds by the scission of the C—C bond. The other route keeps C—C unbroken as the products in that reaction line are C2 based. A study by Coutanceau et al.^[^ ^228^ ^]^ highlighted bismuth's role in the electrocatalysts they synthesized (PtPd, PtBi, and PtPdBi). Bismuth was said to attract and enhance OH_ads_. Just like other researchers, they reported a mixture of products being formed during EG electro‐oxidation. Herein, they also noted that Pd addition to PtBi resulted in increased current densities. They attributed Pd to reduced poisoning effects of the electrocatalyst by its ability to favor hindrance of carbon‐to‐carbon scission through the formation of oxalate. The reported products included glycolic, oxalic, and formic acids. Yang et al.^[^ ^233^ ^]^ also reported high electrocatalytic electro‐oxidation of EG on Pd–Ag/C (4.85 times higher relative to commercial Pd/C).

##### Glycerol Oxidation Reaction (GOR)

Complete electro‐oxidation of glycerol liberates 14 electrons, as shown in Equation (37).
(37)

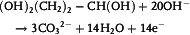




The scission of the C–C in glycerol during electrocatalytic electro‐oxidation proceeds via multisteps, releasing a smaller number of electrons than the theoretically postulated number. This means that just like EG electro‐oxidation, glycerol also produces intermediates, as shown in Figure 7II. The commonly realized intermediates are glyceric acid, tartronic acid, glycolic acid, etc. One of the attractive intermediates is formic acid which is employable in direct formic FCs.

Qi et al.^[^ ^234^ ^]^ synthesized PdAg/CNT for the electro‐oxidation of MetOH, EtOH, EG, and Gly. They used a half‐cell system in evaluating the electrocatalytic activity of the synthesized electrocatalyst. They reported that the electro‐oxidation of Gly had the highest peak mass activity of 8.53 mA μg_Pd_^−1^. Figure 7IIIa–d shows a peak power density of 276.2 mW cm^−2^ (Figure 7Iva–d) and corresponding peak mass activity of 552.4 mW mg_PdperMEA_^−1^. Ag was said to have a promotional effect toward aldehydes' further oxidation, enhancing glycerol oxidation on PdAg/CNT. Thus, the facilitation of C–C scission to C3 glycerol and C2 oxalate occurs.

Other groups have done research pointing out the roles of different electrocatalysts, for example, 20%, 30%, 40%, and 60% Pt on carbon,^[^ ^235^ ^]^ NiPi/Pi–Fe_2_O_3_,^[^ ^236^ ^]^ PdAg and PdAu,^[^ ^237^ ^]^ Pd nanodendrites,^[^ ^238^ ^]^ AgAu,^[^ ^239^ ^]^ etc. Interestingly, we note in the literature the study by Ozoemena et al.,^[^ ^230^ ^]^ where they synthesized and compared the electrocatalytic activity of FeCo@Fe@Pd/MWCNT‐COOH and Pd/MWCNT‐COOH toward glycerol electro‐oxidation. They proposed a reaction pathway based on the results they obtained, as shown in Figure 7V.

As alluded to earlier sections, the multistep nature of the glycerol electro‐oxidation can be seen from the routes a–f. Route b, the formation of tartronate, is the only direct route for the oxidation of glycerol. Tartronate can also be formed by further oxidation of glycerate in route c. Glycerate is first formed by the oxidation of one hydroxyl on glycerol (route b). The formation of tartronate requires further oxidation of the other terminal hydroxyl group. Route d represents the formation of glycolate and formate through the cleavage of the C—C bond. The formed glycolate can then be oxidized to oxalate (route f) of another C—C bond scission forming carbonate (route e). From the electro‐oxidation production quantification, the FeCo@Fe@Pd/MWCNT‐COOH gave 73% of carbonates relative to 47% of Pd/MWCNT‐COOH, as shown in Figure 7V. This indicates that FeCo@Fe@Pd/MWCNT‐COOH is a stronger electrocatalyst for the scission of the C—C bond. Compared to other literature reports, core–shell–shell structures are good candidates for selective and plausible catalytic electro‐oxidation of glycerol.

### 8.2 Oxygen Reduction Reaction (ORR)

The development of FCs with commercial realization is centered mainly on the enhancement of the ORR. This cathodic reaction is sluggish as it occurs with very slow kinetics. The inherently poor kinetics are dependent on the electrolytes' pH and reaction system temperature and the partial pressure of oxygen.^[^ ^240^ ^]^ As outlined by Wroblowa et al.,^[^ ^241^ ^]^ ORR occurs via direct or indirect pathways; the direct pathway is termed the four‐electron pathway, which is the most favored route. This direct pathway results in the production of water in acidic conditions and hydroxide species in alkaline conditions. The indirect pathway is termed the two‐electron route. This pathway results in the production of hydrogen peroxide, H_2_O_2_, and OOH* as ORR intermediate and hence not a favored pathway. The demerit is because it causes a reduction in current efficiencies and components dilapidation in FC and hence unsteady performances. The four‐electron pathway occurs via the dissociative mechanism. In the dissociative mechanism, there is an O—O bond scission, then the atomized O will be hydrogenated to OH and water, H_2_O. The two‐electron pathway occurs via the associative pathway. In the associative pathway, there is adsorption of oxygen, coupled with direct protonation (transfer of H^+^) electron transfer to OOH species. These two mechanisms can occur in both acidic and alkaline mediums and are represented by Equation (37)–(42).

#### 8.2.1 Oxygen Reduction Reaction in Alkaline Medium



(38)

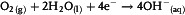



(39)

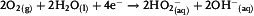



(40)

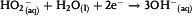




#### 8.2.2 Oxygen Reduction Reaction in an Acidic Medium



(41)

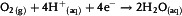



(42)

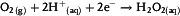



(43)

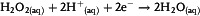




The reaction kinetics of the cathode ORR is extremely slow, limiting the large‐scale commercialization for FCs. As a result, exploring new effective electrocatalysts for breaking the O=O bond is extremely desirable. The lack of a suitably effective ORR electrocatalyst greatly affects the net efficiency of low‐temperature polymer electrolyte membrane (PEM) FCs. Although platinum NPs are still exhibiting high catalytic activity toward ORR over many other precious and nonprecious metal NPs, their low durability and abundance on Earth remain a significant hindrance to broad use in FCs. To address these concerns, alloying or incorporating Pt with other metals mainly cheap metals of 3*d* is a proven method for improving ORR performance and activity while simultaneously reducing Pt usage. Incorporation of other metals on Pt can also modify the d‐band structure of platinum, therefore increasing electrocatalytic activity and performance of Pt toward ORR by lowering the degree of adsorption of oxygenated intermediates.^[^ ^242^, ^243^, ^244^ ^]^ Deng et al.^[^ ^243^ ^]^ investigated an application of 1D mesoporous trimetallic AgPtPd nanotube composite as an effective electrocatalyst to boost the sluggishness of the ORR. The generated AgPdPt nanotubes composite exhibited significantly enhanced electrocatalytic activity with the best performance toward the ORR due to their mesoporous nanotube nature and trimetallic characteristics. Zhang et al.^[^ ^245^ ^]^ also studied the effect of trimetallic PtCoNi alloy nanoclusters composed of CoNi core and stable Pt shell to understand its synergistic contribution toward ORR. It was revealed that the trimetallic electrocatalyst PtCoNi/C exhibited much higher electrocatalytic activity and electrochemical stability than the bimetallic electrocatalytic system and pure Pt/C electrocatalyst.

Chang et al.^[^ ^246^ ^]^ also studied the catalytic activity of PtPd alloy NWs with strain‐modulated morphology toward ORR. Because of the composition‐dependent atomic‐scale alloying and faceting features, an electrocatalyst with the morphology of NWs showed better electrocatalytic activity and stability toward ORR than NP electrocatalysts with identical compositions. Xhao et al.^[^ ^247^ ^]^ also investigated an application of rhombohedral‐ordered intermetallic electrocatalyst to enhance ORR activity. The N‐doped and rhombohedral‐ordered intermetallic PtCuN/KB electrocatalyst with enhanced catalytic activity and durability for the ORR was successfully prepared. When compared to commercial Pt/C, the mass activity and surface area of rhombohedral‐ordered intermetallic PtCuN/KB electrocatalysts toward ORR improved by 5.2 and 3.9 times than that of pure commercial Pt/C electrocatalyst and even after 20 000 accelerated durability test cycles, the percentage loss in mass activity was found to be 23.4%.

### 8.3 Aspects Distinguishing ORR in Alkaline and Acidic Conditions

**Figure 8:**
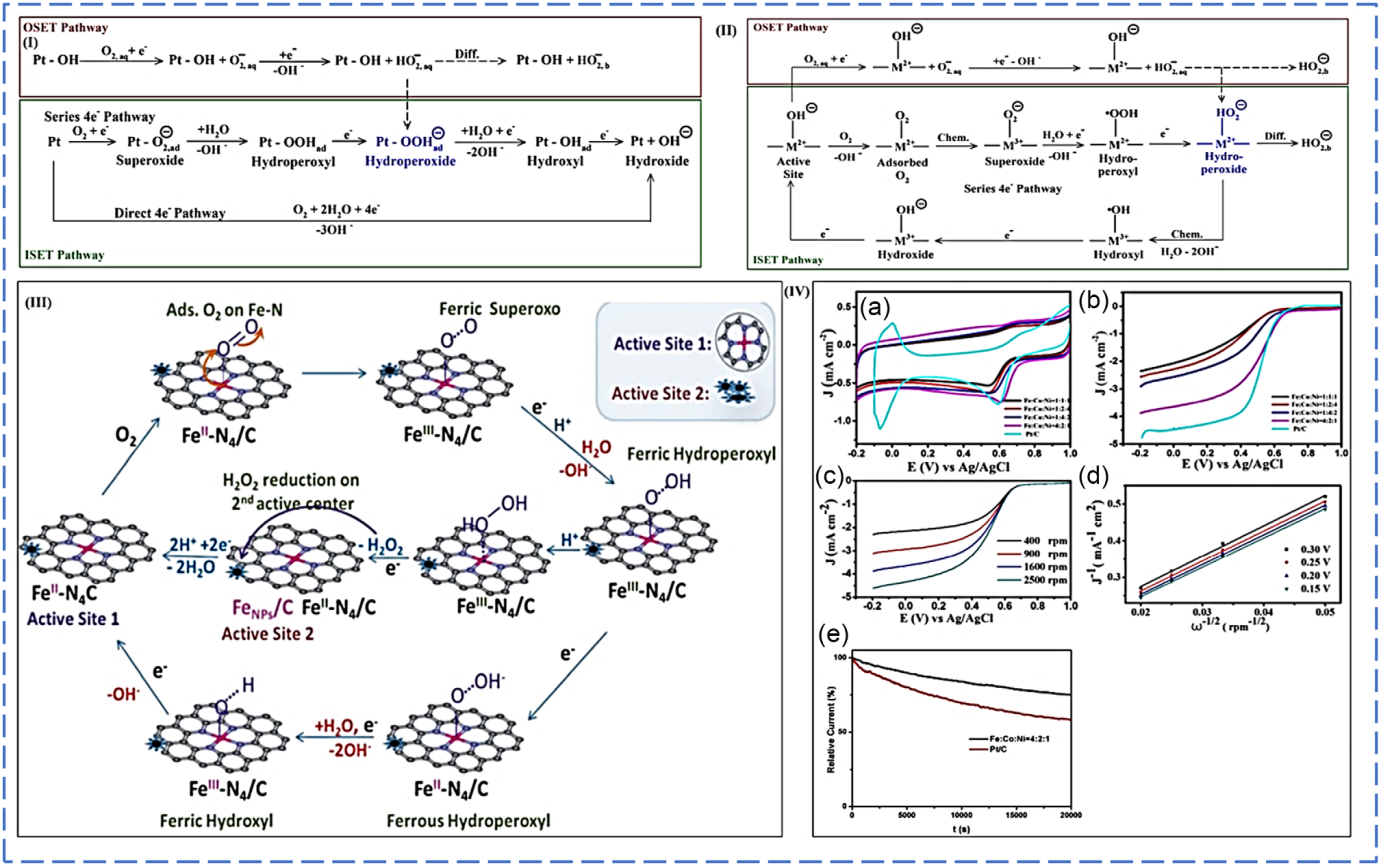
I: PGM surface aiding alkaline ORR pathways (top) and II: non‐PGM active sites. Pt and Pd represent PGMs surfaces, whereas the pyrolyzed Fe–N*x*–C, perovskites, and MnO*x* represent non‐PGMs active sites. For the non‐PGM catalysts, M2^+^ represents the active site. Subscripts aq, b, and ad represent aqueous, bulk, and adsorbed species and “Chem.” Is the chemical reaction step, and “Diff” is diffusion to bulk. III: Proposed ORR mechanistic pathways on Fe–N4/C and Fe NPs on carbon (in acidic and alkaline electrolytes).^[^ ^250^ ^]^ Reprinted with permission.^[^ ^102^ ^]^ Copyright 2014, American Chemical Society. IV: a) CV and b) RDE curves of FeCoNi‐N/CNF electrocatalysts with different Fe/Co/Ni ratios and 20% Pt/C catalyst. c) RDE curves at different rotation rates from 400 to 2500 rpm and d) corresponding Koutecky–Levich plots of FeCoNi‐N/CNF catalyst with a Fe/Co/Ni ratio of 4:2:1 in O_2_‐saturated 0.5 m H_2_SO_4_. e) Current–time i–t) chronoamperometric responses of the FeCoNi‐N/CNF catalyst with Fe/Co/Ni ratio of 4:2:1 and commercial 20% Pt/C catalyst in O_2_‐saturated 0.5 m H_2_SO_4_. Reproduced with Permission.^[^ ^254^ ^]^ Copyright 2012, Elsevier.

Two edge‐cutting aspects distinguish the ORR in alkaline and acidic mediums. The aspects are the nature of the first electron transfer step to molecular oxygen and base catalysis of the H_2_O_2_ intermediate. There is a limited choice of electrocatalyst in the acidic electrolyte. The feasibility is only of the PGMs as they have the optimal d‐electronic band structure coupled to favorable geometric orientation. They possess sufficient high free energy of adsorption for molecular oxygen and related oxygen moieties intermediates. However, for alkaline conditions, there is the possibility of the electrocatalytic ISET mechanism and the OSET. For the ISET, the molecular O_2_ directly chemisorbs dissociatively or associatively on oxide‐free platinum site^[^ ^248^ ^]^ catalytic pathway as there is optimal binding energy for oxygen adsorption. The ISET enables the 4e^-^ On the other hand, the OSET the solvated molecular oxygen $\left(O_{2}\cdot {\left(H_{2}O\right)}_{n}\right)$ undergoes direct electron transfer without direct chemisorption on the electrocatalytic active sites,^[^ ^249^ ^]^ also referred to as a noncatalytic pathway. Here, there is weak adsorption or adsorption of oxygen. The OSET follows the 2 e^−^ pathway which favors the formation of H_2_O_2_, the interaction of $O_{2}\cdot {\left(H_{2}O\right)}_{n}$ and OH_ads_ causing nonspecificity. The nonspecificity opens room for a wide range of non‐PGMs and non‐PGMs oxides as electrode electrocatalysts for the cathode side. In **Figure** **8**I,II, the schemes display the separate routes for metallic PGMs and non‐PGM surfaces, respectively.

A study has tried to explain the occurrence of ORR in both acidic and alkaline mediums on Fe–N4/C and FeNPS/C, as shown in Figure 8III.^[^ ^250^ ^]^ In their study, for the alkaline medium, they pointed out that the Fe–N4 centers are responsible for reducing the HO_2_^-^ species while the same centers coupled with the Fe NPs were said to be responsible for reducing the peroxide H_2_O_2_ only in an acidic medium. However, it is postulated that ORR is most pronounced and carries more merits in an alkaline medium. The reason being in an alkaline medium ORR is favored both thermodynamically and kinetically, the less corrosive nature of the electrolyte protects the cell components and necessitates the usage of non‐PGMs. The use of non‐PGMs brings a mitigation way to methanol cross‐over as most non‐PGMs are insensitive to methanol electrocatalysis.^[^ ^16^ ^]^

PGM‐free based electrocatalysts have received much attention. The transition metals that have been explored extensively include Mn, Ni, Fe, and Co, for the *M*‐N‐C electrocatalysts. Jaouen's et al.^[^ ^251^ ^]^ synthesized Fe/N/C and Co/N/C^[^ ^252^ ^]^ and reported that FeN4/C and N–FeN2 + 2/C were the most electroactive sites toward ORR. This same group has also studied Co–N–C. Conclusively, the Co‐based bound molecular oxygen weakly as compared to Fe‐based. Rojas‐Carbonell et al.^[^ ^253^ ^]^ synthesized Mn‐, Fe‐, Co‐, and Ni ‐ AAPyr (aminoantipyrine = AAPyr). They reported that Fe‐AAPyr showed very plausible ORR electroreduction activity and followed a four‐electron pathway.

Interestingly, Liu et al.^[^ ^254^ ^]^ synthesized FeCoNi–N/C using both electrospinning and impregnation methods. They reported that an optimized ratio of 4:2:1 for Fe: Co: Ni showed very high electroreduction properties owing to pyridinic‐N, quaternary‐N, and Fe_2_N in the nanocomposite. The electrocatalytic performance of FeCoNi–N/C was evaluated in acidic conditions using both CV and RDE experiments. From cyclic voltammograms in Figure 8IIIa, it can be noted that 4:2:1 has 0.74/0.61 V, 0.56 mA cm^−2^ with an onset potential of 0.73 V from RDE. Figure 8IIIb,c, which is comparable to 0.75 V of Pt/C, shows the activity of FeCoNi–N/C almost at par with the commercial Pt/C. From chronoamperometry studies, it was reported that FeCoNi–N/C (4:2:1) follows the four‐electron pathway, Figure 8IIId, and has good stability, Figure 8IIIe.

Carbonaceous heteroatom‐doped electrocatalysts have been studied for the ORR. The most investigated heteroatoms are nitrogen (N), sulfur (S), boron (B), and phosphorous (P). Of these, nitrogen has been studied extensively by Gong et al.^[^ ^255^ ^]^ This is mainly because it exhibits different configurations (pyridinic N, pyrrolic N, graphitic N, and oxidized pyridinic N). Pyridinic N and pyrrolic N have differing electrocatalytic activity contributions toward ORR.^[^ ^256^ ^]^ The effects of the dopants lie mainly in their electronegativities. For example, N is more electronegative than C; hence, it pulls the electron cloud toward itself, resulting in a partially positive C. While, for example, B has less electronegativity than C, it pushes the electron cloud toward C, creating a partial negative charge on C and hence a partially positive B. Both charge orientations enhance the interaction with molecular O_2_ during the electroreduction of the oxidant. Kumaresan et al.^[^ ^257^ ^]^ studied nitrogen‐doped porous carbon; the carbon was activated by lithium ion (Li ion), potassium ion (K ion), and calcium ion (Ca‐ion). They reported the Li‐ion‐activated N‐doped porous carbon had the greatest electroreduction of O_2_ due to high surface energy (73.2 mJ m^−2^) and Brunauer–Emmett–Teller surface area (824.02 m^2^ g^−1^), following a four‐electron route.

A series of heteroatom codoped carbons have been done. Yang et al.^[^ ^258^ ^]^ reported N, S@C_M_‐1000 to be active toward ORR. N, S@C_M_‐1000 was said to have N (4.1%), S (0.94%), specific area (280.1 m^2^ g^−1^), and *J*_L_ (5.5 mA cm^−2^) that showed capabilities of the possibility of being used a cathode electrocatalyst following the four‐electron pathway. In a different study by Xiong et al.,^[^ ^259^ ^]^ they synthesized NS‐HMCS‐32 and reported it to have N (4.8%) and S (1.4%), the specific surface area of (898 m^2^g^−1^), and *J*_L_ (5.1 mA cm^−2^). From the studies, the amount of dopant and the carbon type can influence electrocatalytic activity. Also, the dual doping of carbons presents electrocatalysts with a well‐defined role in the electroreduction of oxygen via the four‐electron route. It is worth pointing out that there is still the need for strategies for increasing the amounts of dopants on or within the carbon framework.

The most interesting electrocatalyst presenting an outstanding role is the core–shell architecture. The core–shell gives room for electrocatalysts engineering to suit its intended role and application. Thus, there can be optimizations concerning core and shell interaction, leading to well‐pronounced and well‐defined ORR electroactivity.^[^ ^260^ ^]^ In the core–shell structures, some properties of bimetallic, interfacial shell–core interaction can undoubtedly result in geometric and electronic modifications of the atoms constituting the shell. Huang et al.^[^ ^261^ ^]^ synthesized PdCu@Pd core–shell nanobranches using a simple one‐pot synthesis of H_2_ gas and Br^-^ ions as codirectors.

This synthesis route was reported to have given highly branched and defective PdCu@Pd. The PdCu@Pd electrocatalyst was reported to have enhanced electrocatalytic electroreduction kinetics (it directed a four‐electron transfer route). RRDE‐confirmed PdCu@Pd was to have produced less than 5% of hydrogen peroxide and superior stability and durability as proved by the methanol test. That is, PdCu@Pd showed antimethanol properties and mitigation for methanol cross‐over in FCs. In another study, Yusuf et al.^[^ ^262^ ^]^ fabricated a nanocomposite with a core–shell architecture, the palladium NP (Pd NP)‐loaded a‐Fe_2_O_3_@SiO_2_ core–shell nanospindle (PdFSCSNS). Their study reported that this electrocatalyst enhanced the ORR via the four‐electron pathway and insignificant amounts of H_2_O_2_, showing high stability and durability. Like the PdCu@Pd,^[^ ^261^ ^]^ PdFS‐CSNS showed antimethanol properties. Other ORR studies have been done on FeCo@Fe@Pd/C, Co_3_Fe_7_@Fe_2_N/rGO,^[^ ^263^ ^]^ Ag@Pd,^[^ ^264^ ^]^ Pt‐PdCo@Pd/C,^[^ ^265^ ^]^ etc.

From these studies, the uniqueness of the core–shell architecture is in the sense that they direct a four‐electron oxygen electroreduction and show antimethanol poisoning. These merits complement the reported core–shell structures' very high stability and durability. Hence, the outlined electrocatalysts can significantly enhance ORR and hence full FC commercialization.

## 9 Evaluation Parameters for ORR and AOR

### 9.1 Testing Protocols for Benchmarking ORR Activities in FC Electrocatalysis

The rotating disk electrode (RDE) method is a cost‐effective and widely used strategy for scanning the ORR activity of new materials. It employs the dispersion of the catalyst sample by homogenization with water, alcohol (ethanol or isopropyl in some cases), and Nafion by an optimal ratio, which is then deposited on a glassy carbon RDE. Sample deposition is usually regulated around 10 mg cm^−2^ to prevent catalyst agglomeration. After sample preparation, CV is performed in solutions of 0.1 KOH, 0.5 m H_2_SO_4_, or 0.1 m HClO_4_ saturated with an inert gas (Ar or N_2_) reduced and absorbed on the catalytic site for oxygen reduction which corresponds to the H^+^ adsorption on the current–potential (*I–V*) curve on the CV. In reverse, the regeneration of H^+^ occurs during anodic scanning and is then portrayed on the CV curve and corresponds to the desorption of these H^+^ atoms on the catalytic active site of the material.^[^ ^266^ ^]^

ORR polarization is obtained in a potential scanning window that ranges from 0.05 to 1.20 V with respect to the RHE utilizing an O_2_‐saturated solution of 0.1 KOH, 0.5 m H_2_SO_4_, or 0.1 m HClO_4._ To mitigate the effect of mass transfer on the electrocatalytic performance during ORR evaluation, the RDE is usually rotated at a speed of 1600 rpm to prevent mass transfer loss. To minimize the capacitive behavior and capacitive current of FCs under oxygen‐saturated solutions, the scan rate is usually controlled to be around 20 mV s^−1^. The Levich–Koutechy = Equation (43) shown below is used to obtain the kinetic current (*j*_k_) which is the catalytic current without the loss caused by mass transfer

**Figure 9:**
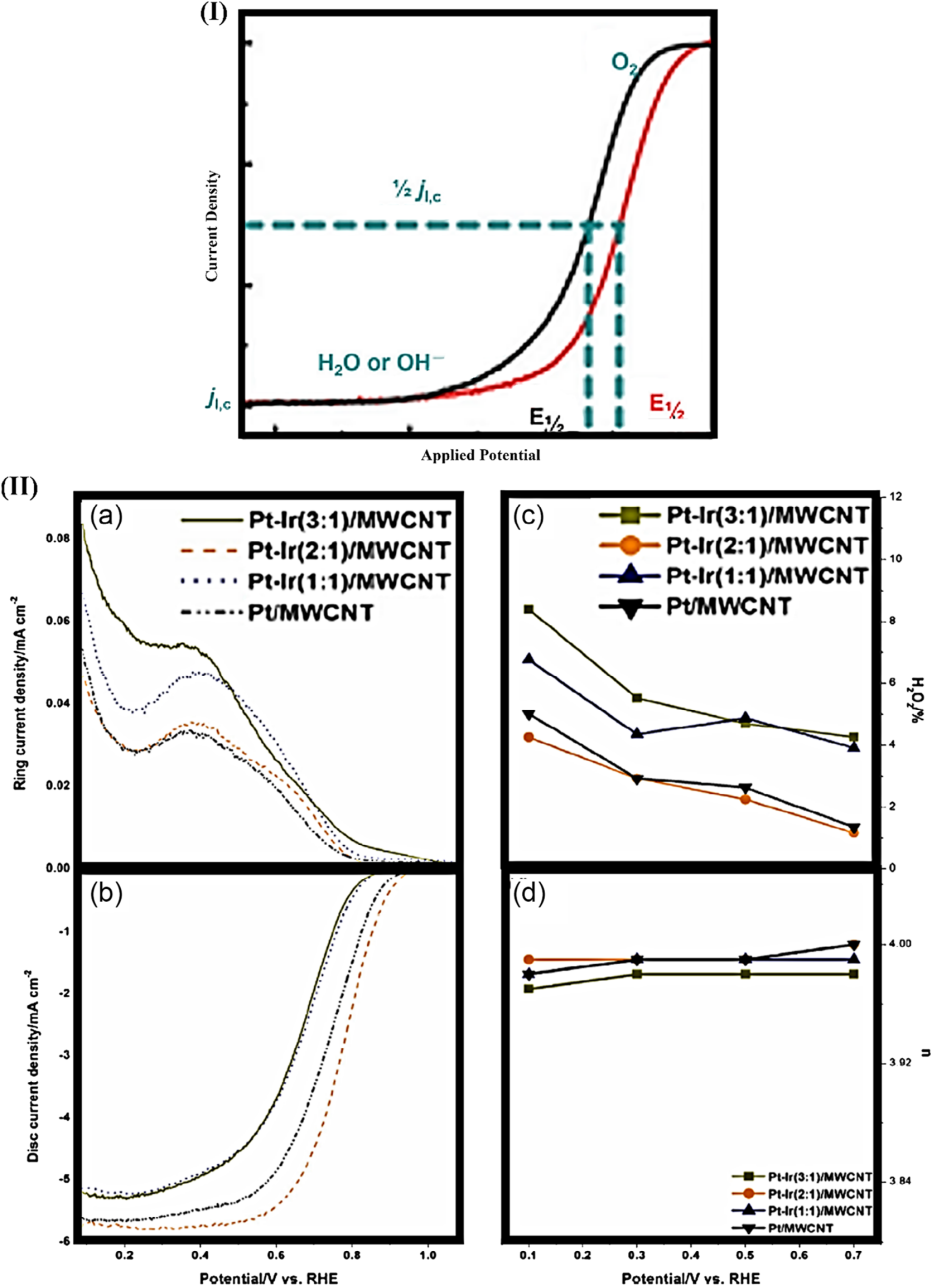
I) Polarization curve for ORR and the quantifying parameters used to evaluate the activity of the catalyst. Reproduced with permission.^[^ ^94^ ^]^ Royal Society of Chemistry II: a) disc and b) ring polarization curves for Pt‐Ir/MWCNT bimetallic composites and monometallic Pt/MWCNT catalysts recorded in N_2_‐saturated 0.1M HClO_4_ electrolyte solution at 0.1 V s^−1^ and trend graphs of c) the percentage of H_2_O_2_. d) The number of electrons transferred during ORR as a function of applied potential. Reproduced with permission.^[^ ^317^ ^]^ copyright 2020, Elsevier.

(44) 1j=1jk+1jl,c=1jk+10.62nFAC0*D02/3v−1/6ω1/2

where *j* is the current density which is extrapolated from the polarization curve under different applied potentials, as shown in **Figure** **9**I, and *j*_l,c_ is the diffusion‐limiting current density which is the highest current density under relatively negative potentials. By average the number of electrons transferred during ORR (*n*), Faradaic constant (*F*), the area of an electrode (*A*), diffusion coefficient (*D*_0_), the rotating speed of the RDE (*ω*), the kinetic viscosity of the solution (*v*), and the concentration of the O_2_ atoms dissolved in the catalysis solution (*C*_0_*), the diffusion‐limited current density (*j*_l,c_) can be obtained. A series of polarization curves under different rotation speeds of *j*_l,c_ facilitates the extraction of the kinetic current (*j*_k_) under 0.9 V with respect to the RHE. The extracted *j*_k_ can then be further conversed into a specific activity to obtain the mass activity and by normalizing with Pt (or other metal) loading and the electrochemical active surface area (ECSA).^[^ ^94^, ^266^ ^]^

The half‐wave potential (*E*_1/2_) and *j*_l,c_ are two significant parameters that are used to evaluate the catalytic capability of a material. As shown in Figure 9I, a half‐wave potential (*E*_1/2_) is the potential required to achieve the current that is half that of the *j*_l,c_ and this is also widely used to analyze the catalytic performance (mainly it is the midpoint of obtained LSV curve at 1600 rpm). A more positive *E*_1/2_ means that lower overpotential is required to achieve ½ *j*_l,c_ which then signifies that the material has a higher catalytic activity. Thus generally, optimal half‐wave potential, *E*_1/2_, is indicative of the thermodynamic, kinetic characteristics, and the ultimate efficiency of ORR with very minimal voltage losses. Mass activity as mentioned before is also used as a benchmark to analyze Pt‐based electrocatalysts for their catalytic activity. The United States Department of Energy (DOE) has set a target for the mass activity of 0.44 A mg_pt_^−1^ (for Pt group metals) at 0.900 V in a PEMFC which should be used as a benchmark for catalytic evaluation purposes.^[^ ^94^ ^]^ Thus, we conclude that from a catalytic activity point of view, *j* at 0.9 V is more relevant than *j*_l,c_ as it is closely linked to optimal and a very thin line with *E*_1/2_.

A rotating ring disk electrode (RRDE) made up of a glassy carbon (ring disk) is used to study the influence of kinetics and mass transfer effects on ORR by evaluating hydrodynamic conditions. The central disk of a RRDE is covered by a concentric ring electrode in a modified shape of the RDE and the gap between the disk and the ring electrode is filled by an insulator such as Teflon or epoxy resin. RRDE can be used to calculate rate constants faster than any other method which qualifies them as an efficient tool for studying steady‐state measurements. Compared to the RDE, the RRDE produces two types of current which are the disk current (*I*_D_) and ring current (*I*_R_) respectively. The ring current refers to how much of the analyte material is returned to the starting material while the disk current refers to how much of the analyte is produced.^[^ ^267^ ^]^

In ORR studies, the formation of intermediate species directly impacts the reaction kinetics and plays a crucial role in determining the mechanism subject to the number of electrons involved and the pH of the electrolyte solution.^[^ ^268^ ^]^ Thus, the number of intermediate species is indicative of the overall efficacy of the electrode and reflects on FC performance. The percentage of hydrogen peroxide (H_2_O_2_) and peroxide (HO_2_^−^) in acidic and alkaline medium, respectively, is determined using RRDE due to its sensitivity toward intermediate species. Figure 9IIa–d shows ORR–LSV curves used to obtain the disk and ring current densities and their corresponding number of electrons and the % H_2_O_2_ formation with varied potential for Pt/MWCNT and other respective bimetallic counterparts. RRDE was used to obtain polarization curves which recorded the disk current (*I*_D_) from the disk electrode surface that was generated when the oxygen species were reduced, and the reduced oxygen species being oxidized at the ring electrode to obtain the ring current (*I*_R_) and plotted as a function of the disk electrode. RRDE–LSV curves were obtained at a ring potential of 1.2 V versus RHE which was sufficient for the complete oxidation of H_2_O_2_ intermediates while reaching the centrifugal flow of the electrode rotation and adequate to ensure that peroxide formation was diffusion limited.^[^ ^269^ ^]^ Based on the disk and ring current density, %H_2_O_2_ and the number of electrons transferred are determined using the equations shown below.

(45) H2O2(%)=(2JRN)(JD+JR)/N 

where *J*_R_ is the ring current density, *J*_D_ is the disk current density, and *N* as the RRDE collection efficiency and

(46) n =4×JD(JD+JR)/N

where *n* is the number of electrons transferred and *N* is the RRDE collection efficiency, which is a fraction of the O_2_ produced on the disk that is collected on the ring for a specific geometric surface area of the disk and ring electrodes.

(47) N=−JRJD

Collection efficiency is determined using standard ferrocyanide/ferrocyanide ([Fe(CN)_6_]^3−/^[Fe(CN)_6_]^4−^) redox couple reaction with supporting electrolyte solution. The reversible redox reaction of Fe^3+^/Fe^4+^ which is a one‐electron process facilitates the estimation of the collection efficiency (N). An N_2_‐saturated solution of 0.1 m KOH or 1 m KNO_3_ with 10 mM K_3_[Fe(CN_6_)] was used as the electrolyte during the recording of the polarization curves under hydrodynamic conditions and the potential window ranged between 0.4 and 1.5 V versus RHE at a ring electrode potential of 1.55 V versus RHE. The electrode speed was also maintained between 400 and 2400 rpm at 0.01 V s^−1^ during the calibration of the collection efficiency.^[^ ^270^ ^]^

### 9.2 Testing Protocols for Benchmarking AOR Activities in Fuel Cell Electrocatalysis

To advance our knowledge of catalyst systems, future research should focus on how different testing protocols map the catalytic activity, the impact of NP size, the nature of support, and the entangled relationship between different synthesis. Comparing different nanomaterials which are proposed and screened as electrocatalysts to commercial and standard catalyst materials is also of great importance; hence, a surfactant‐free synthesis method can be used to perform such studies with an example of EOR.^[^ ^271^, ^272^ ^]^

**Figure 10:**
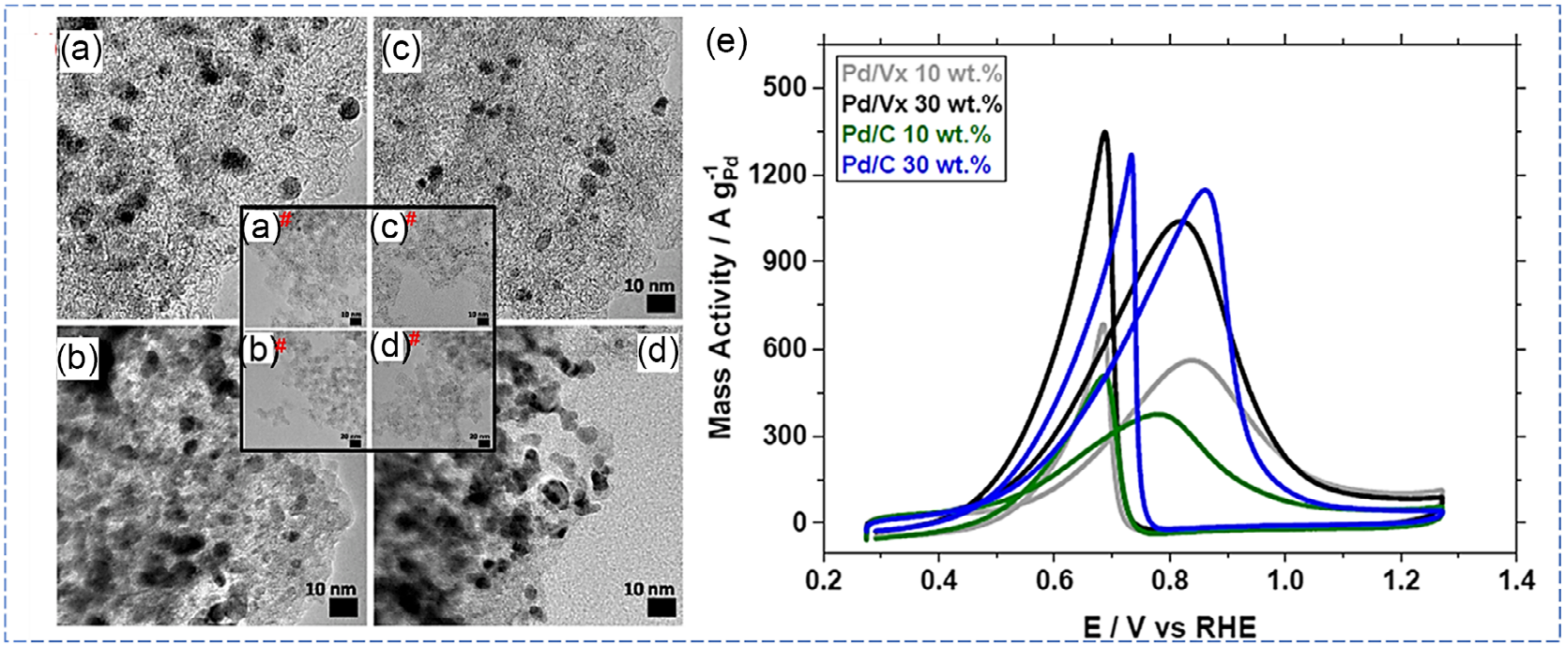
a–d) Transmission electron microscopy after chronoamperometry; inset a–d)^#^ before chronoamperometry for Pd NP on Vulcan carbon. e) Cyclic voltammograms of Pd NPs supported on different supports. Reproduced with permission.^[^ ^273^ ^]^ Copyright 2020, Elsevier.

EOR electrodes were developed by loading Pd on Vulcan (Vx) carbon. After being subjected to chronoamperometry, the nanosized Pd catalysts increased in size. **Figure** **10**a–e shows the relative as‐prepared NPs (a)^#^–(e)^#^, and the as‐prepared Pd/V*x* NPs show less agglomeration or aggregation on the support material and more defined size. These results substantiate the higher stability and long‐term mass activity for the EOR in alkaline conditions for the Pd/V*x* NPs in comparison to the commercial Pd/C. CV for the EOR in 1 m KOH alkaline conditions with 1 m ethanol was performed for Pd/V*x* 10 or 30 wt% and commercially available Pd/C 10 or 30 wt%, as shown in Figure 10e. An oxidation peak around 0.8 V versus RHE corresponds to the oxidation of ethanol on the Pd surface obtained for the forward scan (Figure 10e), which refers to the oxidation of ethanol from low potential to high potential. At high potential, the current drops due to the formation of palladium oxide. Catalysts prepared at 10 wt% Pd show low activity relative to those with high surface per mass unit.^[^ ^273^, ^274^ ^]^ These results highlight the struggle in comparing different materials since comparing synthesized catalysts with commercial catalysts poses a challenge toward understanding the activity loss over time. The production and post‐treatment method for commercial catalysts is often not stated and using commercial catalysts from different suppliers can complicate systematic comparison by impairing a clear understanding of the differences observed. For example, mass loss of Pd NPs can be directly linked with the following mechanism which is the poisoning of the commercial catalysts versus poisoning together with a marked size increase for the synthesized catalysts. The optimal size for Pd NPs design for the EOR may not be the smallest achievable size since all Pd NPs reach a size of ≈ 5–7 nm regardless of loading use and NP size after electrochemical assessment. We note from the above that there is complexity in 1) which loading should be used when synthesizing electrocatalysts, 2) commercial catalysts manufacturing should disclose their synthesis methods so that researchers benchmark with closely related methods, 3) activity loss by PGM dissolution has no well‐elaborated route of analysis, 4) there is no optimal size of the electrocatalysts, 5) also there is no set electrolyte concentration for electrocatalyst testing, and 6) in CV there is no standard potential range that is recommended in which the potential range greatly affects the peak currents.

The different electrochemical testing protocols include the activation procedure or longer tests, to understand the effect of the ink composition and different electrolytes used, and to study and optimize the electrocatalytic activity and stability for the AOR.

Alcohol electro‐oxidation is referred to as a multielectron reaction, which produces a variety of carbonaceous materials with carbon monoxide as the prominent intermediate that is believed to adsorb on the catalytic surface and impede the electrocatalytic performance. CO poisoning has been indexed by the oxidation peak in the cathodic scan of the CV, with the intensity ratio (*J*_f_/*J*_b_) being used as a measure of the CO tolerance; the higher the value, the better the tolerance. *J*_f_/*J*_b_ is the intensity ratio between the peak current in the anodic forward scan (*J*_f_) versus the peak current in the cathodic backward scan (*J*_b_). The criterion is based on the following assumptions. 1) The anodic current beyond methanol oxidation of *J*_f_ comes to fruition because of the oxidation of the adsorbed CO to CO_2_. 2) Increasing the anodic switching potential in CV weakens *J*_b_.^[^ ^275^ ^]^

In contrast to the above information, Tong et al.^[^ ^276^ ^]^ reported the validity of the criterion for methanol oxidation on Pt/C and PtRu/C electrodes in acidic solutions. In the study, they proposed that *J*_b_ and *J*_f_ oxidation peaks originated from the oxidation of surface‐adsorbed methanol and the peak intensity ratio was an inaccurate parameter for measuring CO tolerance. They explained from in situ surface infrared spectroscopy that both oxidation peaks presented opposite correlations with the amount of methanol adsorbed on the electrode surface but not that of the CO adsorbed on the catalyst surface.

**Figure 11:**
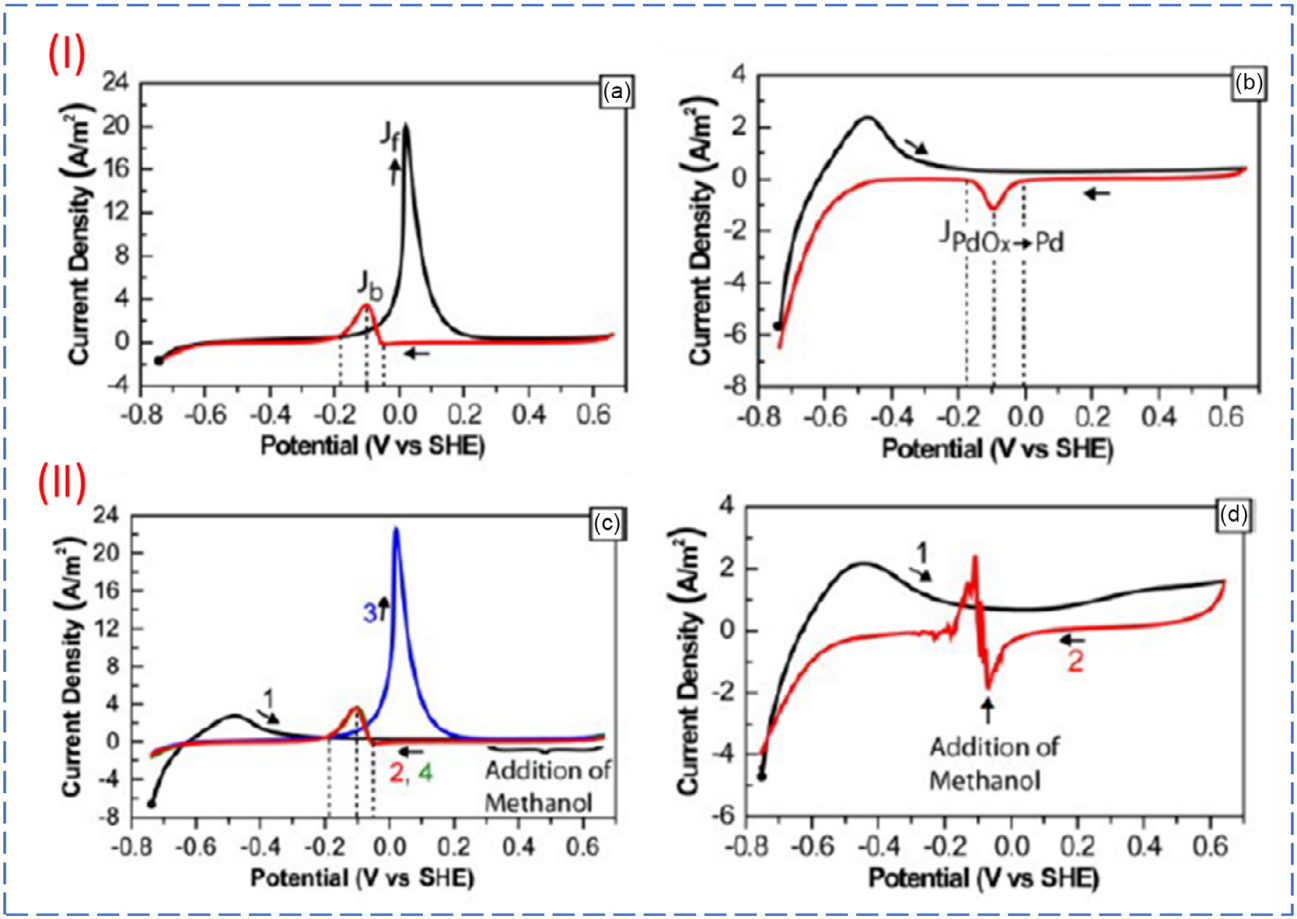
Study of the origin of the oxidation peak (*J*_b_) by polarization curves through methanol oxidation on the Pd surface. (I) a) methanol oxidation in a solution of 0.5 m NaOH + 1.0 m CH_3_OH, b) solution of 0.5 m NaOH alone, (II) c) the online made solution of 0.5 m NaOH + 1.0 m CH_3_OH by adding 10.0 mL of 2.0 m CH_3_OH/0.5 m NaOH into 10.0 mL of 0.5 m NaOH solution ran in the indicated potential window, and d) the online made solution of 0.5 m NaOH + 1.0 m CH_3_OH by adding 10.0 mL of 2.0 m CH_3_OH/0.5 m NaOH into 10.0 mL of 0.5 m NaOH solution within the specified potential range. Reproduced with permission.^[^ ^278^ ^]^ Copyright 2016, Royal Society of Chemistry.

To clarify the origin of *J*_b_, CV of methanol oxidation on a polycrystalline Pd electrode in alkaline solutions, as shown in **Figure** **11**a,b, was used for this study. Two CV curves of polycrystalline Pd electrode were documented, one in 0.5 m NaOH + 1.0 m CH_3_OH featured a large *J*_f_ and a small *J*_b_ while the other had a CV curve taken from 0.5 m NaOH without CH_3_OH, as shown in Figure 11I, as two observations were made. The first observation was elucidated to a decrease in current at a potential beyond *J*_f_ to the oxidation of Pd, which results in a loss in activity, and the second observation was linked to the onset potential of the CV of the Pd electrode in 0.5 m NaOH + 1.0 m CH_3_OH, being the same with a CV counterpart of the Pd electrode in 0.5 NaOH only.^[^ ^277^ ^]^

The above observation shows that the origin of *J*_b_ is from the oxidation of fresh methanol and reactivation of the Pd electrode surface by reduction of PdO_*x*_. This is proven by the CV curves from Figure 11II, which were taken before and after the addition of methanol to the electrolyte solution. The first anodic scan shown as line 1 in Figure 11IIc was taken in 0.5 m NaOH solution and confirms the normal behavior of Pd. The second anodic scan shown as line 2 was taken by adding equal volumes of 0.5 m NaOH + 2.0 m CH_3_OH solutions and during the potential window of adding methanol on the second scan, there was no generation of CO from the oxidation of methanol within the instrument sensitivity. On an onset potential of *J*_PdOx→Pd_ (−0.03 V), an oxidation wave burst occurred and peaked at −0.10 V. In the subsequent anodic scans shown in lines 3 and 4, the J and E profiles were identical to the one in Figure 11I. In the second case, the addition of methanol was only made after the *J*_PdOx→Pd_ emerged halfway, as shown in Figure 11IId, and an interesting feature was that the cathodic peak was immediately converted to the anodic peak upon introduction of the alcohol to the electrochemical cell.^[^ ^278^ ^]^

The above results show that J_PdOx→Pd_ is responsible for the occurrence of *J*_b_. *J*’_b_ current obtained from the oxidation of fresh methanol (*J*’_b_ = *J*_b_ + *J*_PdOx→Pd_) is greater than the apparent *J*_b_ current, and the *J*_PdOx→Pd_ process is not overpowered by the *J*’_b_ process but rather is hidden during CV measurements. There is no oxidation current at the anodic scan even if the cathodic potential is switched before the occurrence of *J*_PdOx→Pd_, which means that the oxidized surface on the Pd electrode is unable to oxidize CH_3_OH, as shown by the zero anodic current beyond 0.36 V and between 0.66 and 0.0 V in the cathodic scan as shown in Figure 11II. The conclusion made does not conflict with the generation and tolerance of the CO which is generated during the forward scan and even though there is a small contribution of CO oxidation in *J*_b_ during the repeated CV scans in the presence of CH_3_OH, the effect of it is minimal and below the instrument detection range.^[^ ^176^ ^]^

## 10 Key Parameters for Improving Performances of Electrocatalysts for Direct Alcohol Fuel Cells

The three significant parameters for an efficient electrocatalyst are 1) higher electrical conductivity, 2) favorable electrocatalytic activity, and 3) longer‐term stability at greater temperatures or over a broader pH environment. The first component is critical for reducing Ohmic loss and increasing electrocatalytic efficiency. To avoid a drop (degradation) in overall performance, the comparatively high conductivity of the electrocatalysts must not prevent electron transmission while still increasing the number of active sites. The quick and efficient charge transfer caused by the carbon matrix's conductivity, as well as the micropores for exposing the active sites, have been credited as the unique attributes of the electrocatalysts for efficient performance and stability under electrochemical conditions. For example, in an aqueous environment, a family of transition metals (Ni, Fe, and Co) N‐doped porous C electrocatalysts for energy conversion systems have been demonstrated to display increased activity and selectivity following the sequence Ni, FeCo, and Ni > FeCo.^[^ ^279^ ^]^

Tripkovic et al.^[^ ^280^ ^]^ proposed a theory that indicated the impact of the metal center. The author demonstrated that porphyrin‐like metal hybridized with graphene might be active for the energy conversion process, allowing the transformation of intermediate to other valuable products. The distinctive functions of metal centers have been suggested by several electrocatalysts with diverse metal centers.^[^ ^279^, ^281^, ^282^, ^283^, ^284^, ^285^, ^286^, ^287^, ^288^, ^289^, ^290^, ^291^ ^]^

Moreover, another key technique for a positive electrocatalytic activity includes boosting intrinsic activity and increasing the number of accessible active sites, for example, increasing catalyst loading and tweaking the electrocatalyst nanostructure. It is necessary to evaluate the realistic limit of electrocatalyst loading without obstructing other critical parameters (mass and charge transfer). This is because only when the mass loading reaches the monodispersed surface do performances improve. Sintering and agglomeration would occur if the electrocatalyst mass loadings exceeded this level, which would be detrimental to electrocatalyst dispersion and performance. The effect of material percentage on electrocatalytic performance has been described,^[^ ^292^, ^293^, ^294^ ^]^ indicating that the content and architecture of the material are significant in building an efficient electrocatalytic for DAFCs.

In order to improve the efficiency of electrocatalysts, reaction conditions (such as pH and temperature) must be studied.^[^ ^292^, ^295^, ^296^, ^297^, ^298^, ^299^, ^300^ ^]^ The influence of heat treatment (temperature) on the structural properties of the catalyst activities and selectivity toward DAFCs should be considered. The FE and product selectivity are influenced by pH and local pH.^[^ ^301^, ^302^, ^303^ ^]^ Electrolysis at varied pH values can impact the pH on the selectivity toward DAFCs, which further revealed the important functions of proton concentrations.^[^ ^304^ ^]^

Furthermore, the impact of metal precursors on the alcohol adsorption onto the active center on overall electrocatalytic activity cannot be overlooked. This is because the greater metal carrier interactions between a well‐dispersed metal monoatomic and carbon substrate(s) can modify the geometrical configuration and electronic structures of the catalytically active site as well as block metal atom aggregations/association.^[^ ^305^, ^306^, ^307^, ^308^, ^309^ ^]^ It should be emphasized that a variety of parameters influence the performance of electrocatalysts for DAFCs; nevertheless, electrocatalyst architectures, compositions, and process conditions remain common considerations.

## 11 Breakthroughs on the Horizon

Electrocatalysts for DAFCs are fast developing study topics due to rising global demands and adequate electrocatalysts providing cheap and renewable energy. The manufacture of electrocatalysts and their uses in DAFCs has accelerated in the previous decades. Many catalysts have been reported to be durable, selective, and efficient, needing the attention of enterprises hoping to profit from the growing renewable energy markets. As a result, the present research suggests that the best method to make a substantial breakthrough on the horizon in the sector is to push the limits of catalyst materials for DAFC capabilities.

To integrate the electrocatalytic set‐ups for homogenous and heterogeneous processes, there is indeed a necessity for feasible intelligent/smart modifications of convectional analysis and spectroscopic methods. These would be anticipated to provide a comprehensive and concise understanding of the chemical characteristics of the process intermediate. The operando investigation can be adopted to understand the mechanistic features that could be used to enable significant custom fabrication modifications/designs. Although it is recognized that such a strategy can take quite some time, it does have a better probability of succeeding than the usual random trials and errors. As a result, despite significant success over the years on DAFC electrocatalysts, the future involves innovation or the development of such a novel interception‐based method.

To achieve precise and practical DAFCs applications on an industrial scale, the microscopic forms of electrocatalysts are usually disregarded. Several academic studies on these electrocatalysts have mostly concentrated on powdered forms, which are rarely appropriate for commercial use. This is due to the dustiness of the fluid dynamic applications and the inevitable pressure drop(s). As a result, palletization is probably one of the best and simple procedures. However, increased pressure is required during the palletization operations, which might potentially degrade or modify the pores of the electrocatalysts. Therefore, selecting a suitable binder and mechanical condition is critical for an efficient FC application.

## 12 Conclusions, Perspectives, and Outlooks

This review summarizes the advancement in doping engineering of electrocatalysts for ORR and AOR, especially types of doping and corresponding electrocatalytic mechanism. Heteroatomic dopants and metallic dopants have proven to enhance electrocatalysts' electronic activity and structural stability in FC applications. Doping is a promising development for affordable and practical electrocatalysts for anodic oxidation and cathodic reduction in FCs. The introduction of heteroatomic and metallic dopants effectively influences the interface properties and electronic structure of metal‐based and nonmetal‐based electrocatalysts. Thus, managing the adsorption energy of poisonous intermediates by disorganizing the distribution of charge and generating a new charge balance thereby speeding up the kinetics of the electrocatalytic reactions is needed. Although in recent years remarkable progress has been archived, challenges and a big research gap still exist, and these need to be apprehended to produce more efficient electrocatalysts for both ORR and AOR. For the timeline surpassing the last decade, there has been tremendous research in developing innovative nanomaterials for use as electrocatalysts in FCs. The innovative nanomaterials roles have increased the electrocatalytic kinetics for anodic and cathodic reactions, AOR and ORR. For anodic reactions, the main goal is to get electrocatalysts that can cleave the carbon‐to‐carbon bonds in monohydric (ethanol, etc.) and polyhydric (ethylene glycol and glycerol, etc.) alcohols. Also, the smart nanomaterials should be able to weakly adsorb intermediates or completely do away with the intermediaries, directing complete electrocatalytic oxidation to carbon dioxide and releasing the maximum possible number of electrons. For the cathodic reaction, the smart nanomaterials design has been for them to direct the ORR for the four‐electron pathway, shunning the two‐electron pathway that produces hydrogen peroxide, with slow kinetics. Majorly, smart nanomaterials provide the reaction sites that enhance and necessitate the FOR and the electroreduction of oxygen in FC operation. Highly innovative strategies in nanotechnology and materials engineering are needed to realize widespread usage of FCs worldwide; however, despite the great strides made in developing electrocatalysts and mapping the electroctrocatalytic routes, there are still vast challenges that need continued attention to improve and broader exploration of new innovative electrocatalysts. More effects need to be accomplished in the following aspects.

Optimization of electrocatalytically active sites: Active sites range from defects and metallic centers to nonmetallic centers. The progression of the electrochemical reactions has distinct energy barriers and is primarily dependent on these sites. Thus, new optimization and control methods are needed to engineer the electroactive sites to maximize the exposure and correlation between the metallic site and defects.

Elucidation of mechanistic pathways: Different classes of electrocatalysts facilitate the AOR and ORR in different ways. Research should focus on determining if the active sites change or reconstruct during electrocatalysis. This will create a deep understanding and instigate a well‐informed proof of concept for the electrocatalysts design that is highly active, selective, stable, and durable.

In situ and ex situ advanced operando characterizations: The electrochemical data, theoretical insights, and accurate in situ measurements should be compounded. This coupling will help have a more interrelation of the various electrochemical processes. Collectively, new theoretical models will be developed, bringing to light the metal–support interaction related to enhancing electroactivity and durability.

Robust electrocatalyst designs (structure–property relationship), interface optimization, unambiguous mass, and charge transport for the propulsion of different mechanisms, for example, bifunctional, intrinsic, inner sphere, outshpere mechanisms, etc.

The relationship between heteroatomic dopants and how they enhance catalytic activity in ORR and AOR should be clarified further. Where N is used as a dopant optimal ratio of graphitic N, pyridinic N should be determined. Effective techniques should be explored on how to efficiently dope the active dopants in a controlled manner into the carbon skeleton. The spark plasma sintering technique can be used as it is known to dope heteroatoms into the carbon skeleton effectively and produce high content of heteroatoms inside the doped carbons. This will be useful in exploring and characterizing the connection between the arrangement of dopants and their catalytic performance.

The development of other novel technologies used for the characterization of dopant configurations in carbons should be explored other than XPS.

The functions of different arrangements of doped elements should be analyzed as this can bring new avenues in the synthesis and design of highly effective ORR and AOR electrocatalysts.

Solid experimental proof and explanations of the synergistic effect of multiheteroatom doping should be elucidated since the use of multiheteroatom doping has been reported to demonstrate great effects on the electronic structure which improves their electrocatalytic activity. The achievement of precise control, characterization, and synthesis of the specific arrangement of dopants in multidoped carbons should be discussed.

New theoretical approaches and experimental methods should be developed to dictate the location and structure of the active sites on the electrocatalyst surface to enhance the ORR and AOR.

An understanding of the atomic‐level doping state and atomic structure of heteroatoms is still undefined. Hence, catalytic activity should be studied at the atomic level to design high‐performance electrocatalysts. The use of electron microscopy and spectroscopy will aid in the precise analysis of the active site structure of the electrocatalysts which will lead to the synthesis of efficient electrocatalysts at an atomic scale.

The interlinkage between dopants and catalysts should be explored. A more detailed evaluation of the factors that influence catalytic performance should be considered. These include electron configurations, electrical conductivity, and surface area.

Heteroatomic doping and metallic doping deserve constant attention. Through linking various experiments and calculations, a concise understanding of the relationship between doping and FC performance can be reached. Such an understanding gives initiatives for the design of electrocatalysts and gives insight into the electrocatalysts for ORR and AOR to improve FC performance. Strategies should be put in place for the large‐scale synthesis of heteroatom and metal‐doped electrocatalysts for FCs to meet the economic demands of a FC application. Current methods yield electrocatalysts which are very low and only possible in the laboratory. Scaling up the synthesis methods is difficult due to the high‐energy processes involved during synthesis. Therefore, it is urgent to develop novel and effective methods for large‐scale synthesis of these electrocatalysts.

## Conflict of Interest

The authors declare no conflict of interest.
